# Quantitative iTRAQ-based proteomic analysis of phosphoproteins and ABA-regulated phosphoproteins in maize leaves under osmotic stress

**DOI:** 10.1038/srep15626

**Published:** 2015-10-27

**Authors:** Xiuli Hu, Nana Li, Liuji Wu, Chunqi Li, Chaohai Li, Li Zhang, Tianxue Liu, Wei Wang

**Affiliations:** 1 State Key Laboratory of Wheat and Maize Crop Science, Collaborative Innovation Center of Henan Grain Crops, Henan Agricultural University, Zhengzhou, China

## Abstract

Abscisic acid (ABA) regulates various developmental processes and stress responses in plants. Protein phosphorylation/dephosphorylation is a central post-translational modification (PTM) in ABA signaling. However, the phosphoproteins regulated by ABA under osmotic stress remain unknown in maize. In this study, maize mutant *vp5* (deficient in ABA biosynthesis) and wild-type *Vp5* were used to identify leaf phosphoproteins regulated by ABA under osmotic stress. Up to 4052 phosphopeptides, corresponding to 3017 phosphoproteins, were identified by Multiplex run iTRAQ-based quantitative proteomic and LC-MS/MS methods. The 4052 phosphopeptides contained 5723 non-redundant phosphosites; 512 phosphopeptides (379 in *Vp5*, 133 in *vp5*) displayed at least a 1.5-fold change of phosphorylation level under osmotic stress, of which 40 shared common in both genotypes and were differentially regulated by ABA. Comparing the signaling pathways involved in *vp5* response to osmotic stress and those that in *Vp5*, indicated that ABA played a vital role in regulating these pathways related to mRNA synthesis, protein synthesis and photosynthesis. Our results provide a comprehensive dataset of phosphopeptides and phosphorylation sites regulated by ABA in maize adaptation to osmotic stress. This will be helpful to elucidate the ABA-mediate mechanism of maize endurance to drought by triggering phosphorylation or dephosphorylation cascades.

Drought is one of globally environmental stress that greatly hampers crop production. The frequent occurrence of drought with rising temperature poses a serious challenge to sustainable crop production^1^. Maintaining crop yield stability in a changing climate is needed to guarantee a food supply for the increasing world population. Particularly, maize (*Zea mays* L.), one of the major food crops globally, is often exposed to drought stress. So, improving maize for increased drought tolerance is a priority in breeding programs^2^.

At the molecular level, understanding the mechanism of crops response to drought is useful to develop genotypes with improved drought tolerance^3^. Most known regulatory genes, e.g., transcription factors (TFs) and protein kinases, are characterized as important stress regulators based on their transcriptional induction by various stresses. However, many proteins are biologically active *in vivo* only after undergoing some kind of post translational modification (PTM), e.g., WRKY4 and ZmCPK4^5^. For example, protein phosphorylation plays a critical role in regulating many biological functions including stress endurance through signal transduction^6^. Many regulatory proteins and enzymes can be switched on and off by phosphorylation and dephosphorylation to control a wide range of cellular processes or signal relays^2^. In maize response to drought stress, 138 phosphopeptides display highly significant changes and their corresponding proteins affect epigenetic control, gene expression, cell cycle-dependent processes and phytohormone-mediated responses^6^; in bread wheat response to drought stress, 31 phosphoproteins have significant change of phosphorylation level and are mainly involved in three biological processes: RNA transcription/processing, stress/detoxification/defense, and signal transduction^7^. Moreover, previous studies also indicate that there are different phosphoprotein changes in different crops response to drought stress. Thus, characterizing protein phosphorylation and its dynamics in cell response to stresses will contribute to understanding signaling pathways and stress endurance mechanisms in crops.

Plant hormone abscisic acid (ABA) is involved in regulating several major processes, such as seed dormancy, germination and seedling growth, and various stress responses. ABA can regulate different sets of stress-responsive genes to initiate the synthesis of various proteins, including TFs, enzymes, and molecular chaperones^8^. Protein phosphorylation belongs to a type of rapidly PTMs in the ABA-regulated signaling pathway^7^. ABA-regulated phosphoproteins have been analyzed in Arabidopsis^9^,^10^,^11^,^12^ and rice^10^,^13^,^14^. However, it remains unknown whether *in vivo* phosphosites of many drought stress-responsive protein kinases are involved in ABA-triggered maize response to drought stress. Recently, iTRAQ-based quantitative proteomic and LC-MS/MS methods demonstrate the power of quantitative analysis for protein phosphorylation. Using these methods, a total of 1625 unique phosphopeptides have been detected from 1126 phosphoproteins in soybean root hairs, of which 273 phosphopeptides corresponding to 240 phosphoproteins are significantly regulated in response to *Bradyrhizobium japonicum*^15^.

Maize *viviparous-5* (*vp5*) is deficient in ABA biosynthesis^16^,^17^, with much reduced ABA content in seeds, roots and leaves compared to its wild-type *Vp5*. Thus, the mutant *vp5* and wild-type *Vp5* are useful for the studies of ABA-regulated phosphoproteins in maize. In this study, multiplex run iTRAQ-based quantitative phosphoproteomic analysis and LC-MS/MS methods were performed to identify and compare the differential phosphoproteins in maize under osmotic stress. As a result, up to 4052 unique phosphopeptides, corresponding to 3017 phosphoproteins, were identified, and their phosphorylation levels were analyzed as ABA-dependent or independent.

## Results

### Differentially accumulated phosphopeptides under osmotic stress

**Figure 1:**
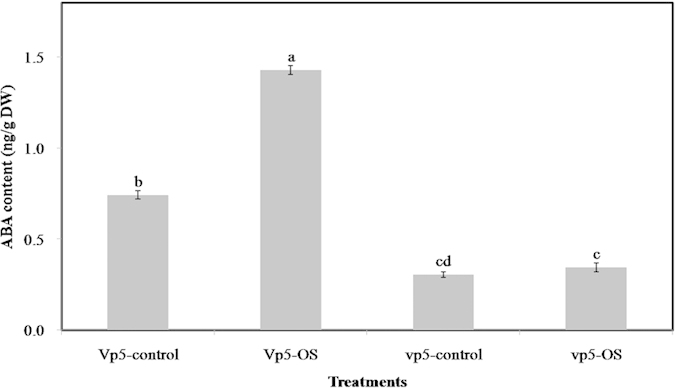
ABA content in maize ABA-deficient mutant *vp5* and wild-type *Vp5* leaves under normal conditions (control) or 8 h osmotic stress (OS). Values are means ± SE (n = 5).

The ABA content in *vp5* and *Vp5* leaves was measured by ELISA. Under osmotic stress, the increased ABA content in *Vp5* and *vp5* leaves was 0.6863 and 0.0403 ng/g · dry weight, respectively; the increased ABA content in *Vp5* leaves was about 17 times that in *vp5* leaves (Fig. 1). This difference in ABA accumulation facilitates the study of the ABA-regulated signaling pathways in maize exposed to osmotic stress.

**Figure 2:**
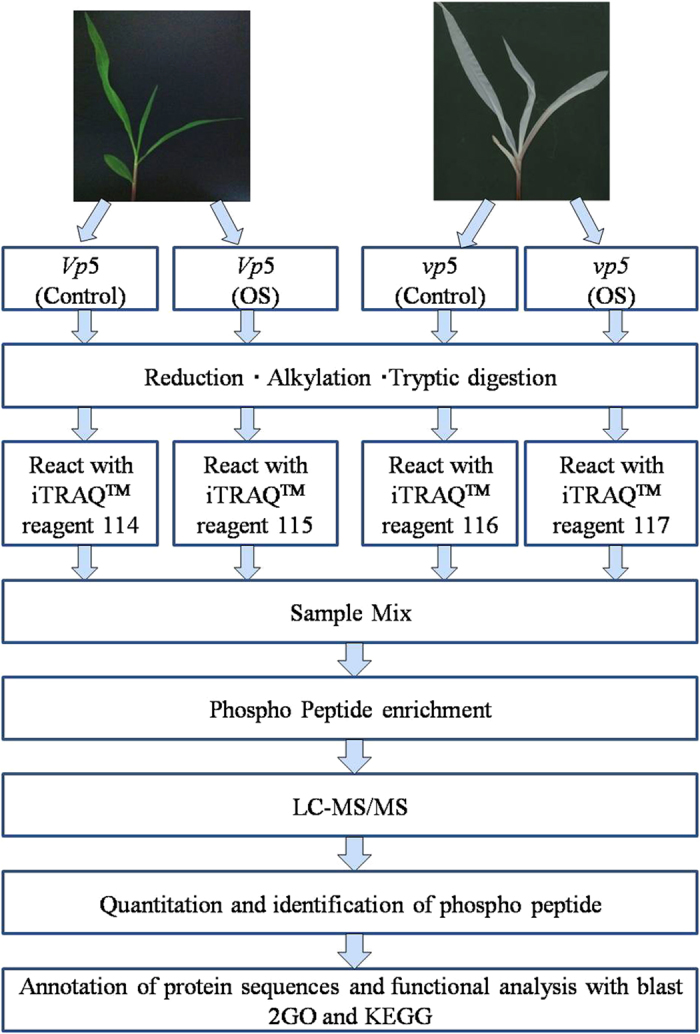
iTRAQ 4-plex labeling and LC MS/MS workflow of identifying phosphorous proteins in leaves of maize ABA mutant *vp5* and wild-type *Vp5* seedlings under osmotic stress (OS).

**Figure 3:**
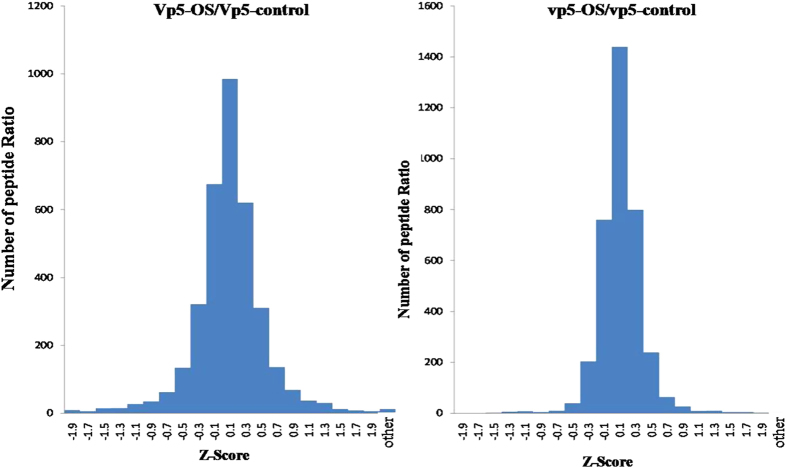
Z-scores frequency distribution of differential peptides in maize wild type *Vp5* and mutant *vp5* under osmotic stress. iTRAQ ratios between osmotic stress (OS) and controls for each run were converted to z-scores to normalize the data. Positive z-score values represent proteins up-regulated by OS and negative values represent proteins down-regulated by OS. Z-scores between −0.9 and 0.9 indicates proteins not significantly altered, between ±0.9 and 1.96 moderately altered, and ≥1.96 and ≤−1.96 significantly altered ≥2-fold during osmotic stress (>95% confidence).

Total leaf proteins from *vp5* and *Vp5* seedlings exposed to osmotic stress were isolated and analyzed as shown in the work flowchart (Fig. 2). Simultaneously, osmotic stress and control iTRAQ ratios for each run were converted to z-scores to normalize the data (Fig. 3), resulting in the identification of 4052 unique phosphopeptides (correspond to 3017 proteins) at a false discovery rate (FDR) of 5%. Among the 4052 unique phosphopeptides, 53.84% contained only a single phosphoryl group, 37.81% contained two, 7.34% contained three, and 1.03% contained four and above. At a FDR of 1%, there were 3240 phosphorylated peptides and 153 non-phosphorylated peptides; the ratio of phosphoenrichment was 95.49%. At a FDR of 5%, there were 4052 phosphorylated peptides and 221 non-phosphorylated peptides; the ratio of phosphoenrichment was 94.84%.

**Figure 4:**
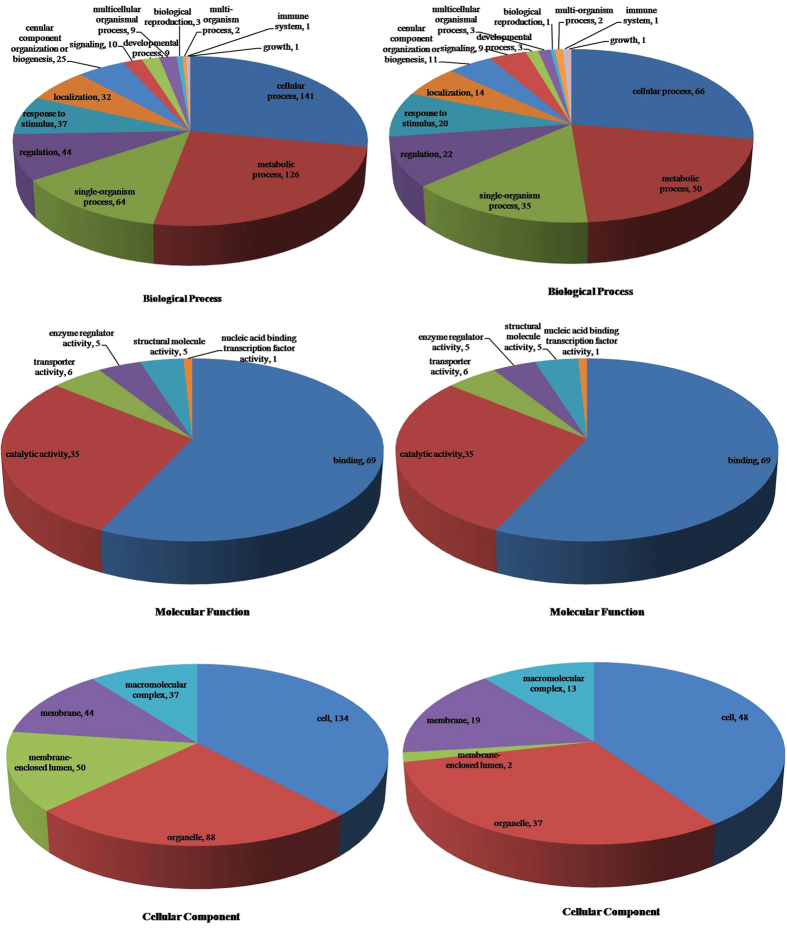
The distribution of differentially phosphorylated proteins in maize response to osmotic stress. The 160 proteins identified were classified according to their known or predicted cellular component, molecular function, biological process, and signaling pathway. Left, *Vp5*; right*, vp5*.

The proteins corresponding to the identified phosphoryled peptides in *vp5* and *Vp5* exposed to osmotic stress were annotated using Blast2GO according to the cell component and biological and molecular function (Fig. 4).

Concerning cell component, 308 and 119 phosphoproteins were annotated in *Vp5* and *vp5*, respectively, showing an unbiased distribution in different compartments. Thus, no protein enrichment procedure was introduced during protein extraction.

Concerning the biological process, phosphoproteins corresponding to the identified phosphopeptides in both genotypes were classified into 14 categories. The top categories with the highest number of phosphoproteins were cellular processes (28% in *Vp5* and 27.72% in *vp5*), metabolic processes (25% in *Vp5* and 21% in *vp5*) and single organism processes (12.70% in *Vp5* and 4.96 in *vp5*), and these three functional categories were the most important in maize response to osmotic stress.

Concerning the molecular function, phosphoproteins corresponding to the identified phosphopeptides in both genotypes were classified into 9 categories. The top 3 categories with the highest number of phosphoproteins were transcription factor activity (57.57% in *Vp5* and 57.24% in *vp5*), catalytic activity (28.95% in *Vp5* and 28.92% in *vp5*) and transporter activity (4.28% in *Vp5* and 5.95% in *vp5*).

**Figure 5:**
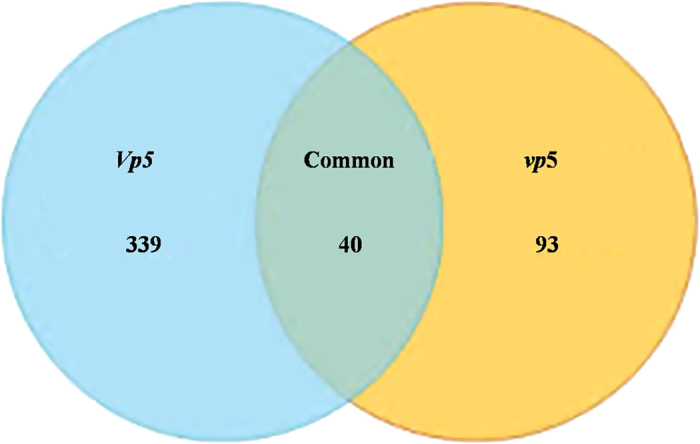
Venn diagram showing the number of proteins with significant changes of phosphorylation levels in maize *vp5* and *Vp5* leaves exposed to osmotic stress.

Of the 4,052 phosphopeptides identified, there were 379 and 133 phosphopeptides with ≥1.5 folds (increased) or ≤0.6 folds (decreased) only in *Vp*5 and *vp*5, respectively, 40 in both genotypes (Fig. 5). This change was equivalent to a significant expression ratio according to the standard with p-value <0.05 (Table 1, Tables S1 and S2). These significant phosphopeptides corresponded to 472 phosphoproteins. In order to further test the significance of 512 phosphopeptides, FDR attained by Benjamini-Hochberg method at 5% level were used to adjust p-values (correction for multiple comparisons). As a result, 36 phosphopeptides were no significant difference, which corresponded to 36 phosphoproteins, including C0PLA9 and B8A0C6 (Table 1) and other 34 listed in Table S1. Among the 36 phosphoproteins, other phosphopeptides of B4F8Q3, B4FZY1, B6TDL6 and Q9ATM4, were significant (Table 1, Tables S1 and S2).

In order to prove that the observed changes in phosphopeptide abundances were due to the changes in phosphorylation state or the abundance change, protein abundance was also measured using the iTRAQ technique. As a result, among 472 phosphoproteins, 187 phosphoproteins changed in abundance but in no significant level; 10 phosphoproteins changed in abundance with a significant level only in *Vp*5 or *vp*5; no changes in abundance of the rest 275 phosphoproteins were detected (Tables S3–S5). Except C0P8S9 and K7U4E0, eight (B4G1E6, B6T0F0, B6STN4, B6T6R3, B6TPC9, K7VBH0, B6TM56 and K7UCK7) of the 10 differential abundance phosphoproteins resulted from a significant change in phosphorylation state. For example, B4G1E6 had significant phosphopeptide abundances but no significant protein abundances in *Vp*5, whereas it had significant protein abundances but no significant phosphopeptide abundances in *vp*5; K7UCK7 had significant folds of phosphopeptide only in *vp*5 which existed significant difference compared to protein abundances (Table S4).

Furthermore, for the 379 phosphopeptides in *Vp5*, the numbers of phosphoRS sites at S, T and Y residues were 585 (56.41%), 194 (33.16%) and 61 (10.42%), respectively. For the 133 phosphopeptides in *vp5*, the numbers of phosphoRS sites were 181 (51.93%), 66 (36.46%) and 21 (11.60%), respectively. For each peptide, the PhosphoRS site probabilities above 75% indicate that a site is truly phosphorylated (Table 1, Tables S1 and S2).

Our data showed that many phosphoproteins were differentially phosphorylated and involved in a series of DNA/RNA-related processes and protein synthesis/degradation (Table 2). This was consistent with the results attained by using Blast2GO software to analyze the biological function, cellular components and molecular function (Fig. 4).

### Phosphorylation motifs in phosphopeptides

To determine whether the phosphorylated versions of the identified phosphopeptides had different phosphorylation site motifs in both genotypes and whether ABA affected the motifs, Motif-X online software was used to predict the motif specificity of the phosphopeptides. The motifs SP and TP were common in both genotypes response to osmotic stress; 12 motifs were only predicted in *Vp5*; 2 motifs were only predicated in *vp5* (Table 3). These results indicated a high sensitivity and specificity of phosphorylation sites in maize response to ABA under osmotic stress.

In the present study, 34 phosphoproteins (Table 4) were found to contain several phosphopeptides. Notably, these peptides had specific phosphorylation characteristics in response to ABA and osmotic stress. Particularly, the phosphorylation level of two different peptides in 13 phosphorylation proteins was up-regulated or down-regulated in *Vp5*, whereas there was no change in *vp5* under drought stress. These results indicated that ABA regulated the phosphorylation of different peptides of one protein with contrasting influence in maize response to osmotic stress. In contrast, B4F808 and C0HF00 were up-regulated or down-regulated in *vp5*, whereas no changes were detected in *Vp5* under osmotic stress. The different phosphopeptides of the other 19 phosphoproteins had similar response to ABA under osmotic stress. Overall, this result showed the diversity of the phosphorylation sites and their specificity in maize response to ABA and stress treatments.

### Effect of ABA on peptide phosphosites regulated by osmotic stress

The mechanisms of plant response to stress include both ABA-dependent and ABA-independent processes^18^. In this study, a total 472 phosphorylation peptides were changed with an l.5-fold increase, including 40 in two genotypes, 339 only in *Vp5* and 93 only in *vp5*. Specially, among 40 phosphopeptides identified in both genotypes (Table 1), the phosphorylation level of some phosphopeptides (corresponding to protein ID: B6TE49 and K7V8B2) increased in *Vp5* but decreased in *vp5*, indicating that these phosphopeptides were up-regulated by osmotic stress in an ABA-dependent way; the phosphorylation level of some phosphopeptides (corresponding to protein ID: K7TWA4 and Q3MQ01), was not obviously different in two genotypes, indicating that they were regulated by osmotic stress in an ABA-independent way; The phosphorylation level of some phosphopeptides (corresponding to protein ID: B6SP06, K7USN0 and B6TCM5) (Table 1), increased in both genotypes, but the increase was more significant in *Vp5* under osmotic stress, indicating that they were regulated by osmotic stress in an ABA-dependent or ABA-independent way. Overall, the phosphorylation levels of 27 proteins were up-regulated by osmotic stress (9 in an ABA-dependent way, 12 in an ABA-dependent or ABA-independent way and 4 in an ABA-independent way), and 2 down-regulated by ABA; the phosphorylation levels of 13 were down-regulated by osmotic stress: 10 in an ABA-dependent way and 3 in an ABA-independent way.

Among the 339 phosphopeptides whose phosphoryltion level were identified with fold change >1.5 only in *Vp5* (Table S1), 183 were down-regulated by osmotic stress, of which 156 had significant increase folds compared to *vp*5, indicating a down-regulation in an ABA-dependent way; 27 had no significant increase folds compared to *vp*5, indicating a down-regulation in an ABA-independent way; 156 were up-regulated by osmotic stress, of which 136 had significant increase fold compared to *vp*5, indicating an up-regulation in an ABA-dependent way; 20 had no significant increase folds compared to *vp*5, indicating an up-regulation in an ABA-independent way (Table S1).

Among the 93 phosphopeptides whose phosphorylation level were identified with fold change >1.5 only in *vp5* under osmotic stress (Table S2), 34 peptides with >1.5 fold increase had significant difference in *vp5* compared to *Vp*5, indicating a down-regulation by ABA; 19 peptides with >1.5 fold decrease had significant difference in *vp5* compared to *Vp*5, indicating an up-regulation by ABA; three were down-regulated both in *vp5* and *Vp*5 but without significant difference between them, indicating a down-regulation by osmotic stress in an ABA-independent way; two were up-regulated in *vp5* and *Vp*5 but without significant difference between them, indicating an up-regulated by osmotic stress in an ABA-independent way; one was up-regulated more in *vp5* than in *Vp*5, indicating up-regulated by osmotic stress but down-regulated by ABA. Particularly, among the 93 phosphopeptides, the phosphorylation levels of 34 peptides were detected in *vp*5 but not in *Vp*5, of which 22 were significantly up-regulated and 12 significantly down-regulated under osmotic stress (Table S2).

### Phosphorylation of ubiquitin and transporters

Ubiquitin is a highly conserved protein found in all eukaryotic species. This small protein is involved in the destruction of endogenous target proteins via the ubiquitin 26S proteasome system, which is the primary proteolysis mechanism in eukaryotic cells^19^. In the present study, 11 phosphopeptides corresponding to 8 ubiquitin proteins were identified during osmotic stress (Table 2). The phosphorylation level of ubiquitin-conjugating enzyme e2 22-like (B4FHK6), e3ubiquitin-protein ligase rhf2a-like x1 (B4FAG8: eVNAGIASVsR), ubiquitin carboxyl-terminal hydrolase isozyme l5-like (C0P3H1), e3ubiquitin-protein ligase ubr7-like (B4G0Z1) and ubiquitin carboxyl-terminal hydrolase 6-like (B6T6V5) was decreased by osmotic stress in *Vp5*, whereas no obvious change occurred in *vp5*. In contrast, the phosphorylation level of e3ubiquitin-protein ligase rhf2a-like isoform x1 (B4FAG8: rHSTGQstPDR), e3ubiquitin-protein ligase upl4-like (K7V4D9), ubiquitin ligase protein cop1 (B6UEN7) and e3ubiquitin-protein ligase upl1-like (K7TFK8) was increased by osmotic stress in *Vp5*, whereas no obvious change occurred in *vp5*. These results indicated that ubiquitination played an important role in ABA regulating maize response to osmotic stress.

All types of transporters are important for turgor pressure and water-potential regulation, which is crucial to the growth and survival of plants under water stress. In the present study, the phosphorylation level of 16 transporters related to the cell ion/water-potential regulation was significantly changed (Table 2). Particularly, the phosphorylation level of aquaporin PIP2–5 (Q9ATM7) and aquaporin pip2–4-like (Q9XF58) was decreased by osmotic stress in *Vp5*, whereas no difference occurred in *vp5*; the phosphorylation level of probable sugar phosphate/phosphate translocator at3g17430-like (B6U937), vacuolar amino acid transporter 1-like (C0PEW7), hexose transporter (B6U6U2), zinc transporter (K7U2V8), solute carrier family facilitated glucose transporter member 8 (K7UMX4) and abc transporter b family member 1-like (Q6UNK5) was significantly up-regulated by osmotic stress in *Vp5*, where there was no difference in *vp5*; Na^+^/H^+^ antiporter (B4FZY1) had two different phosphopeptides whose phosphorylation level was up-regulated or down-regulated by osmotic stress in *Vp5*, whereas no difference occurred in *vp5*; the phosphorylation level changes of K^+^ efflux antiporter 5-like (B6SP24) and sodium hydrogen exchanger 6-like (B4FS09) was only detected in *vp5*. These results indicated that ABA might regulate the phosphorylation states of transporter proteins to maintain cell solute and ion homeostasis under osmotic stress.

### Phosphorylation of chloroplast proteins

*vp5* seedlings have light green leaves under dim light conditions. Nevertheless, *vp5* seedlings have white leaves under high light conditions due to photooxidation of chlorophyll^20^,^21^,^22^. *Vp5* seedlings had green leaves. This difference of morphology is helpful to identify the chloroplast-related phosphoproteins. In the present study, there were 20 chloroplast proteins corresponding to 23 phosphopeptides whose phosphorylation level was significantly changed by osmotic stress (Table 5). The phosphorylation level of 5 phosphoproteins (B6UBN4, B4FQ59, B6SVI8, B6T9S5 and C4JAR6) was significantly increased (B6SVI8: decreased) by osmotic stress in *vp5*, whereas had no significant change in *Vp5* response to osmotic stress; the phosphorylation level of 8 phosphoproteins (Protein ID: B4FAW3, B4FSE2, B4FVB8, B4FZ38, B6SS20, C0PM56, K7U926 and P22275), was significantly increased by osmotic stress in *Vp5*, whereas there was no differences in *vp5* response to osmotic stress; the phosphorylation level of the rest 8 phosphoproteins had an opposite response under osmotic stress.

### Responses of kinases and phosphatases to osmotic stress

The responses of enzymes, including protein kinases and phosphatases, are notable. In this study, 34 protein kinases/phosphatases were found to be involved in the ABA regulating maize response to osmotic stress (Table 6). The phosphorylation levels of the top 30 protein kinases/phosphatases (except B7ZYR5: atsEEERSGGtPPAAPtP) was significantly increased or decreased by osmotic stress in *Vp5*, whereas had no significant change in *vp5* response to osmotic stress; by contrast, the phosphorylation levels of cyclin-dependent kinase family protein (K7VGC6), calcium-dependent protein kinase (Q41790) and tpa: leucine-rich repeat receptor-like protein kinase family protein (B7ZYR5) was significantly increased or decreased in *vp5* response to osmotic stress, whereas had not obvious change in *Vp5* response to stress. These results showed that ABA was involved in the phosphorylation and dephosphorylation of the 34 protein kinases/phosphatases. Particularly, some phosphopeptide belonged to the same protein kinases/phosphatases but had a different response to osmotic stress. For example, the phosphorylation levels of two different peptides, vAFNDTPTTVFWtDyVATR and qLsSGAAR of the map kinase family protein isoform 1 (B8A0M9) were up-regulated and down-regulated in *Vp5* response to osmotic stress, respectively, but with no significant change in *vp5* response to osmotic stress.

### Signaling pathways regulated by ABA under osmotic stress

According to the KEGG results, signal pathways related to phosphoproteins with significant changes of phosphorylation level in *Vp5* (Table S6) and *vp5* (Table S7) response to osmotic stress were classified into 47 and 35 categories under osmotic conditions, respectively. For *Vp5,* the top 3 categories with the highest number of phosphoproteins were spliceosome (13), carbon metabolism (9) and biosynthesis of amino acids (7), RNA transport (7), and the mRNA surveillance pathway (7). For *vp5*, the top 3 categories with the highest number of phosphoproteins were spliceosome (5), the PI3K-Akt signaling pathway (4) and photosynthesis, ribosome, RNA transport, the mRNA surveillance pathway, cell cycle, and protein processing in endoplasmic reticulum (3). Particularly, 15 signal pathways, including glycolysis/gluconeogenesis, pentose phosphate pathway, glycine, serine and threonine metabolism, plant hormone signal transduction, fructose and mannose metabolism, circadian rhythm–plant, photosynthesis-antenna proteins, cysteine and methionine metabolism, glyoxylate and dicarboxylate metabolism, one carbon pool by folate, nicotinate and nicotinamide metabolism, porphyrin and chlorophyll metabolism, sulfur metabolismvaline, proteasome, protein export, lysosome and peroxisome, were only found in *Vp5*; however, meiosis–yeast, galactose metabolism, and cell cycle-yeast were only found in *vp5* (Table S7).

The top five signaling pathways in both genotypes all included spliceosome, RNA transport and the mRNA surveillance pathway. The three pathways are involved in mRNA synthesis and processing. In the present study, 27 identified proteins (K7V792, C0HIN5, B6U3A0, M1GS93, B4FUX9, K7TTT8, C0P8S9, B4FX58, Q8W149, C0PMQ0, B6SY05, B4FQ73, K7VZN2, B6T2W8, K7VKP3, P11143, M1H548, K7V1I2, B8A134, B4FK28, B8A305, C4J0D7, B4FX58, B6SGQ1, M1H548 K7V1I2 B4FKD1 C0PL59 B4FX58 B6T7C2 K7V0H) belonged to the three pathways, of which 26 proteins except K7VKP3 were regulated by ABA under osmotic stress. This was consistent with the signaling pathways related to protein synthesis, such as the biosynthesis of amino acids and ribosome, which had the second greatest number of proteins (B8A367, C0HHU2, B6TS38, C0HIV2, B4FRM3, B4FWX5, C0PKN2, B6TPG2, B4FCE7, O04014, B4FCK4, B4FWI0), and only B4FWI0 was not regulated by ABA under osmotic stress. Moreover, the signaling pathways related to photosynthesis, such as carbon fixation in photosynthetic organisms and photosynthesis, had the third greatest number of proteins (B7ZYP6, B6TS38, B4FRM3, B4FZ38, P04711, B4FQ59, C0PNN7, P24993, B4FAW3, B6T9S5, P05022), and all were regulated by ABA under osmotic stress. These results indicated that the three pathways related to mRNA synthesis, protein synthesis and photosynthesis played a vital role in ABA enhancing maize endurance to osmotic stress.

## Discussion

ABA governs many aspects of plant physiology, and the induced reversible phosphorylation of proteins is an important regulator of ABA signaling^23^. The degree of specificity and redundancy among these factors is hotly debated. Previously, there had been no comprehensive survey of phosphorylation sites regulated by ABA in maize exposed to osmotic stress. We have performed a comparative, global analysis of ABA effects on maize protein phosphorylation under osmotic stress using the ABA mutant *vp5* and wild-type *Vp5* and identified known associations with ABA pathways and proteins that contain strongly induced phosphorylation sites.

### ABA regulation of phosphorylation at transcriptional and post-translational levels

The interaction between specific transcription factors and their cis-elements causes the expression of stress inducible genes. Abiotic stress regulation also occurs at post-transcriptional and post-translational levels. The former involves pre-mRNA processing, which starts with intron splicing and exon joining^24^. In Arabidopsis, the phosphorylation state of the ABA-responsive element binding protein 3, the bZIP family transcription factor, GsZFP1, an ABA-responsive C2H2-type zinc finger protein, and the Topless transcription repressor was regulated by exogenous ABA treatment^9^,^25^. TFs which involved in ABA-mediated gene expression are increasingly recognized as promising candidates to create useful transgenic crops that can tolerate drought stress^26^. Our data showed that many ABA-regulated phosphoproteins were involved in a series of DNA/RNA-related processes and protein syntheses/degradation under osmotic stress (Table 2, Tables S1 and S2). ABA triggered the phosphorylation or dephosphorylation of 17 zinc finger protein transcription factors and other transcription factors, such as the gata transcription factor (B6TFI9), and 6 ribosomal proteins under osmotic stress. These results imply that phosphoproteins participating in gene transcription and translation may be major targets for regulatory phosphorylation during osmotic stress and that ABA-mediated transcriptional regulation plays a crucial role in many cellular processes of plants response to stress.

### Ubiquitination and transporter–mediated ABA signaling under osmotic stress

Ubiquitination is a major modifier of signaling in all eukaryotes that causes the conjugation of ubiquitin to the lysine residues of acceptor proteins. The targeted protein is then subjected to degradation by the 26S proteasome, which is the major protein degradation system in eukaryotes and greatly influences plant growth and development by modulating the activity, localization, and stability of proteins under stress^19^. Many signaling details of ABA responses to abiotic stresses, such as salt and dehydration stress have been well elucidated in large studies using ABA mutants^27^,^28^. In salt and/or drought stress signaling, many E3 ligases mediate the stress response in ABA-dependent and ABA-independent pathways^19^. In this study, by using the maize ABA-deficient mutant *vp5* and wild-type *Vp5*, the phosphorylation level of 8 phosphoproteins related to the ubiquitin/26S proteasome system was regulated by osmotic stress in an ABA-dependent way. These results indicate that the changes in expression abundance or modification state of the ubiquitin/26S proteasome complex protein subunits directly reflected the related-protein degradation, or not, during some biological processes and was necessary for many processes involved in plant responses to abiotic stresses.

Plasma membrane intrinsic proteins have been shown to be primary channels mediating water uptake in plant cells and their regulation via phosphorylation events^29^. In Arabidopsis, the phosphorylation level of plasma membrane intrinsic protein 2-A/B (PIP2-A/B), intrinsic protein 3, intrinsic protein 2–8 and intrinsic protein 2–4 was found to significantly decrease after ABA treatment up to 30 min^7^. Na^+^/H^+^ exchangers in the plasma membrane or vacuole have been recognized as one of the key regulatory mechanisms mediating cellular signaling by maintaining ion homeostasis. Previous studies indicated Na^+^/H^+^ exchangers can be up-regulated by salt, drought and heat stress^30^ and ABA treatment^31^. In the present study, the phosphorylation level of two aquaporins, one mitochondrial import inner membrane translocase subunit tim14 and one Na^+^/H^+^ antiporter involved in the signaling of ABA- regulated maize response to osmotic stress. Moreover, the phosphorylation states of other important transporters, such as probable sugar phosphate/phosphate translocator at3g17430-like (B6U937), vacuolar amino acid transporter 1-like (C0PEW7), hexose transporter (B6U6U2), zinc transporter (K7U2V8), solute carrier family facilitated glucose transporter member 8 (K7UMX4), abc transporter b family member 1-like (Q6UNK5), Na^+^/H^+^ antiporter (B4FZY1), K^+^ efflux antiporter 5-like (B6SP24) and sodium hydrogen exchanger 6-like (B4FS09) were regulated by ABA under osmotic stress. In summary, these results show that the phosphorylation and dephosphorylation of transporters might help the cell to maintain solute and ion stability, which might play an active role in ABA-regulated plant adaptation to osmotic stress.

### Phosphorylation states of chloroplast proteins regulated by osmotic stress in an ABA-dependent way

Photosynthesis is a key process affected by environmental stress. The expression patterns of most photosynthesis-related proteins are complex under drought stress^32^. ABA signal transduction has been extensively studied, and numerous signaling components have been identified, including the chloroplast envelope-localized ABA receptor^33^, which provides stronger evidence that ABA plays an active role in regulating chloroplast response to stress. Previous reports have shown that PLASTID MOVEMENT IMPAIRED1 involved in blue-light-induced chloroplast movement, functions in ABA-response pathways and participates in the regulation of ABA accumulation during periods of water deficit at the seedling stage^34^. Other reports have also shown that some chloroplast proteins, such as the light-harvesting chlorophyll a/b binding proteins, ATP synthase, 2-cys peroxiredoxin BAS1, elongation factor 1a, phosphoglycerate kinase, protochlorophyllide reductase A, rubisco large chain, fructokinase-2, β-glucosidase, glyceraldehyde-3-phosphate dehydrogenase A, and phosphoribulokinase are involved in ABA signal transduction and play a positive role in maize response to ABA and drought stress^32^. In the present study, the phosphorylation level of 21 chloroplast proteins displayed significant differences between *Vp*5 and *vp*5 under osmotic stress. However, taking into account the fact that phosphorylation changes of chloroplasts proteins in white leaves of *vp*5 might be due to a carotenoid side-effect rather than a direct effect of ABA, so we supposed that the phosphorylation of the chloroplast proteins might by regulated by osmotic stress in an ABA-dependent or –independent way.

### Phosphorylated protein kinases and phosphatases that are associated with signal perception and transduction

Protein phosphorylation, which plays a key role in most cellular activities, is a reversible process mediated by protein kinase and phosphatases. The interplay between phosphatases and kinases strictly controls biological processes, such as metabolism, transcription, cell cycle progression, differentiation, cytoskeletal arrangement and cell movement, apoptosis, intercellular communication, and immunological functions^35^,^36^. Recent studies have established a simple ABA signaling model consisting of three core components: PYR/PYL/RCAR receptors, 2C-type protein phosphatases, and SnRK2 protein kinases. This model highlights the importance of protein phosphorylation mediated by SnRK2. Other protein kinases, e.g., Ca^2+^ dependent protein kinase (CDPK) and mitogen-activated protein kinase (MAPK), have been identified as ABA signaling factors^37^,^38^. In fact, Arabidopsis *snrk2.2/2.3/2.6* triple-mutant plants are nearly completely insensitive to ABA; most of the phosphoproteins regulated by ABA are triggered by SnRK2s-mediated phosphorylation. These proteins are involved in flowering time regulation, RNA and DNA binding, miRNA and epigenetic regulation, signal transduction, chloroplast function, and many other cellular processes^39^. Moreover, in maize, research results show that ZmPYL3 and ZmPP2C16 proteins are the most likely members of the receptors and the second components of the ABA signaling pathway, respectively^4^. In this study, 33 kinases and 1 phosphatase were identified under osmotic stress (Table 5). The phosphorylation level of CDPK-related protein kinase (B6SYP7), SNRK SAPK family protein kinase (B7ZXP0), and map kinase family protein isoform 1 (B8A0M9) was up-regulated by osmotic in an ABA-dependent way. Thus, our results did not only prove this model but also highlighted the importance of protein phosphorylation that is mediated by these kinases in maize responses to osmotic stress and ABA signaling.

Overall, protein phosphorylation/dephosphorylation is a central PTM in plant hormone signaling, which usually results in a functional change of the target protein by changing enzyme activity, cellular location, or association with other proteins. In this study, we have identified up to 3,484 unique phosphopeptides, corresponding to 2,863 phosphoproteins using Multiplex run iTRAQ-based quantitative proteomic and LCMS/MS methods. Differential phosphorylation and expression patterns of individual protein isoforms were detected in maize response to osmotic stress and ABA. Our results provide a comprehensive dataset of phosphopeptides and phosphorylation sites regulated by ABA in maize adaptation to osmotic stress. This will be helpful to elucidate the ABA-mediate mechanism of maize endurance to drought by triggering phosphorylation or dephosphorylation cascades.

## Methods

### Plant material and treatments

Maize mutant *vp5* and wild-type *Vp5* seedlings were used in this study. The *vp5* mutant is deficient in ABA biosynthesis and has decreased amounts of ABA^16^. Homozygous recessive kernels (*vp5*/*vp5*) lack carotenoids, resulting in white endosperm and embryos, which is easily distinguishable from the yellow, wild type kernels (*Vp5*/-). Because the recessive mutation is lethal in the homozygous state, it is maintained as a heterozygote. Seeds of *vp5* and *Vp5* plants were obtained by selfing plants grown from heterozygous seeds (Maize Genetics Stock Center, Urbana, IL, USA).

*Vp5* and *vp5* seeds were germinated on moistened filter paper after being surface-sterilized for 10 min in 2% hypochlorite and then rinsed in distilled water. After germination for 2 d, both *vp5* and *Vp5* seedlings were cultured in Hogland’s nutrient solution in a light chamber (day 28 °C/night 22 °C, relative humidity 75%) under 400 μmol m^−2^ s^−1^ photosynthetically active radiations with a 14/10 h (day/night) cycle. After 2 weeks, the seedlings were subjected to osmotic stress by placing them in a −0.7 MPa PEG6000 solution for 8 h at 28 °C under relative humidity 40%. Control seedlings were maintained at 28 °C under relative humidity 75%. Subsequently, leaves of treated and untreated seedlings were sampled, immediately frozen in liquid N_2_ and stored at −80 °C until analysis. Three or five replicates were performed for each treatment.

### Protein Extraction

Total proteins from the second new expand leaf of the maize seedlings were extracted according to the following procedure. Approximately 0.5 g of fresh leaves from each biological replicate were ground into a fine power in liquid N_2_ in a mortal and further ground in a 4 ml SDS buffer (30% sucrose, 2% SDS, 100 mM Tris-HCl, pH 8.0, 50 mM EDTA-Na_2_, 20 mM DTT) and 4 ml phenol (Tris-buffered, pH 8.0) in a 10 ml tube, followed by the addition of 1 mM phenylmethanesulfonyl fluoride (PMSF) and PhosSTOP Phosphatase Inhibitor Cocktail (one tablet/10 ml; Roche, Basel, Switzerland) to inhibit protease and phosphatase activity. The mixture was thoroughly vortexed for 30 s and the phenol phase was separated by centrifugation at 14,000 × g and 4 °C for 15 min. The upper phenol phase was pipetted into fresh 10 mL tubes and four fold volumes of cold methanol plus 100 mM ammonium acetate were added. After centrifugation at 14,000 × g and 4 °C for 15 min, the supernatant was carefully discarded and the precipitated proteins were washed twice with cold acetone. Finally, the protein mixtures were harvested by centrifugation. Using a 2-D Quant Kit (Amersham Bioscience, America) containing bovine serum albumin (BSA) (2 mg/mL) as the standard, we carried out the measurement of protein content. To enhance the quantitative accuracy, extracted proteins from every biological replicate were adjusted to the same concentration for the subsequent analysis^7^,^39^.

### Protein digestion and iTRAQ labeling

Protein digestion was performed according to the FASP procedure^40^,^38^, and the resulting peptide mixture was labeled using the 4-plex iTRAQ reagent according to the manufacturer’s instructions (Applied Biosystems). Briefly, 200 μg of proteins for each sample were incorporated into 30 μl of STD buffer (4% SDS, 100 mM DTT, 150 mM Tris-HCl pH 8.0). The detergent DTT and other low-molecular-weight components were removed using UA buffer (8 M Urea, 150 mM Tris-HCl pH 8.0) by repeated ultrafiltration (Microcon units, 30 kD). Then, 100 μl of 0.05 M iodoacetamide in UA buffer was added to block reduced cysteine residues, and the samples were incubated for 20 min in darkness. The filters were washed with 100 μl of UA buffer three times and then washed twice with 100 μl of DS buffer (50 mM trimethylammonium bicarbonate at pH 8.5). Finally, the protein suspensions were digested with 2 μg of trypsin (Promega) in 40 μl of DS buffer overnight at 37 °C, and the resulting peptides were collected as a filtrate. The peptide content was estimated by UV light spectral density at 280 nm using an extinction coefficient of 1.1 of 0.1% solution that was calculated on the basis of the frequency of tryptophan and tyrosine in vertebrate proteins.

For labeling, each iTRAQ reagent was dissolved in 70 μl of ethanol and added to the respective peptide mixture. The samples, *Vp5*-control, *Vp5*-OS (osmotic stress), *vp5*-control, and *vp5*-OS, were multiplexed and vacuum dried. Three independent biological experiments were performed.

### Peptide fractionation with strong cation exchange (SCX) chromatography for proteomic analysis

iTRAQ labeled peptides were fractionated by SCX chromatography using the AKTA Purifier system (GE Healthcare). The dried peptide mixture was reconstituted and acidified with 2 ml buffer A (10 mM KH_2_PO_4_ in 25% of ACN, pH 2.7) and loaded onto a PolySULFOETHYL 4.6 × 100 mm column (5 μm, 200 Å, PolyLC Inc, Maryland, USA.). The peptides were eluted at a flow rate of 1 ml/min with a gradient of 0–10% buffer B (500 mM KCl, 10 mM KH_2_PO_4_ in 25% of ACN, pH 2.7) for 2 min, 10–20% buffer B for 25 min, 20–45% buffer B for 5 min, and 50–100% buffer B for 5 min. The elution was monitored by absorbance at 214 nm, and fractions were collected every 1 min. The collected fractions (about 30 fractions) were finally combined into 10 pools and desalted on C18 Cartridges (Empore™ SPE Cartridges C18 (standard density), bed I.D. 7 mm, volume 3 ml, Sigma). Each fraction was concentrated by vacuum centrifugation and reconstituted in 40 μl of 0.1% (v/v) trifluoroacetic acid. All samples were stored at −80 °C until LC-MS/MS analysis.

### Liquid chromatography (LC)—electrospray ionization (ESI) tandem MS (MS/MS) analysis by Q Exactive for proteomic analysis

Experiments were performed on a Q Exactive mass spectrometer that was coupled to Easy nLC (Proxeon Biosystems, now Thermo Fisher Scientific). 10 μl of each fraction was injected for nanoLC-MS/MS analysis. The peptide mixture (5 μg) was loaded onto a the C18-reversed phase column (Thermo Scientific Easy Column, 10 cm long, 75 μm inner diameter, 3μm resin) in buffer A (0.1% Formic acid) and separated with a linear gradient of buffer B (80% acetonitrile and 0.1% Formic acid) at a flow rate of 250 nl/min controlled by IntelliFlow technology over 140 min. MS data was acquired using a data-dependent top10 method dynamically choosing the most abundant precursor ions from the survey scan (300–1800 m/z) for HCD fragmentation. Determination of the target value is based on predictive automatic gain control (pAGC). Dynamic exclusion duration was 60 s. Survey scans were acquired at a resolution of 70,000 at m/z 200 and resolution for HCD spectra was set to 17,500 at m/z 200. Normalized collision energy was 30 eV and the underfill ratio, which specifies the minimum percentage of the target value likely to be reached at maximum fill time, was defined as 0.1%. The instrument was run with peptide recognition mode enabled.

### Phosphopeptide enrichment by TiO_2_ beads

The labeled peptides were mixed, concentrated by a vacuum concentrator and resuspended in 500 μL of loading buffer (2% glutamic acid/65% ACN/ 2% TFA). Then, TiO_2_ beads were added and then agitated for 40 min. The centrifugation was performed for 1 min at 5000 g, resulting in the first beads. The supernatant from the first centrifugation was mixed with additional TiO_2_ beads, resulting in the second beads that were collected as before. Both bead groups were combined and washed three times with 50 μL of washing buffer I (30% ACN/3%TFA) and then washed three times with 50 μL of washing buffer II (80% ACN/0.3% TFA) to remove the remaining non-adsorbed material. Finally, the phosphopeptides were eluted with 50 μL of elution buffer (40% ACN/15% NH_4_OH)^41^, followed by lyophilization and MS analysis.

### MS/MS for phosphoproteomics analysis

Five μl of the phosphopeptide solution mixed with 15 μl of 0.1% (v/v) trifluoroacetic acid and then 10 μl of the solution mixture was injected into a Q Exactive MS (Thermo Scientific) equipped with Easy nLC (Proxeon Biosystems, now Thermo Scientific) for nanoLC-MS/MS analysis. The peptide mixture was loaded onto a C18-reversed phase column (15 cm long, 75 μm inner diameter, RP-C18 3 μm, packed in-house) in buffer A (0.1% Formic acid) and separated with a linear gradient of buffer B (80% acetonitrile and 0.1% Formic acid) at a flow rate of 250 nL/min controlled by IntelliFlow technology over 240 min. The peptides were eluted with a gradient of 0%–60% buffer B from 0 min to 200 min, 60% to 100% buffer B from 200 min to 216 min, 100% buffer B from 216 min to 240 min.

For MS analysis, peptides were analyzed in positive ion mode. MS spectra were acquired using a data-dependent top10 method dynamically choosing the most abundant precursor ions from the survey scan (300–1800 m/z) for HCD fragmentation. Determination of the target value is based on predictive Automatic Gain Control (pAGC). Dynamic exclusion duration was 40 s. Survey scans were acquired at a resolution of 70,000 at m/z 200, and the resolution for the HCD spectra was set to 17,500 at m/z 200. Normalized collision energy was 27 eV, and the under fill ratio, which specifies the minimum percentage of the target value likely to be reached at maximum fill time, was defined as 0.1%. The instrument was run with peptide recognition mode enabled.

### Data analysis

MS/MS spectra were searched using Mascot 2.2 (Matrix Science) embedded in Proteome Discoverer 1.4 against the uniprot_Zea_mays_87227_20150504.fasta (87227 sequences, download May 4th, 2015) and the decoy database. The parameters used in Mascot searches for normal peptides were as follows: Peptide mass tolerance: 20 ppm, MS/MS tolerance: 0.1 Da, Enzyme: Trypsin, max missed cleavage: 2, Fixed modification: Carbamidomethyl (C), iTRAQ4plex(K), iTRAQ4plex(N-term), Variable modification: Oxidation (M), FDR ≤0.01. The protein and peptide probabilities were set at 50 and 60%, respectively. Only proteins with at least two unique peptides with a Mascot score of at least 25 and detected in at least two replicates were further used. For peptides after phosphopeptide enrichment, the following options were used. Peptide mass tolerance: 20 ppm, MS/MS tolerance: 0.1 Da, enzyme: trypsin, max missed cleavage: 2, fixed modification: Carbamidomethyl (C), iTRAQ4plex (K), iTRAQ4plex (N-term), variable modification: Oxidation (M), phosphorylation (S/T/Y). The score threshold for peptide identification was set at a 5% or 1% false discovery rate (FDR), and the PhosphoRS site probabilities estimate the probability (0–100%) of each phosphorylation site. The PhosphoRS site probabilities above 75 percent indicate that a site is truly phosphorylated^42^.

For each replicate of both proteomics and phosphoproteomics, iTRAQ ratios between osmotic stress (OS) and controls for each run were converted to z-scores to normalize the data.

### Bioinformatics

The molecular functions of the identified proteins were classified according to their gene ontology annotations and their biological functions. The subcellular localization of the unique proteins identified in this study was predicted using the publicly available program WolfPsort ( http://wolfpsort.org). Protein-protein interaction networks were analyzed using the publicly available program STRING ( http://string-db.org/). STRING is a database of known and predicted protein-protein interactions. The interactions include direct (physical) and indirect (functional) associations, and they are derived from four sources: the genomic context, high-throughput experiments, coexpression and previous knowledge. STRING quantitatively integrates the interaction data from these sources for a large number of organisms and, where applicable, transfers information between these organisms.

Motif-X online software ( http://motif-x.med.harvard.edu/motif-x.html) was used to find phosphorylation site motifs in the identified maize proteins and to predict the specificity of these motifs based on the identified phosphopeptide sequences. The parameters were set to peptide length = 21, occurrence = 5, and statistical significance for p-values of less than 0.000001.

### NABA assay

Maize leaves (0.5–1.0 g) were ground in liquid N_2_ with a mortar, extracted with 2 ml of ice-cold 80% methanol containing 1 mM butylated hydroxytoluene to prevent oxidation, and then stored overnight at 4 °C. The extracts were centrifuged at 12000 g for 15 min at 4 °C. The pellets were extracted once and stored at 4 °C for 1 h. The two resulting supernatants were combined and passed through a C18 Sep-Pak cartridge (Waters, Milford, MA, USA). The efflux was collected and dried in N_2_. The residues were then dissolved in 10 mM phosphate buffer solution (pH 7.4) and concentrations of ABA were determined in enzyme-linked immunosorbent assay (ELISA)^32^. Statistical analyses of the physiological measurements were conducted using independent Student’s t-tests with SPSS statistics software (version 17.0).

### Statistical analysis

The phosphoproteins, phosphopeptides and ABA assays were the mean of three replicates. The means were compared by a one-way analysis of variance and Duncan’s multiple range test at a 5% level of significance. FDR attained by Benjamini-Hochberg method were used to adjust p-values (correction for multiple comparisons). The significance of difference between *Vp*5 and *vp*5 were compared by T-Test analysis at a 5% level.

## Additional Information

**How to cite this article**: Hu, X. *et al.* Quantitative iTRAQ-based proteomic analysis of phosphoproteins and ABA-regulated phosphoproteins in maize leaves under osmotic stress. *Sci. Rep.* **5**, 15626; doi: 10.1038/srep15626 (2015).

## Supplementary Material

Supplementary Information

## Figures and Tables

**Table 1:** Proteins with more than 1.5-folds phosphorylation level change in two maize genotypes response to ABA and osmotic stress.

UniProt ID	>Protein name	>Sequence of phosphorylati-on peptides	>PhosphoRS-Site Probabilities (>75%)	>Ion score	*Vp5*: OS/control			*vp5*: OS/control			*Vp5*: OS/control		*vp5*: OS/control		>T-test	>Regulation of ABA and osmotic stress for peptides phosphosites
					1	2	3	1	2	3	Average^a^	P-Value/FDR	Average^a^	P-Value/FDR		
B6TE49	probable receptor-like protein kinase at1g33260-like	gGFsTVYLAsLSSSR	S(4):77.9	18	2.151	2.481	3.261	0.299	0.343	0.301	2.631	0.000/0.000	0.314	0.000/0.000	0.020	Up-regulated by osmotic stress with ABA-dependent way
K7V8B2	tata-binding protein-associated factor 172-like	sSAGtTPSk	S(1):100.0; T(5):99.9	15	4.345	3.219	2.340	0.455	0.221	0.599	3.301	0.000/0.000	0.425	0.000/0.000	0.044	Up-regulated by osmotic stress with ABA-dependent way
K7TWA4	regulatory-associated protein of tor 1-like	fRtPPVsPPQHDFLPGLR	T(3):100.0; S(7):100.0	13	0.232	0.362	0.490	0.232	0.622	0.455	0.361	0.000/0.000	0.436	0.000/0.000	0.505	Down-regulated by osmotic stress with ABA-independent way
*C0PLA9	nodulin-like protein	eEVTEDSENASSSTtALGGsNQDLSSGk	S(20):99.9	43	1.813	1.592	1.203	0.403	0.556	0.469	1.536	0.041/0.057	0.476	0.000/0.000	0.032	Up-regulated by osmotic stress with ABA-dependent way
*B4FBC9	patellin family protein	aAEADsEEEk	S(6):100.0	44	2.015	2.745	2.489	0.505	0.477	0.375	2.416	0.000/0.002	0.452	0.000/0.000	0.014	Up-regulated by osmotic stress with ABA-dependent way
K7U7E1	brefeldin a-inhibited guanine nucleotide-exchange protein 1-like	vLENVHQPsFLQk	S(9):100.0	17	0.409	0.378	0.312	0.789	0.400	0.543	0.366	0.000/0.000	0.577	0.000/0.001	0.179	Down-regulated by osmotic stress with ABA-independent way
*K7TWZ6	clustered mitochondria isoform x1	qcDVLsPEEYsDEGWQAASmR	S(6):99.6	17	2.267	2.092	2.823	0.561	0.443	0.650	2.394	0.000/0.001	0.551	0.000/0.001	0.008	Up-regulated by osmotic stress with ABA-dependent way
*K7V8M7	mdr-like abc transporter	qIsINk	S(3):100.0	21	0.578	0.628	0.642	0.571	0.555	0.604	0.616	0.010/0.021	0.577	0.000/0.000	0.175	Down-regulated by osmotic stress with ABA-independent way
*B8A0C6	phosphatidate phosphatase lpin2-like	eLVPGGEDsGtGSDDEtVNEPEPPAR	S(9):75.0; T(11):75.0; S(13):75.0; T(17):75.0	21	2.012	1.812	2.166	0.676	0.488	0.595	1.997	0.001/0.005	0.586	0.000/0.001	0.003	Up-regulated by osmotic stress with ABA-dependent way
*B4FWX5	dihydroxy-acid mitochondrial-like	nAMVIVmALGGstNAVLHLIAIAR	S(12):100.0; T(13):100.0	16	1.803	1.503	2.071	0.602	0.613	0.593	1.792	0.008/0.017	0.603	0.000/0.001	0.020	Up-regulated by osmotic stress with ABA-dependent way
*B4FS10	TPA: hypothetical protein ZEAMMB73_767959	aAGGDDSGsGGGFNLGGLGGLFAk	S(7):50.0; S(9):50.0	25	0.538	0.617	0.476	0.605	0.922	0.409	0.544	0.004/0.011	0.646	0.000/0.001	0.449	Down-regulated by osmotic stress with ABA-independent way
*B8A0C6	phosphatidate phosphatase lpin2-like	eLVPGGEDSGtGSDDETVNEPEPPAR	T(11):75.0	45	1.567	1.632	2.166	0.612	0.656	0.595	1.788	0.048/0.060	0.621	0.001/0.004	0.029	Up-regulated by osmotic stress with ABA-dependent way
*K7V792	splicing factor 3b subunit 1-like isoform x1	mADADAtPAAGGAtPGATPSGAWDAtPk	T(7):99.7; T(26):100.0	26	1.786	1.565	2.019	0.666	0.612	0.671	1.790	0.000/0.001	0.650	0.003/0.011	0.010	Up-regulated by osmotic stress with ABA-dependent way
*B6U1M6	transposon protein	lALPLAGGHVtDNDGEGTAERPTk	T(11):93.7	11	0.513	0.491	0.578	1.612	1.499	1.480	0.527	0.005/0.011	1.530	0.005/0.017	0.003	Up-regulated by osmotic stress, but down-regulated by ABA
*B6U0Y9	atp binding protein	aVQVSPILDGNQtDADSNTAGEEVASR	T(13):96.4	52	1.512	1.492	1.557	1.647	1.590	1.558	1.520	0.004/0.011	1.598	0.000/0.001	0.190	Up-regulated by osmotic stress with ABA-independent way
*B6SP06	glycine-rich protein 2b	sLNDGDAVEYTVGsGNDGR	S(14):99.9	41	2.360	1.781	2.014	1.943	1.484	1.437	2.052	0.002/0.007	1.621	0.005/0.017	0.034	Up-regulated by osmotic stress with ABA-dependent and independent way
*B6TB18	lipid phosphate phosphatase 3	eTLNDVESGsAR	S(10):100.0	69	0.175	0.143	0.307	1.570	1.439	1.234	0.208	0.000/0.000	1.415	0.005/0.016	0.014	Up-regulated by osmotic stress, but down-regulated by ABA
K7U2M6	heat shock protein sti	dVEPEPEAEPmDLtDEEkDR	T(14):100.0	11	0.353	0.212	0.495	1.523	1.533	1.484	0.353	0.005/0.012	1.513	0.005/0.016	0.007	Up-regulated by osmotic stress, but down-regulated by ABA
K7USN0	2og-fe oxygenase family protein	aPVMMVAAAPARPmVmASSGTGGGNIsk	S(27):75.0	15	1.857	1.788	2.201	1.487	1.568	1.513	1.949	0.005/0.011	1.523	0.005/0.016	0.091	Up-regulated by osmotic stress with ABA-independent way
*B4FKD1	nucleoporin nup53-like	eGSPmDGVVQyQQQSPTTPSGQQSQQQk	Y(11):75.0	14	0.512	0.508	0.581	1.523	1.545	1.488	0.534	0.004/0.011	1.519	0.004/0.016	0.002	Up-regulated by osmotic stress, but down-regulated by ABA
B6TCM5	duf1664 domain family protein isoform 1	hNMANAVssMTkHLEQVQssLAAAk	S(19):99.6; S(20):99.6	26	3.516	3.110	3.123	1.539	1.496	1.700	3.250	0.004/0.010	1.578	0.004/0.014	0.009	Up-regulated by osmotic stress with ABA-dependent and independent way
Q3MQ01	autophagy protein 5	sQEAEQALAsPAEAGFAk	S(10):81.9	11	1.861	1.512	1.631	1.651	1.499	1.508	1.668	0.029/0.043	1.553	0.003/0.011	0.180	Up-regulated by osmotic stress with ABA-independent way
*K7UKU6	protein decapping 5-like	iGQLNDEPNGYEDDVIEDDEIsPR	S(22):100.0	54	2.432	2.821	2.066	1.523	1.613	1.528	2.440	0.000/0.001	1.555	0.002/0.009	0.045	Up-regulated by osmotic stress with ABA-dependent and independent way
*P04711	phosphoenolpyruvate carboxylase	hHsIDAQLR	S(3):100.0	35	5.006	3.801	4.766	1.974	1.447	1.377	4.524	0.000/0.000	1.599	0.001/0.006	0.011	Up-regulated by osmotic stress with ABA-dependent and independent way
*O04014	tpa:40s ribosomal protein s6	dRRsEsLAk	S(4):100.0; S(6):100.0	14	10.27	2.875	2.829	1.467	1.641	1.828	5.324	0.000/0.000	1.645	0.001/0.005	0.288	Up-regulated by osmotic stress with ABA-dependent and independent way
C0P2E1	disease resistance protein rga2-like	aHFPVImLYSFtsTyDVk	Y(15):94.0	16	3.023	3.640	4.087	1.533	1.831	1.557	3.583	0.000/0.000	1.640	0.001/0.005	0.024	Up-regulated by osmotic stress with ABA-dependent and independent way
B7ZYP2	pentatricopeptide repeat-containing protein *at4g22760-like*	aGDIPAARAmFEAmPARDVVsWNSMVAGLAk	S(21):80.0	14	0.389	0.455	0.299	1.678	1.723	1.523	0.381	0.000/0.000	1.641	0.001/0.004	0.000	Down-regulated by osmotic stress with ABA-dependent way
K7U3J5	set domain protein sdg117	dDTIVcsPVDLSDAcQSGmDR	S(7):92.7	12	3.544	2.809	3.211	1.812	1.578	1.593	3.188	0.000/0.000	1.661	0.001/0.003	0.010	Up-regulated by osmotic stress with ABA-dependent and independent way
*K7UKZ7	transcription elongation factor spt6-like	eScPtLLSFDSDEDNEDIESDAR	T(5):79.0	17	0.623	0.647	0.712	1.701	1.631	1.669	0.661	0.002/0.007	1.667	0.000/0.003	0.001	Down-regulated by osmotic stress with ABA-dependent way
*B4FXH0	act-domain containing protein kinase family protein	iEDMDSAyDsDASEEGDDDGDDLSVR	Y(8):84.9	18	0.347	0.251	0.196	1.701	1.723	1.651	0.265	0.000/0.000	1.692	0.000/0.002	0.001	Down-regulated by osmotic stress with ABA-dependent way
*K7TW55	translocase of chloroplast chloroplastic-like	gGNLGPTEAEAETDDGGEEPASGDGEtPASLAAPMPVVESk	T(27):83.3	63	1.822	1.529	1.532	2.120	1.593	1.681	1.628	0.004/0.011	1.798	0.000/0.001	0.130	Up-regulated by osmotic stress with ABA-independent way
M1H548	arginine serine-rich protein 45-like	rsPsPPPRR	S(2):100.0; S(4):100.0	10	0.345	0.234	0.123	2.072	1.503	1.638	0.234	0.000/0.000	1.738	0.000/0.001	0.008	Down-regulated by osmotic stress with ABA-dependent way
*K7V1A7	chromatin structure-remodeling complex protein syd-like isoform x4	aAVVAELFGDATEGGSDQPLPsPR	S(22):94.8	26	2.084	1.824	1.508	1.562	1.624	2.127	1.805	0.011/0.022	1.771	0.000/0.001	0.929	Up-regulated by osmotic stress with ABA-independent way
*B6UEN7	ubiquitin ligase protein cop1	aAsAsPQGPAEEGEGPADR	S(3):100.0; S(5):100.0	28	2.492	3.032	2.412	1.704	1.600	1.801	2.645	0.000/0.000	1.702	0.000/0.001	0.063	Up-regulated by osmotic stress with ABA-dependent and independent way
*O04014	tpa: 40s ribosomal protein s6	sEsLAk	S(1):100.0; S(3):100.0	20	4.269	3.875	4.829	1.867	1.671	1.828	4.324	0.000/0.000	1.789	0.000/0.000	0.009	Up-regulated by osmotic stress with ABA-dependent and independent way
*Q8W149	cell division cycle 5-like	eSQtPLLGGDNPELHPSDFSGVtPR	T(4):99.9; T(23):99.9	47	1.578	2.420	1.953	2.199	1.503	1.763	1.984	0.003/0.009	1.822	0.000/0.000	0.750	Up-regulated by osmotic stress with ABA-independent way
*B8A298	histone-lysine n- h3 lysine-9 specific suvh1-like	dSESsPQPPIAAPAESGk	S(5):95.5	13	3.661	2.500	3.604	3.044	1.528	1.790	3.255	0.000/0.000	2.120	0.000/0.000	0.086	Up-regulated by osmotic stress with ABA-dependent and independent way
C0P2E1	disease resistance protein rga2-like	aHFPVImLYsFtstYDVk	S(10):78.0; T(12):78.0; S(13):78.0; T(14):78.0	17	0.653	0.281	0.483	2.101	2.105	1.976	0.472	0.001/0.003	2.061	0.000/0.000	0.006	Down-regulated by osmotic stress with ABA-dependent way
*B6TXK5	uncharacterized protein LOC100277637	gPHAstDDEEEEDDDDEDAYEVER	S(5):99.9; T(6):99.9	18	1.547	1.588	1.778	2.020	2.122	2.501	1.638	0.003/0.009	2.214	0.000/0.000	0.017	Up-regulated by osmotic stress, but down-regulated by ABA
*O04014	tpa: 40s ribosomal protein s6	skLsAAAk	S(1):100.0; S(4):100.0	16	9.494	10.39	11.99	2.572	2.373	2.786	10.63	0.000/0.000	2.577	0.000/0.000	0.007	Up-regulated by osmotic stress with ABA-dependent and independent way
*P24993	photosystem ii phosphoprotein	atQtVEDSSRPkPk	T(2):100.0; T(4):100.0	15	2.148	1.578	1.557	4.661	2.294	2.004	1.761	0.002/0.006	2.987	0.000/0.000	0.199	Up-regulated by osmotic stress, but down-regulated by ABA

^a^Each value represents the average of three biological replicas. The average is significant at a p < 0.05 and a false discovery rate (FDR) <0.05 level. Moreover, these peptides whose UniProt ID are signed with * are also significant under FDR <0.01. FDR values attained by Benjamini-Hochberg method were shown in column and were used to adjust p-values (correction for multiple comparisons). These phosphopeptides whose FDR values were signed with delete line ‘—’ were not significant. ‘-’, not measured. T-test is used to identify whether the difference is significant. A T-test value <0.05 is considered to be significant. OS = osmotic stress.

**Table 2:** Most abundance phosphoproteins mediated ABA signaling pathways under osmotic stress.

>Protein/peptide sequence	>UniProt ID	>Protein name	>PhosphoRS-Site Probabilities (>75%)	>Ion score	*Vp5*: OS/control			*vp5*: OS/control			*Vp5*: OS/control		*vp5*: OS/control		t-test	Regulation of ABA and osmotic stress for peptides phosphosites
					1	2	3	1	2	3	Averagea	P-Value	Averagea	P-Value		
Transporter																
aLGSFRsNA	*Q9ATM4	aquaporin pip2-7	S(7):100.0	16	0.656	0.611	0.650	0.762	0.718	0.755	0.639	0.021	0.745	0.028	0.000	Down-regulated by osmotic stress with ABA-dependent way
aLGsFR	Q9ATM4	aquaporin pip2-7	S(4):100.0	17	0.559	0.702	0.728	0.831	0.825	0.824	0.663	0.047	0.827	0.138	0.096	Down-regulated by osmotic stress with ABA-independent way
aLGsFRsNA	Q9ATM4	aquaporin pip2-7	S(4):100.0; S(7):100.0	17	0.436	0.436	0.347	0.862	0.923	0.822	0.406	0.000	0.869	0.153	0.002	Down-regulated by osmotic stress with ABA-dependent way
lGsSAsFSR	*Q9XF58	aquaporin pip2-4-like	S(3):97.3;	27	0.494	0.498	0.714	1.192	1.203	1.126	0.569	0.002	1.174	0.221	0.025	Down-regulated by osmotic stress with ABA-dependent way
xtPLIAGLAVAAtALAGR	B6T195	mitochondrial import inner membrane translocase subunit tim14	T(2):100.0; T(13):100.0	13	2.210	2.004	2.451	0.989	1.024	0.719	2.222	0.018	0.911	0.163	0.027	Up-regulated by osmotic stress with ABA-independent way
rPAsLR	B4FZY1	Na^+^/H^+^ antiporter	S(4):100.0	14	0.641	0.621	0.553	1.032	0.988	1.099	0.605	0.048	1.040	0.880	0.016	Down-regulated by osmotic stress with ABA-dependent way
gFVPFVPGsPTESsLPLLPGNEN	*B4FZY1	Na^+^/H^+^ antiporter	S(9):91.3	28	2.946	2.823	2.969	1.139	0.936	0.789	2.913	0.000	0.954	0.396	0.003	Up-regulated by osmotic stress with ABA-dependent way
gQsALGsALGLIsR	*B6U6U2	hexose transporter	S(3):100.0; S(7):100.0; S(13):100.0	75	1.683	1.692	1.634	1.071	1.048	1.095	1.670	0.026	1.071	0.580	0.003	Up-regulated by osmotic stress with ABA-dependent way
tQtGSSSNR	B6U937	probable sugar phosphate/ phosphate translocator at3g17430-like	T(1):80.0	10	6.023	4.674	3.324	0.758	0.813	0.869	4.674	0.000	0.813	0.106	0.041	Up-regulated by osmotic stress with ABA-dependent way
lSNsFLAITDsFR	C0PEW7	vacuolar amino acid transporter 1-like	S(11):94.9	29	1.653	1.553	1.653	1.041	1.141	1.241	1.620	0.041	1.141	0.357	0.019	Up-regulated by osmotic stress with ABA-dependent way
tPLGAAYEPPSAAAGGGGTtPVNIR	*C0PLZ2	probable peptide nitrate transporter at5g13400-like	T(20):95.8	28	0.522	0.526	0.580	1.392	0.823	1.129	0.543	0.043	1.115	0.400	0.075	Down-regulated by osmotic with ABA-dependent way
sAsTPR	K7U2V8	zinc transporter	S(3):97.7	15	1.733	1.930	1.833	0.945	1.113	1.045	1.832	0.007	1.034	0.760	0.000	Up-regulated by osmotic stress with ABA-dependent way
qSsLNAAGTssMAVLR	K7UMX4	solute carrier family facilitated glucose transporter member 8	S(10):97.8	55	1.728	1.821	1.691	0.912	1.121	1.054	1.747	0.018	1.029	0.701	0.005	Up-regulated by osmotic stress with ABA-dependent way
nsVSsPIMTR	Q6UNK5	abc transporter b family member 1-like	S(2):76.7	24	2.429	2.169	2.175	1.294	1.330	1.378	2.258	0.001	1.334	0.047	0.013	Up-regulated by osmotic stress with ABA-dependent way
sSEGVFVGAFLSMSStAVVskFLVEk	B6SP24	K^+^ efflux antiporter 5-like	T(16):87.3	17	—	—	—	0.454	0.436	0.424			0.438	0.000		Up-regulated by ABA
kAssLQR	B6SV26	vacuolar amino acid transporter 1-like	S(3):100.0; S(4):100.0	19	1.022	0.911	0.920	1.628	1.423	1.682	0.951	0.817	1.578	0.002	0.013	Down-regulated by ABA
sTGTAAtGGsDAGLEEGk	B6UH65	zinc transporter 2 precursor	S(1):78.4	10	0.686	0.645	0.665	2.999	3.120	2.973	0.665	0.069	3.031	0.000	0.001	Down-regulated by ABA
nYLTPFFTsQTDDDNDDDFSQQPQNR	B4FS09	sodium hydrogen exchanger 6-like	S(9):74.6	12	—	—	—	2.301	2.340	2.201			2.281	0.000		Down-regulated by ABA
eGSPMDGVVQyQQQSPTTPSGQQSQQQk	B4FKD1	nucleoporin nup53-like	Y(11):75.0	14	0.512	0.508	0.581	1.523	1.545	1.488	0.534	0.005	1.519	0.004	0.002	Up-regulated by osmotic stress, but down-regulated by ABA
tsDADsEAGSGSGGGGR	C0P5C4	abc1 family protein	S(2):95.0	16	0.701	0.774	0.787	1.660	1.594	1.612	0.754	0.197	1.622	0.001	0.003	Down-regulated by ABA
Ubiquitin-conjugating enzyme family protein-like																
ntPSmPPAVSTSSAsR	B4FHK6	ubiquitin-conjugating enzyme e2 22-like	T(2):76.1	11	0.456	0.511	0.405	0.782	0.790	0.757	0.457	0.000	0.776	0.053	0.004	Down-regulated by osmotic stress with ABA-dependent way
eVNAGIASVsR	*B4FAG8	e3ubiquitin-protein ligase rhf2a-like isoform x1	S(10):100.0	30	0.605	0.589	0.646	0.912	0.800	0.845	0.613	0.028	0.852	0.196	0.020	Down-regulated by osmotic stress with ABA-dependent way
rHsTGQstPDR	*B4FAG8	e3ubiquitin-protein ligase rhf2a-like isoform x1	S(3):97.0; T(8):97.0	28	2.211	1.890	2.017	1.401	1.201	1.303	2.039	0.002	1.302	0.057	0.002	Up-regulated by osmotic stress with ABA-dependent way
aDsPSEGLTcGSQNLPAETcPk	*K7V4D9	e3ubiquitin-protein ligase upl4-like	S(3):99.8	23	2.888	2.521	2.666	1.010	0.987	0.949	2.692	0.000	0.982	0.665	0.003	Up-regulated by osmotic stress with ABA-dependent way
sAsPSTS	C0P3H1	Ubiquitin carboxyl-terminal hydrolase isozyme l5-like	S(3):96.4;	16	0.555	0.512	0.597	0.957	0.879	1.020	0.555	0.006	0.952	0.704	0.002	Down-regulated by osmotic stress with ABA-dependent way
lGVDVNtmPAItDk	B4G0Z1	e3ubiquitin-protein ligase ubr7-like	T(12):99.6	11	0.601	0.499	0.558	1.230	1.110	1.192	0.553	0.001	1.177	0.221	0.000	Down-regulated by osmotic stress with ABA-dependent way
aAsAsPQGPAEEGEGPADR	*B6UEN7	ubiquitin ligase protein cop1	S(3):100.0; S(5):100.0	28	2.492	3.032	2.412	1.704	1.600	1.801	2.645	0.010	1.702	0.001	0.063	Up-regulated by osmotic stress with ABA-dependent and independent way
dVsNAsELATEMQYER	*K7TFK8	e3ubiquitin-protein ligase upl1-like	S(3):100.0; S(6):100.0	28	2.132	1.929	1.958	1.153	1.099	0.900	2.007	0.009	1.051	0.473	0.005	Up-regulated by osmotic stress with ABA-dependent way
lRPGQPDAVQDAStSDmEDASTSSGGQR	*K7TFK8	e3ubiquitin-protein ligase upl1-like	T(14):76.0	41	1.656	1.626	1.701	1.420	1.766	1.087	1.661	0.029	1.424	0.020	0.390	Up-regulated by osmotic stress with ABA-independent way
eNEGSSSsAGESSSmDIDk	*B6T6V5	ubiquitin carboxyl-terminal hydrolase 6-like	S(8):79.5	17	0.612	0.584	0.658	1.312	1.113	1.215	0.618	0.029	1.213	0.169	0.008	Down-regulated by osmotic stress with ABA-dependent way
sALLsYSDTVR	B6T6V5	ubiquitin carboxyl-terminal hydrolase 6-like	S(5):75.0	13	0.580	0.592	0.512	1.799	1.064	1.200	0.561	0.008	1.354	0.048	0.070	Down-regulated by osmotic stress with ABA-dependent way
Zinc finger transcription factor																
dLVVDtDDGGNANR	*B6UB08	zinc finger protein 652-a- partial	T(6):100.0	46	0.570	0.521	0.564	0.809	0.812	1.089	0.552	0.006	0.903	0.101	0.057	Down-regulated by osmotic stress with ABA-independent way
dPSINQVAsPVAAPEPVGAILPk	*B4FX96	Zinc finger ccch type domain containing protein zfn-like 1	S(9):100.0	26	0.655	0.776	0.516	1.016	0.877	0.769	0.649	0.047	0.887	0.358	0.087	Down-regulated by osmotic stress with ABA-independent way
eQGsIGITANDDPyNGNEmSPSDQR	K7UHH6	zinc finger c-x8-c-x5-c-x3-h type family protein	S(4):100	40	0.245	0.360	0.495	0.808	0.904	1.007	0.367	0.000	0.906	0.443	0.001	Down-regulated by osmotic stress with ABA-dependent way
dPAVGsSPAVsNNk	*B4FLK4	zinc finger protein 207-like isoform x1	S(6):97.3	41	0.431	0.423	0.454	1.277	1.292	1.311	0.436	0.000	1.293	0.086	0.000	Down-regulated by osmotic stress with ABA-dependent way
dWNQNFEVsPTDYLPQDSR	*B6U194	zinc finger c-x8-c-x5-c-x3-h type family protein	S(9):80.0	12	0.401	0.393	0.536	0.901	0.993	0.821	0.443	0.000	0.905	0.411	0.038	Down-regulated by osmotic stress with ABA-dependent way
lQPADsIEGTVIDRDcDEVDDAAQDSGAR	*B4FY62	tpa:c3hc zinc finger-like family protein	S(6):99.9	18	0.422	0.493	0.412	1.112	1.030	0.980	0.442	0.000	1.041	0.891	0.006	Down-regulated by osmotic stress with ABA-dependent way
dcDEVDDAAQDsGAR	*B4FY62	tpa:c3hc zinc finger-like family protein	S(12):100.0	35	2.981	1.879	2.006	1.355	1.439	1.402	2.288	0.001	1.399	0.017	0.139	Up-regulated by osmotic stress with ABA-independent way
cMVsLsPPPPk	K7UE59	ring zinc finger domain superfamily protein	S(4):100.0; S(6):100.0	21	0.498	0.556	0.587	1.332	1.376	1.385	0.547	0.002	1.364	0.020	0.000	Down-regulated by osmotic stress with ABA-dependent way
gANEEVsSINVDEDPNVPYERsPNAAIAk	*K7UBL3	zinc finger c-x8-c-x5-c-x3-h type family protein	S(7):95.1; S(22):100.0	35	0.409	0.627	0.570	0.925	1.328	1.624	0.535	0.002	1.293	0.131	0.041	Down-regulated by osmotic stress with ABA-dependent way
dSSANPPPsPGTTYGPVGSISk	*B6SW01	zinc finger ccch type domain-containing protein zfn-like 3	S(9):83.6	16	0.534	0.424	0.624	0.980	0.883	1.039	0.527	0.005	0.967	0.698	0.001	Down-regulated by osmotic stress with ABA-dependent way
lGGsDGNsEDDMDNDk	*B7ZXU2	serrate-related c2h2 zinc-finger family protein	S(4):100.0; S(8):100.0	29	1.694	1.721	1.417	1.220	1.179	1.274	1.611	0.048	1.224	0.151	0.088	Up-regulated by osmotic stress with ABA-independent way
ePGEGtSS	B6TD33	zinc finger ccch domain-containing protein 11-like	T(6):83.3	17	1.594	1.623	1.856	1.226	1.112	1.345	1.691	0.027	1.228	0.149	0.010	Up-regulated by osmotic stress with ABA-dependent way
nVDVDsDGER	*K7UZK2	zinc finger ccch domain-containing protein 44-like	S(6):100.0	26	2.282	1.968	2.223	1.365	1.063	1.211	2.158	0.001	1.213	0.192	0.001	Up-regulated by osmotic stress with ABA-dependent way
vEsSLVGSDDVLDSASDSPPsVk	C0P2B1	phd zinc finger	S(21):74.8	15	2.334	2.410	2.309	1.350	1.597	1.441	2.351	0.000	1.463	0.008	0.003	Up-regulated by osmotic stress with ABA-dependent way
nTHPPEPESIDGINDtGVQTPQQFR	*B4FZ17	dhhc-type zinc finger domain-containing protein	T(16):49.0	13	—	—	—	3.911	3.890	3.589			3.797	0.000		Down-regulated by ABA
eQGsIGItANEDPYNANEMSPSDQR	*B4FX77	zinc finger c-x8-c-x5-c-x3-h type family protein	S(4):100.0; T(8):94.7	22	1.372	1.375	1.489	0.650	0.580	0.630	1.412	0.143	0.620	0.001	0.002	Up-regulated by ABA
vPQDEEESGDDDEDEEADEHNNtLcGTcGTNDSk	B6TG72	phd finger protein	T(27):32.2	10	1.320	1.344	1.462	0.431	0.660	0.778	1.375	0.162	0.623	0.001	0.008	Up-regulated by ABA
sQPPDAAASPDASIssPSSLGGGGGDAADADAIEk	*K7UCK7	zinc finger ccch type domain-containing protein zfn-like 6	S(15):79.2	22	0.998	0.899	1.031	0.656	0.645	0.663	0.976	0.976	0.655	0.000	0.011	Up-regulated by ABA
Ribosomal protein																
gQAAATAsk	*B4FCE7	60s ribosomal protein l2	S(8):100.0	33	0.649	0.633	0.624	0.912	0,847	0.967	0.635	0.049	0.940	0.458	0.018	Down-regulated by osmotic stress with ABA-dependent way
asAAtSA	*O04014	tpa: 40s ribosomal protein s6	S(2):100.0; T(5):100.0	29	2.017	1.865	1.947	0.992	1.254	0.913	1.943	0.005	1.053	0.920	0.024	Up-regulated by osmotic stress with ABA-dependent way
vsEELR	*O04014	tpa: 40s ribosomal protein s6	S(2):100.0	16	1.773	1.541	1.476	0.981	0.989	1.121	1.597	0.044	1.030	0.358	0.046	Up-regulated by osmotic stress with ABA-dependent way
fTADDVAAAAGGAAAtGAsLQEID	*B6TPG2	60s ribosomal protein l26-1	T(16):100.0; S(19):100.0	20	0.456	0.387	0.523	1.301	1.299	1.286	0.455	0.000	1.295	0.075	0.003	Down-regulated by osmotic stress with ABA-dependent way
eEsDDDMGFSLFD	*B6UE07	60s acidic ribosomal protein p2a	S(3):100.0	44	1.920	2.079	1.631	1.013	0.995	1.174	1.877	0.003	1.061	0.261	0.049	Up-regulated by osmotic stress with ABA-dependent way
fASVPcGGGGVAVAAAsPAAGGAAPTAEAk	*B6UE07	60s acidic ribosomal protein p2a	S(17):80.0	27	0.498	0.487	0.536	1.371	1.104	1.240	0.507	0.002	1.238	0.127	0.010	Down-regulated by osmotic stress with ABA-dependent way
kAsGGGGDDEEEE	B4FCK4	40s ribosomal protein s9	S(3):100.0	19	0.987	1.082	1.020	1.560	1.487	1.550	1.030	0.643	1.532	0.004	0.010	Down-regulated by ABA
eEEkAPEPAEEsDEEMGFSLFDD	*B4FWI0	60s acidic ribosomal protein p0	S(12):100.0	12	—	—	—	1.752	1.687	1.681			1.706	0.000		Down-regulated by ABA
WD-40 repeat protein																
vSNNDSEPDsPSGSPNR	*B6TM01	transducin wd-40 repeat	S(10):100.0	49	1.608	1.264	1.921	1.070	0.804	1.020	1.598	0.022	0.965	0.801	0.043	Up-regulated by osmotic stress with ABA-dependent way
gRSsPVVsGSPSQNSDGSmsSWR	B4FMI7	wd repeat-containing protein 89 homolog	S(20):83.9	17	1.221	1.262	1.276	1.776	1.660	1.676	1.253	0.321	1.704	0.000	0.013	Down-regulated by ABA
ssPVVsGSPSQNSDGSmSSWR	B4FMI7	wd repeat-containing protein 89 homolog	S(2):96.0	12	3.083	3.880	3.912	1.240	1.022	1.012	3.625	0.000	1.091	0.701	0.018	Up-regulated by osmotic stress with ABA-dependent way
Arginine serine-rich splicing factor																
gNNGDDEHRGsPRGsQsP	C0HIN5	arginine serine-rich splicing factor rs2z37a transcript i	S(11):100.0; S(15):100.0; S(17):100.0	15	0.336	0.301	0.485	0.756	0.801	0.785	0.374	0.000	0.781	0.063	0.020	Down-regulated by osmotic stress with ABA-dependent way
sEGSSSsSFGR	*B7ZYN1	serine arginine repetitive matrix protein 2-like	S(7):79.6	27	0.601	0.552	0.705	0.850	0.798	0.844	0.620	0.038	0.831	0.190	0.028	Down-regulated by osmotic stress with ABA-dependent way
eRsPGAR	B6SY05	arginine serine-rich splicing factor rsp41	S(3):100.0	17	1.680	1.781	2.356	0.878	0.880	0.825	1.939	0.008	0.861	0.218	0.042	Up-regulated by osmotic stress with ABA-dependent way
gGtPPR	K7V1I2	arginine serine-rich splicing factor sr45_2 transcript i	T(3):100.0	18	0.310	0.308	0.387	0.859	0.949	0.827	0.335	0.000	0.878	0.234	0.011	Down-regulated by osmotic stress with ABA-dependent way
qYRsPsADR	K7U6X8; B4FD63	serine arginine repetitive matrix protein 2-like isoform x2	S(4):99.9; S(6):100.0	11	0.626	0.504	0.613	0.866	0.945	0.941	0.581	0.017	0.917	0.616	0.029	Down-regulated by osmotic stress with ABA-dependent way
aAcsGsP	*M1GS93	splicing arginine serine-rich 2	S(4):100.0; S(6):100.0	26	0.555	0.564	0.560	0.849	0.835	0.916	0.559	0.011	0.867	0.199	0.007	Down-regulated by osmotic stress with ABA-dependent way
sYTPDDINDR	*B4FQ73	serine arginine-rich splicing factor 33-like	S(1):100.0	11	1.789	1.954	1.864	0.977	1.010	0.945	1.869	0.007	0.977	0.654	0.002	Up-regulated by osmotic stress with ABA-dependent way
Heterogeneous nuclear ribonucleoprotein																
ssQGGGGYR	C0P8S9	heterogeneous nuclear ribonucleoprotein a2	S(2):100.0	12	0.597	0.567	0.685	0.812	0.845	0.815	0.616	0.029	0.824	0.114	0.040	Down-regulated by osmotic stress with ABA-dependent way
sPAGGQNYAmSR	*B8A134	heterogeneous nuclear ribonucleoprotein 1-like	S(1):100.0	12	0.587	0.543	0.618	0.897	0.834	0.847	0.583	0.015	0.859	0.202	0.008	Down-regulated by osmotic stress with ABA-dependent way
lGsPIGYVGLNDDSGSILSSMSR	*B8A134	heterogeneous nuclear ribonucleoprotein 1-like	S(3):95.1	13	2.221	2.340	2.179	0.987	1.021	1.163	2.247	0.001	1.057	0.289	0.006	Up-regulated by osmotic stress with ABA-dependent way
qPsEEPEEQVDLEGDDDGmDDDDAGYR	*K7UBY5	heterogeneous nuclear ribonucleoprotein r-like	S(3):100.0	74	1.789	1.801	1.894	0.867	0.887	1.080	1.828	0.010	0.945	0.845	0.002	Up-regulated by osmotic stress with ABA-dependent way
rGsRDDsEEPEEDDDNDER	*K7UBY5	heterogeneous nuclear ribonucleoprotein r-like	S(3):100.0; S(7):100.0	29	1.923	2.088	2.265	1.031	1.176	0.963	2.092	0.002	1.057	0.880	0.016	Up-regulated by osmotic stress with ABA-dependent way
dDsEEPEEDDDNDER	*K7UBY5	heterogeneous nuclear ribonucleoprotein r-like	S(3):100.0	55	1.577	1.779	2.056	1.208	1.302	1.436	1.804	0.002	1.315	0.880	0.021	Up-regulated by osmotic stress with ABA-dependent way
eANPGGsGGGR	B8A305	heterogeneous nuclear ribonucleoprotein 1-like isoform x1	S(7):100.0	11	0.565	0.612	0.671	1.210	0.988	1.102	0.616	0.031	1.100	0.508	0.028	Down-regulated by osmotic stress with ABA-dependent way

OS = osmotic stress.

^a^Each value represents the average of three biological replicas. A p value <0.05 in regard of FDR <0.05 is considered to be significant. Nevertheless, these peptides whose UniProt ID are signed with * are also significant under FDR <0.01. ‘-’, not measured. T-test is used to identify whether the difference is significant. A T-test value <0.05 is considered to be significant.

**Table 3:** Phosphorylation motif of proteins with significant phosphorylation sites in *Vp5* and *vp5* under osmotic stress.

Genotype	#	Motif	Motif Score	Foreground Matches	Foreground size	Background Matches	Background size	Fold Increase
	1	. . . . . . . . . . **S** P . . . . . p . . .	21.42	20	369	3885	1013205	14.14
	2	. . . . . . P . . . **S** P . . . . . . . . .	20.07	15	349	3609	1009320	12.02
	3	. . . . . . . . . . **S** P . . . . . . . . .	13.92	52	334	45568	1005711	3.44
	4	. . . . . . . . . R **S** . . . . . . . . P .	16.77	11	282	2404	960143	15.58
	5	. . . . . . . . . R **S** . . . . . . . . . .	7.15	36	271	46916	957739	2.71
	6	. . . . . . . . . G **S** . . . . . . . . . .	5.78	37	235	61833	910823	2.32
W	7	. . . . . . . A . D **S** . . . . . . . . . .	10.34	9	198	2666	848990	14.48
	8	. . . . . . . R . . **S** . . . . . . . . . .	5.42	27	189	45461	846324	2.66
	9	. . . . . . . . . . **S** S . . . . . A . . .	8.25	11	162	6216	800863	8.75
	10	. . . . . . . . . A**T** P . . . . . . . . .	18.63	9	101	1982	574595	25.83
	11	. . . . . . . . . . **T** P . . . . . . . . .	8.70	21	92	27413	572613	4.77
	12	P . . . . . . . . . **T** P . . . . . . . . .	5.06	14	71	26983	545200	3.98
	13	. . . . . A . . . . **T** . . . . . . . . . .	4.46	13	57	31931	518217	3.70
	14	. . . . . . . . . . **T** . S . . . . . . . .	3.95	13	44	44728	486286	3.21
M	1	. . . . . . . . . . S P . . . . . . . . .	11.70	35	167	53062	1013205	4.00
	2	. . . . . . . . . . S . . D . . . . . . .	7.76	26	132	53429	960143	3.54
	3	. . . . . . . . . . S . . . . . . G . . .	4.09	18	106	55755	906714	2.76
	4	. . . . . . . . . . T P . . . . . . . . .	7.50	15	52	29395	574595	5.64

**Table 4:** Comparison of different phosphopeptides belonging to one protein in response to ABA and osmotic stress.

Protein accession	Protein name	Sequence	*Vp5*: OS/control	*vp5*: OS/control
B4F8Q3	btb poz domain-containing protein at5g66560-like	dVADEGNEEEGsEAEtPGR	4.768	0.882
		aIAQTIMANEGGAAGsGEEGGEsDGGGTWR	0.635	1.307
B4FAG8	e3 ubiquitin-protein ligase rhf2a-like isoform x1	eVNAGIASVsR	0.613	0.852
		rHSTGQstPDR	2.039	1.302
B4FK28	tpa:rna-binding protein	sNTSsIGSPGPGR	0.597	0.866
		sPAGVGQNYAMNR	0.588	1.031
B4FWC4	rna-binding protein 39-like isoform x1	aVEPAPPQANGSGsGSGEkDR	2.213	1.009
		nLVQSNATsGGAASGGAR	0.645	1.036
B4FZY1	Na^+^/H^+^ antiporter	rPAsLR	0.605	1.040
		gFVPFVPGsPTESsLPLLPGNEN	2.913	0,954
B6SS20	tpa:phototropin family protein kinase	dALPAEVEAPAPAPAPAPPEsTTEk	2.021	1.046
		sEGEQEPVEPAPPVMAsPLVAPGtPSGGASLk	1.763	1.271
B6T245	zn- - containing protein	gsPmPVSsPWSGGALAENTDNIASR	1.714	1.045
		gsPMPVsSPWSGGALAENTDNIASR	0.509	1.224
B6T6V5	ubiquitin carboxyl-terminal hydrolase 6-like	eNEGSSSsAGESSSmDIDk	0.618	1.213
		sALLsYSDTVR	0.561	1.354
B6TDL6	uncharacterized membrane protein at1g16860-like	lSGPQsSGVNPmAR	0.595	0.991
		rLsGPQSsGVNPmAR	1.830	1.100
B6TI42	at-hook protein 1	qQQQQQLAPSPAPLNLAPTGVAAGPSsPPSR	0.575	0.923
		ePFGLPktPAtPPSSGGTQGLR	0.339	1.177
B6UBN4	j domain-containing protein required for chloro-plast accumulation response 1-like isoform x2	nDDGTSYAYsVPTsPNASMNNYLAQGAAR	0.454	1.006
		gMDSSmPtsPSQQMSNR	0.617	1.117
B6UE07	60s acidic ribosomal protein p2a	eEsDDDMGFSLFD	1.877	1.061
		fASVPcGGGGVAVAAAsPAAGGAAPTAEAk	0.507	1.238
B8A134	heterogeneous nuclear ribonucleoprotein 1-like	sPAGGQNYAmSR	0.583	0.859
		lGsPIGYVGLNDDSGSILSSMSR	2.247	1.057
B8A307	transmembrane expressed	dQEGGQPTGPEVVADDEVTsHR	0.513	0.956
		sNsVSTtGNENLR	1.725	1.092
C0HIM6	integrin-linked protein kinase family protein	qLsSGAAR	0.579	0.926
		gGPDGSsAHQQLAVPENLDATmR	0.312	1.050
C0HIQ2	something about silencing protein 10-like isoform x4	qIAGGDDsmDEQEDETQENVWGR	2.497	0.909
		qIAGGDDsMDEQEDETQENVWGR	0.617	1.212
C0P9I0	unknown	eTGDGEEGEEEDASAAtGDEVVk	1.979	1.139
		eTGDGEEGEEEDAsAAtGDEVVk	1.973	1.023
C0PJF1	basic proline-rich	sPSQQPPR	1.645	0.754
		rPPsPPAPAPPAAEELTEAGTEER	1.729	0.977
C0PM56	chloroplast post-illumination chlorophyll fluorescence increase protein	lDIVSGcTDPSSDmFDPLATVDDGScPLEsDSEE	1.677	1.051
		lDIVSGcTDPSSDMFDPLATVDDGScPLESDsEE	1.762	0.988
C4J2P1	protein kinase superfamily protein	asPEPGEVSGGR	1.729	0.992
		sVsPADSSVPGQWk	0.483	1.104
K7TFK8	e3 ubiquitin-protein ligase upl1-like	dVsNAsELATEMQYER	2.007	1.051
		lRPGQPDAVQDAStSDmEDASTSSGGQR	1.661	1.424
K7U2M6	heat shock protein sti	dVEPEPEAEPmDLtDEEk	0.541	1.084
		dVEPEPEAEPMDLtDEEk	0.242	0.988
K7U4E0	protein furry homolog isoform x1	asEmDAVGLVFLsSADVQIR	0.536	1.005
		sGQLLPALItmSGPLSGVR	0.581	1.376
K7UBY5	heterogeneous nuclear ribonucleoprotein r-like	qPsEEPEEQVDLEGDDDGmDDDDAGYR	1.828	0.945
		rGsRDDsEEPEEDDDNDER	2.091	1.057
		dDsEEPEEDDDNDER	1.804	1.515
K7UT89	jumonji-like transcription factor family protein	dTVAEDSAHATEEsGEENLQEk	2.399	1.208
		dTVAEDsAHATEEsGEENLQEk	2.924	1.240
K7V792	splicing factor 3b subunit 1-like isoform x1	mADADAtPAAGGATPGATPSGAWDAtPk	1.776	0.802
		lLAtPTPLGtPLYAIPEENR	0.537	0.942
		mADADAtPAAGGAtPGAtPSGAWDAtPk	0.319	1.127
K7VBC2	vacuolar proton atpase a1-like	fLGTSEmDPDSEPDsAR	1.861	0.982
		fLGTSEMDPDSEPDsAR	0.447	1.054
O48547	nonphototropic hypocotyl protein expressed	vsEELR	1.597	1.030
		ssETGsR	1.905	1.008
		eDPLLDsDDERPDsFDDDFR	1.774	1.109
Q6JN48	ethylene-insensitive protein 2-like	sIVDSTPYVSDDGPPsLTFSR	0.569	0.874
		sYYDPsSVDGNENAGSPAYSk	0.427	1.458
Q8W149	cell division cycle 5-like	eIQtPNPMAtPLAsPGPGItPR	1.752	1.030
		eIQtPNPMATPLAsPGPGITPR	2.139	0.932
Q9ATM4	aquaporin pip2-7	aLGSFRsNA	0.639	0.745
		aLGsFR	0.663	0.827
		aLGsFRsNA	0.406	0.869
B4F808	nucleic acid binding protein	eLALLNstLREDsPHPGsVsPFsNGGmkR		0.410
		eLALLNstLREDSPHPGsVsPFsNGGmkR		1.593
B6SVF2	gtp binding protein	asAEPLRFtVTPGDAFGDGPPVGmsEAAk	1.247	0.364
		asAEPLRFtVTPGDAFGDGPPVGMsEAAk	1.261	0.646
C0HF00	vacuolar protein sorting-associated protein 41 homolog	sNSGQDsDGGMDDEDGSPSGQSR	1.032	0.624
		sNSGQDsDGGmDDEDGSPSGQSR		1.808

**Table 5:** Phosphorylated chloroplast proteins in maize leaves regulated by ABA under osmotic stress.

Protein accession	Protein name	Sequence	Phosphorylation level/protein abundance	*Vp5*: OS/control	*vp5*: OS/control	Regulation of ABA and osmotic stress for peptides phosphosites
B4FAW3	photosystem i reaction center subunit ii	gFVAPQLDPSTPSPIFGGStGGLLR	Phosphorylation level	2.281	1.295	Up-regulated by osmotic stress with ABA-independent way
			Protein abundance	0.775	0.968	
B4FSE2	protochlorophyllide reductase b	aQAAAVSSPSVTPAsPSGk	Phosphorylation level	1.701	1.048	Up-regulated by osmotic stress with ABA-dependent way
			Protein abundance	0.935	0.935	
B4FVB8	serine threonine-protein kinase chloroplastic-like	tIkEsMDELNSQR	Phosphorylation level	1.509	0.943	Up-regulated by osmotic stress with ABA-dependent way
			Protein abundance	1.102	0.923	
B4FZ38	fructose–bisphosphatase	dGsPPR	Phosphorylation level	1.815	0.668	Up-regulated by osmotic stress with ABA-dependent way
			Protein abundance	—	—	
B4G1V3	ribonucleoprotein chloroplastic-like	gGGGGGGGGsFVDSGNk	Phosphorylation level	0.522	0.871	Down-regulated by osmotic stress with ABA-dependent way
			Protein abundance	0.990	0.893	
B6SS20	tpa:phototropin family protein kinase	dALPAEVEAPAPAPAPAPPEsTTEk	Phosphorylation level	2.021	1.046	Up-regulated by osmotic stress with ABA-dependent way
		sEGEQEPVEPAPPVMAsPLVAPGtPSGGASLk		1.763	1.271	Up-regulated by osmotic stress with ABA-independent way
			Protein abundance	1.087	1.015	
B6STN4	chlorophyll a-b binding protein 2	vGsFGEGR	Phosphorylation level	0.561	1.054	Down-regulated by osmotic stress with ABA-dependent way
			Protein abundance	1.621	0.863	
B6TM56	chloroplast outer envelope 24 kd protein	nSADGAGAADAEsR	Phosphorylation level	0.270	1.345	Down-regulated by osmotic stress with ABA-dependent way
			Protein abundance	0.597	1.537	
B6TS38	ribose-5-phosphate isomerase	gsAAAsPPPSGk	Phosphorylation level	0.618	1.031	Down-regulated by osmotic stress with ABA-independent way
			Protein abundance	1.037	1.011	
B6UBN4	j domain-containing protein required for chloroplast accumulation response 1-like isoform x2	nDDGTSYAYsVPTsPNASMNNYLAQGAAR	Phosphorylation level	0.454	1.006	Down-regulated by osmotic stress with ABA-dependent way
		gMDSSmPtsPSQQMSNR		0.617	1.117	Down-regulated by osmotic stress with ABA-dependent way
			Protein abundance	1.060	0.985	
C0PM56	chloroplast post-illumination chlorophyll fluorescence increase protein	lDIVSGcTDPSSDmFDPLATVDDGScPLEsDSEE	Phosphorylation level	1.677	1.051	Up-regulated by osmotic stress with ABA-dependent way
		lDIVSGcTDPSSDMFDPLATVDDGScPLESDsEE		1.762	0.988	Up-regulated by osmotic stress with ABA-dependent way
			Protein abundance	—	—	
C0PNN7	atp synthase gamma chain chloroplast (h(+)-transporting two-sector atpase f -atpase atpc1)	nLsIAYNR	Phosphorylation level	0.618	0.936	Down-regulated by osmotic stress with ABA-dependent way
			Protein abundance	—	—	
K7U926	stress enhanced protein chloroplastic-like isoform x2	sLsIIR	Phosphorylation level	1.824	1.034	Up-regulated by osmotic stress with ABA-dependent way
			Protein abundance	—	—	
K7VLY6	blue-light photoreceptor phr2-like	lNsAtYSVISPLPSSTPGLSR	Phosphorylation level	0.615	1.054	Down-regulated by osmotic stress with ABA-dependent way
			Protein abundance	—	—	
P22275	phosphoenolpyruvate carboxylase	Phosphorylation level	Phosphorylation level	2.248	1.090	Up-regulated by osmotic stress with ABA-dependent way
			Protein abundance	Protein abundance	0.983	0.987	
P31927	sucrose-phosphate synthase	gAGGGGGGGDPRsPTk	Phosphorylation level	0.603	1.074	Down-regulated by osmotic stress with ABA-dependent way	
			Protein abundance	1.002	0.985		
B4FQ59	phosphoribulokinase precursor	lTsVFGGAAEPPk	Phosphorylation level	0.980	1.577	Down-regulated by ABA	
			Protein abundance	1.113	0.932		
B6SVI8	protein lutein deficient chloroplastic-like	aATTPAmPAtGLssAGASPFR	Phosphorylation level	—	0.371	Up-regulated by ABA	
			Protein abundance	—	—		
B6T9S5	ferredoxin--nadp leaf isozyme	mAAVTAAAIsLsSSSASSxAAAAk	Phosphorylation level	0.889	1.591	Down-regulated by ABA	
			Protein abundance	—	—		
B6UBN4	j domain-containing protein required for chloroplast accumulation response 1-like isoform x2	nDDGTSYAYsVPtSPNASmNNYLAQGAAR	Phosphorylation level	—	1.952	Down-regulated by ABA	
			Protein abundance	1.060	0.985		
C4JAR6	rubisco subunit binding-protein beta subunit	sSEGTGSFPsPAAsPQPSR	Phosphorylation level	1.058	1.563	Down-regulated by ABA	
			Protein abundance	—	—		

**Table 6:** Kinases and phosphatases regulated by ABA under osmotic stress.

Protein accession	Protein name	Sequence	*Vp5*: OS/control	*vp5*: OS/control	Regulation of ABA and osmotic stress for peptides phosphosites
B4FAE7	tpa: protein kinase superfamily protein	dAGFQsAEEGGsGTFR	0.590	1.036	Down-regulated by osmotic stress with ABA-dependent way
B4FGQ3	probable receptor-like protein kinase at5g56460-like	aEsPkIQsPSER	0.614	1.231	Down-regulated by osmotic stress with ABA-dependent way
B4FVB8	serine threonine-protein kinase chloroplastic-like	tIkEsMDELNSQR	1.509	0.943	Up-regulated by osmotic stress with ABA-dependent way
B4FXH0	act-domain containing protein kinase family protein	iEDmDSAYDsDAsEEGDDDGDDLSVR	2.322	0.819	Up-regulated by osmotic stress with ABA-dependent way
B4FY41	protein kinase chloroplastic-like	rLSGsAsPLPAPAtGSPLPGSSR	1.900	0.925	Up-regulated by osmotic stress with ABA-dependent way
B4FZ38	fructose–bisphosphatase	dGsPPR	1.815	0.668	Up-regulated by osmotic stress with ABA-dependent way
B6SS20	tpa: phototropin family protein kinase	dALPAEVEAPAPAPAPAPPEsTTEk	2.021	1.046	Up-regulated by osmotic stress with ABA-dependent way
		sEGEQEPVEPAPPVMAsPLVAPGtPSGGASLk	1.763	1.271	Up-regulated by osmotic stress with ABA-independent way
B6SVR9	protein kinase	hsQPDLsGPPPPk	0.617	1.073	Down-regulated by osmotic stress with ABA-dependent way
B6SWV6	nad kinase 1	sLSPAPIPIPAsPGIR	3.890	1.049	Up-regulated by osmotic stress with ABA-dependent way
B6SXI8	tpa: protein kinase superfamily protein	sGPGPsFANR	0.516	0.986	Down-regulated by osmotic stress with ABA-independent way
B6SYP7	cdpk-related protein kinase	aDHDADPSGAGSVAPPsPLPANGAPLPAtPR	1.774	0.984	Up-regulated by osmotic stress with ABA-independent way
B7ZXP0	tpa: snrk sapk family protein kinase	sTVGTPAYIAPEVLLk	2.182	0.920	Up-regulated by osmotic stress with ABA-dependent way
B7ZYP6	pyruvate orthophosphate dikinase	sDsGAGR	0.589	0.999	Down-regulated by osmotic stress with ABA-dependent way
B7ZYR5	tpa: leucine-rich repeat receptor-like protein kinase family protein	aATSSAAAAAGsGATR	0.385	1.130	Down-regulated by osmotic stress with ABA-dependent way
		atsEEERSGGtPPAAPtP	1.000	1.574	Down-regulated by ABA
B8A0M9	tpa: map kinase family protein isoform 1	vAFNDTPTTVFWtDyVATR	2.941	1.211	Up-regulated by osmotic stress with ABA-dependent way
C0HIM6	integrin-linked protein kinase family protein	qLsSGAAR	0.579	0.926	Down-regulated by osmotic stress with ABA-independent way
		gGPDGSsAHQQLAVPENLDATmR	0.312	1.050	Down-regulated by osmotic stress with ABA-dependent way
C0P5V5	tpa: act-domain containing protein kinase family protein	gAsPPPPPSAGGAAGR	0.520	1.083	Down-regulated by osmotic stress with ABA-dependent way
C0P8J5	tpa: act-domain containing protein kinase family protein	sVQVSPILDGNQtDsDSNTAGEEVASR	2.738	1.136	Up-regulated by osmotic stress with ABA-dependent way
C0PKH3	serine threonine-protein kinase afc3-like	gGAsPPWR	0.627	1.080	Down-regulated by osmotic stress with ABA-dependent way
C0PKN2	phosphoglycerate dehydrogenase	gLVEPVsSTFVNLVNADYtAk	2.589	1.044	Up-regulated by osmotic stress with ABA-dependent way
C4IYD7	c-type lectin receptor-like tyrosine-protein kinase at1g52310-like isoform x1	sGtsTSATsPmLPLEVRtPR	0.416	0.955	Down-regulated by osmotic stress with ABA-dependent way
C4J2P1	protein kinase superfamily protein	asPEPGEVSGGR	1.729	0.992	Up-regulated by osmotic stress with ABA-dependent way
		sVsPADSSVPGQWk	0.483	1.104	Down-regulated by osmotic stress with ABA-dependent way
K7TWL5	casein kinase i	mATEsDsDSDAR	0.443	1.106	Down-regulated by osmotic stress with ABA-dependent way
K7UAY1	serine threonine-protein kinase ctr1	tNVDPSIsIPGFVsSQIDNPTTTk	0.365	1.180	Down-regulated by osmotic stress with ABA-dependent way
K7UJC3	serine threonine-protein kinase at5g01020-like	fmDPGLEAQysPRAAEAAAk	0.283	1.100	Down-regulated by osmotic stress with ABA-dependent way
K7UNQ6	proline-rich receptor-like protein kinase perk2-like	aSsSSTSAADPNPNk	0.370	1.050	Down-regulated by osmotic stress with ABA-dependent way
K7UW53	proline-rich receptor-like protein kinase perk1-like	fFGSYSSSDyDSGQYNEDmk	2.064	1.088	Up-regulated by osmotic stress with ABA-dependent way
K7VGC6	cyclin-dependent kinase family protein	iPDLNLQDGPmVLsPPR	1.597	1.047	Up-regulated by osmotic stress with ABA-dependent way
K7VN66	leucine-rich repeat receptor-like protein kinase family protein	gLTASGGDFTSsSk	0.534	1.005	Down-regulated by osmotic stress with ABA-dependent way
A0MBZ8	gck-like kinase mik	fSSYEDMSNSGTVVQTQNEDPEtPR	0.408	Up-regulated by ABA	
B4FQ59	phosphoribulokinase precursor	lTsVFGGAAEPPk	0.980	1.577	Down-regulated by ABA
C0PHB9	probable receptor-like protein kinase at5g56460-like	vSSTAkPEsPPkVQsPSEVDR	1.227	1.580	Down-regulated by ABA
K7VGC6	cyclin-dependent kinase family protein	iPDLNLQDGPMVLsPPR	1.230	0.649	Up-regulated by ABA
Q41790	calcium-dependent protein kinase	aPAPDsGR	1.133	1.575	Down-regulated by ABA
