# Supplementary material for: Quantitative iTRAQ-based proteomic analysis of phosphoproteins and ABA-regulated phosphoproteins in maize leaves under osmotic stress
Source: Sci Rep. 2015 Oct 27;5:15626. doi: 10.1038/srep15626 (PMC4650667; doi:10.1038/srep15626)
Supplement: Supplementary Information [file srep15626-s1.doc]

**Supplementary Information: Tables S1-S7**

**Quantitative iTRAQ-based proteomic analysis of phosphoproteins and ABA-regulated phosphoproteins in leaves of ABA-deficient mutant and wild-type maize under osmotic stress**

Running title: **ABA-regulated phosphoproteins in maize leaves**

Xiuli Hu, Nana Li, Liuji Wu, Chunqi Li, Tianxue Liu, Chaohai Li, Li Zhang, Wei Wang*

**Table S1**︱Proteins and its phosphoralation peptides with more than 1.5-fold expression only in maize wild-type *Vp5* under osmotic stress

| **UniProt ID** | **Protein name** | **Sequence of phosphorylation peptides** | **PhosphoRS-Site Probabilities**  **(>75%)** | **Ion score** | ***Vp5*: OS/control** | | | ***vp5*: OS/control** | | | ***Vp5*: OS/control** | | ***vp5*: OS/control** | | **T-test**  **P-value** | **Regulation of ABA and osmotic stress for peptides phosphosites** |
| --- | --- | --- | --- | --- | --- | --- | --- | --- | --- | --- | --- | --- | --- | --- | --- | --- |
|  |  |  |  |  | 1 | 2 | **3** | **1** | **2** | **3** | **Average** | **P-Value /FDR** | **Average** | **P-Value**  **/FDR** |  |  |
| *A6YRS1 | ent domain containing protein | sTGGLQASMSNNPQPIHDPVPsPtTSGR | S(22):100.0 | 27 | 0.182 | 0.443 | 0.292 | 1.167 | 1.319 | 0.915 | 0.306 | 0.000  /0.000 | 1.134 | 0.370  /0.602 | 0.016 | Down-regulated by osmotic stress with ABA-dependent way |
| *B4F8N9 | gpi-anchored isoform x1 | stPsSPTSVYSLPASFDR | S(1):80.0 | 34 | 0.534 | 0.516 | 0.498 | 0.979 | 1.064 | 1.124 | 0.516 | 0.046  ~~/0.060~~ | 1.056 | 0.859  /0.956 | 0.008 | Down-regulated by osmotic stress with ABA-dependent way |
| *B4F8Q3 | btb poz domain-containing protein at5g66560-like | dVADEGNEEEGsEAEtPGR | S(12):100.0; T(16): 100.0 | 19 | 4.556 | 5.297 | 4.452 | 0.876 | 0.896 | 0.875 | 4.768 | 0.000  /0.000 | 0.882 | 0.346  /0.583 | 0.004 | Up-regulated by osmotic stress with ABA-dependent way |
|  |  | aIAQTIMANEGGAAGsGEEGGEsDGGGTWR | S(23): 89.5 | 12 | 0.539 | 0.701 | 0.666 | 1.178 | 0.707 | 1.037 | 0.635 | 0.043  /~~0.058~~ | 1.307 | 0.817  /0.943 | 0.307 | Down-regulated by osmotic stress with ABA-independent way |
| *B4FAE7 | tpa: protein kinase superfamily protein | dAGFQsAEEGGsGTFR | S(6):100.0; S(12): 96.5 | 42 | 0.645 | 0.605 | 0.520 | 1.030 | 1.066 | 1.011 | 0.590 | 0.019  /0.032 | 1.036 | 0.875  /0.964 | 0.005 | Down-regulated by osmotic stress with ABA-dependent way |
| *B4FAG8 | e3 ubiquitin-protein ligase rhf2a-like isoform x1 | eVNAGIASVsR | S(10): 100.0 | 30 | 0.605 | 0.589 | 0.646 | 0.912 | 0.800 | 0.845 | 0.613 | 0.028  /0.043 | 0.852 | 0.196  /0.397 | 0.020 | Down-regulated by osmotic stress with ABA-dependent way |
|  |  | rHSTGQstPDR | T(8): 97.0 | 28 | 2.211 | 1.890 | 2.017 | 1.401 | 1.201 | 1.303 | 2.039 | 0.002  /0.006 | 1.302 | 0.057  /0.158 | 0.002 | Up-regulated by osmotic stress with ABA-dependent way |
| *B4FAW3 | photosystem i reaction center subunit ii | gFVAPQLDPSTPSPIFGGStGGLLR | T(20): 80.0 | 36 | 1.845 | 3.415 | 1.582 | 1.153 | 1.242 | 1.489 | 2.281 | 0.003  /0.008 | 1.295 | 0.027  /0.085 | 0.252 | Up-regulated by osmotic stress with ABA-independent way |
| *B4FC96 | zn- - containing protein | gsPMPVSSPWSGGALAENNDNIASR | S(2): 100.0 | 72 | 0.620 | 0.290 | 0.485 | 1.005 | 0.954 | 0.900 | 0.465 | 0.001  /0.002 | 0.953 | 0.469  /0.687 | 0.031 | Down-regulated by osmotic stress with ABA-dependent way |
| *B4FCC3 | dna-binding protein family-like | eAsEANDsEIQmEDVGk | S(3):100.0; S(8): 100.0 | 13 | 1.711 | 1.700 | 1.690 | 1.022 | 1.100 | 1.026 | 1.700 | 0.023  /0.037 | 1.049 | 0.875  /0.964 | 0.002 | Up-regulated by osmotic stress with ABA-dependent way |
| *B4FCE7 | 60s ribosomal protein l2 | gQAAATAsk | S(8): 100.0 | 33 | 0.649 | 0.633 | 0.624 | 0.912 | 0,847 | 0.967 | 0.635 | 0.049  ~~/0.061~~ | 0.940 | 0.458  /0.679 | 0.018 | Down-regulated by osmotic stress with ABA-dependent way |
| *B4FDJ3 | epidermal growth factor receptor substrate 15-like 1-like isoform x2 | sVFDsVPsTPMQR | S(5): 99.5 | 12 | 0.251 | 0.201 | 0.159 | 0.915 | 0.899 | 0.922 | 0.204 | 0.000  /0.000 | 0.912 | 0.467  /0.689 | 0.002 | Down-regulated by osmotic stress with ABA-dependent way |
| *B4FFB7 | dead-box atp-dependent rna helicase family protein | gGWDtDSEDR | T(5): 100.0 | 30 | 0.565 | 0.707 | 0.645 | 0.900 | 0.898 | 0.913 | 0.639 | 0.043  ~~/0.057~~ | 0.903 | 0.416  /0.638 | 0.024 | Down-regulated by osmotic stress with ABA-dependent way |
| *B4FFY6 | transposon protein | hDAEDSGNPSFssSDEQESAPR | S(12):76.6; S(13):76.6 | 35 | 1.820 | 1.942 | 1.839 | 1.077 | 1.116 | 1.007 | 1.864 | 0.008  /0.018 | 1.067 | 0.696  /0.863 | 0.001 | Up-regulated by osmotic stress with ABA-dependent way |
| B4FGQ3 | probable receptor-like protein kinase at5g56460-like | aEsPkIQsPSER | S(3):100.0; S(8): 96.2 | 11 | 0.613 | 0.599 | 0.631 | 1.219 | 1.323 | 1.152 | 0.614 | 0.028  /0.043 | 1.231 | 0.164  /0.355 | 0.009 | Down-regulated by osmotic stress with ABA-dependent way |
| B4FHK6 | ubiquitin-conjugating enzyme e2 22-like | ntPSmPPAVSTSSAsR | T(2):76.1 | 11 | 0.456 | 0.511 | 0.405 | 0.782 | 0.790 | 0.757 | 0.457 | 0.000  /0.002 | 0.776 | 0.053  /0.150 | 0.004 | Down-regulated by osmotic stress with ABA-dependent way |
| *B4FJ52 | bri1-kd interacting protein 128 | eTQEALDGSEETAsAGPDEDTSAVDVk | S(14): 96.1 | 89 | 1.867 | 1.540 | 1.694 | 1.149 | 1.335 | 1.279 | 1.700 | 0.031  /0.045 | 1.254 | 0.500  /0.713 | 0.096 | Up-regulated by osmotic stress with ABA-dependent way |
| *B4FK28 | tpa: rna-binding protein | sNTSsIGsPGPGR | S(8): 97.7 | 36 | 0.617 | 0.603 | 0.569 | 0.845 | 0.899 | 0.854 | 0.597 | 0.022  /0.036 | 0.866 | 0.272  /0.486 | 0.006 | Down-regulated by osmotic stress with ABA-dependent way |
|  |  | sPAGVGQNYAMNR | S(1): 100.0 | 14 | 0.599 | 0.534 | 0.631 | 0.977 | 1.112 | 1.003 | 0.588 | 0.018  /0.031 | 1.031 | 0.983  /0.994 | 0.023 | Down-regulated by osmotic stress with ABA-dependent way |
| B4FLK4 | zinc finger protein 207-like isoform x1 | dPAVGsSPAVsNNk | S(11): 100.0 | 16 | 0.431 | 0.423 | 0.454 | 1.277 | 1.292 | 1.311 | 0.436 | 0.000  /0.002 | 1.293 | 0.086  /0.713 | 0.000 | Down-regulated by osmotic stress with ABA-dependent way |
| B4FMI7 | wd repeat-containing protein 89 homolog | ssPVVsGSPSQNSDGSmSSWR | S(2): 96.0 | 12 | 3.083 | 3.880 | 3.912 | 1.240 | 1.022 | 1.012 | 3.625 | 0.000  /0.000 | 1.091 | 0.701  /0.860 | 0.018 | Up-regulated by osmotic stress with ABA-dependent way |
| B4FN51 | protein grpe-like | iGFSSVSPQQsDDEVNELk | S(11): 47.9 | 13 | 1.662 | 1.801 | 1.914 | 0.914 | 0.910 | 0.899 | 1.792 | 0.014  /0.026 | 0.908 | 0.487  /0.699 | 0.008 | Up-regulated by osmotic stress with ABA-dependent way |
| *B4FPT5 | uncharacterized protein LOC100272657 | sSQVSEtASsVSEENNQVDPDAPR | S(9): 99.4 | 42 | 0.560 | 0.679 | 0.650 | 1.123 | 1.270 | 1.212 | 0.630 | 0.043  ~~/0.058~~ | 1.202 | 0.097  /0.247 | 0.000 | Down-regulated by osmotic stress with ABA-dependent way |
| *B4FPV9 | actin-related protein 9-like | iPFLDDEEMPsPNQPLPPk | S(11): 100.0 | 36 | 0.509 | 0.573 | 0.578 | 1.073 | 1.091 | 0.767 | 0.553 | 0.008  /0.017 | 0.977 | 0.994  /1.000 | 0.070 | Down-regulated by osmotic stress with ABA-dependent way |
| *B4FQ71 | tpa: duf1421 domain family protein | sSsSVNGTSDDmLPSYDFQPIR | S(1):76.7 | 34 | 0.553 | 0.692 | 0.312 | 0.913 | 0.757 | 1.052 | 0.519 | 0.004  /0.010 | 0.907  /0.324 | 0.145 | 0.185 | Down-regulated by osmotic stress with ABA-independent way |
| *B4FQ73 | serine arginine-rich splicing factor 33-like | sYTPDDINDR | S(1): 100.0 | 11 | 1.789 | 1.954 | 1.864 | 0.977 | 1.010 | 0.945 | 1.869 | 0.007  /0.014 | 0.977 | 0.654  /0.829 | 0.002 | Up-regulated by osmotic stress with ABA-dependent way |
| *B4FQK4 | ist1-like isoform x2 | sPsLEDDPYFSYPNLFSAPkP | S(1):80.0;S(3): 80.0 | 30 | 1.603 | 1.536 | 1.913 | 1.017 | 0.891 | 1.158 | 1.684 | 0.027  /0.041 | 1.022 | 0.945  /0.979 | 0.006 | Up-regulated by osmotic stress with ABA-dependent way |
| *B4FQT3 | heat shock protein sti | iQRPEASEPSQSTPPPPPPQQQQtSPLQTk | T(24):80.0 | 14 | 0.647 | 0.544 | 0.655 | 0.971 | 0.787 | 0.874 | 0.615 | 0.048  ~~/0.060~~ | 0.877 | 0.294  /0.513 | 0.014 | Down-regulated by osmotic stress with ABA-dependent way |
| *B4FRM3 | fructose-bisphosphate aldolase | lSsINVENVEENR | S(3): 97.4 | 17 | 1.812 | 1.691 | 1.768 | 0.879 | 1.020 | 0.978 | 1.757 | 0.017  /0.030 | 0.959 | 0.616  /0.950 | 0.009 | Up-regulated by osmotic stress with ABA-dependent way |
| B4FRR8 | 6b-interacting protein 1 | eDcWSDGEtAALVSAWGSR | T(9): 95.8 | 11 | 0.532 | 0.569 | 0.551 | 1.133 | 1.140 | 1.156 | 0.551 | 0.008  /0.017 | 1.143 | 0.376  /0.603 | 0.000 | Down-regulated by osmotic stress with ABA-dependent way |
| *B4FRW3 | gbp17 type transcription partial | vQEPEAsDEDEYDDEDDEPQk | S(7): 100.0 | 55 | 1.462 | 1.562 | 2.122 | 1.399 | 0.985 | 1.298 | 1.716 | 0.023  /0.037 | 1.227 | 0.121  /0.288 | 0.161 | Up-regulated by osmotic stress with ABA-independent way |
| B4FS10 | TPA: hypothetical protein ZEAMMB73_767959 | aktTAGk | T(3): 100. | 17 | 0.334 | 0.345 | 0.329 | 0.853 | 0.981 | 0.789 | 0.336 | 0.000  /0.000 | 0.874 | 0.254  /0.469 | 0.009 | Down-regulated by osmotic stress with ABA-dependent way |
| B4FSD6 | ac transposase | mtPPVGNNTPssStIk | T(2): 92.5; S(11): 75.0; S(12): 75.0; T(14): 75.0 | 15 | 0.596 | 0.532 | 0.633 | 0.999 | 0.912 | 0.907 | 0.587 | 0.021  /0.035 | 0.939 | 0.443  /0.672 | 0.012 | Down-regulated by osmotic stress with ABA-dependent way |
| *B4FSE2 | protochlorophyllide reductase b | aQAAAVSSPSVTPAsPSGk | S(15): 100.0 | 43 | 1.948 | 1.531 | 1.625 | 1.151 | 1.096 | 0.898 | 1.701 | 0.022  /0.036 | 1.048 | 0.934  /0.982 | 0.028 | Up-regulated by osmotic stress with ABA-dependent way |
| *B4FTA7 | pgr5-like a isoform 1 | atEQQGQVQEQDDEVVDR | T(2): 100.0 | 48 | 1.783 | 1.479 | 1.998 | 0.940 | 0.989 | 0.811 | 1.754 | 0.017  /0.029 | 0.913 | 0.453  /0.680 | 0.053 | Up-regulated by osmotic stress with ABA-dependent way |
| B4FTR7 | tab2 protein | mIVPASPsPR | S(8): 99.8 | 20 | 0.534 | 0.588 | 0.542 | 1.011 | 0.798 | 0.823 | 0.555 | 0.006  /0.014 | 0.877 | 0.272  /0.486 | 0.056 | Down-regulated by osmotic stress with ABA-independent way |
| B4FTY8 | harpin inducing protein | lSVPAPGAANAsPFQPTVck | S(12): 86.7 | 11 | 1.900 | 1.654 | 1.756 | 0.823 | 0.761 | 0.921 | 1.770 | 0.014  /0.026 | 0.835 | 0.287  /0.508 | 0.006 | Up-regulated by osmotic stress with ABA-dependent way |
| *B4FUX9 | coiled-coil domain-containing protein 12-like | eDGsPTEEAEEVEDDGELPAmk | S(4): 96.6 | 26 | 0.456 | 0.691 | 0.569 | 0.908 | 0.711 | 1.010 | 0.572 | 0.013  /0.024 | 0.876 | 0.214  /0.420 | 0.166 | Down-regulated by osmotic stress with ABA-independent way |
| *B4FVB8 | serine threonine-protein kinase chloroplastic-like | tIkEsMDELNSQR | T(1):100.0; S(5): 100.0 | 25 | 1.451 | 1.590 | 1.486 | 0.997 | 0.961 | 0.872 | 1.509 | 0.037  /0.050 | 0.943 | 0.949  /0.980 | 0.010 | Up-regulated by osmotic stress with ABA-dependent way |
| *B4FWC4 | rna-binding protein 39-like isoform x1 | aVEPAPPQANGSGsGSGEkDR | SS(14): 79.0 | 17 | 1.469 | 1.923 | 3.246 | 1.132 | 0.812 | 1.082 | 2.213 | 0.001  /0.002 | 1.009 | 0.720  /0.871 | 0.151 | Up-regulated by osmotic stress with ABA-independent way |
|  |  | nLVQSNATsGGAASGGAR | S(9): 73.3 | 14 | 0.313 | 0.999 | 0.623 | 1.284 | 0.925 | 0.899 | 0.645 | 0.005  /0.011 | 1.036 | 0.571  /0.772 | 0.331 | Down-regulated by osmotic stress with ABA-independent way |
| *B4FX58 | dead-box atp-dependent rna helicase 56-like | eLPEQIDTstYMPS | S(14): 98.0 | 56 | 1.637 | 1.812 | 1.554 | 1.062 | 1.200 | 0.899 | 1.668 | 0.032  /0.046 | 1.054 | 0.840  /0.950 | 0.001 | Up-regulated by osmotic stress with ABA-dependent way |
| *B4FX96 | zinc finger ccch type domain-containing protein zfn-like 1 | dPSINQVAsPVAAPEPVGAILPk | S(9):100.0 | 27 | 0.655 | 0.776 | 0.516 | 1.016 | 0.877 | 0.769 | 0.649 | 0.047  ~~/0.060~~ | 0.887 | 0.358  /0.591 | 0.087 | Down-regulated by osmotic stress with ABA-independent way |
| *B4FXH0 | act-domain containing protein kinase family protein | iEDmDSAYDsDAsEEGDDDGDDLSVR | S(10):99.9; S(13): 99.9 | 51 | 2.110 | 1.531 | 3.326 | 0.768 | 0.935 | 0.752 | 2.322 | 0.002  /0.007 | 0.819 | 0.563  /0.767 | 0.001 | Up-regulated by osmotic stress with ABA-dependent way |
| *B4FXQ4 | ataxin-3 homolog | eHSDSQDsPAR | S(8): 99.9 | 15 | 0.323 | 0.422 | 0.615 | 0.913 | 0.687 | 1.072 | 0.453 | 0.001  /0.002 | 0.891 | 0.230  /0.432 | 0.044 | Down-regulated by osmotic stress with ABA-dependent way |
| *B4FXU1 | rna polymerase ii-associated factor 1 homolog | vEDIDQYsGEEYsE | S(8): 99.9; S(13): 99.9 | 46 | 0.489 | 0.414 | 0.528 | 0.719 | 0.996 | 1.010 | 0.477 | 0.021  /0.035 | 0.908 | 0.187  /0.391 | 0.050 | Down-regulated by osmotic stress with ABA-dependent way |
| *B4FXZ7 | heterogeneous nuclear ribonucleoprotein 1-like isoform x2 | nNsTGTIGEPFSAAGNTYEVNNR | S(3): 93.9; | 43 | 2.123 | 1.872 | 1.781 | 0.890 | 1.144 | 1.321 | 1.926 | 0.006  /0.013 | 1.118 | 0.363  /0.594 | 0.071 | Up-regulated by osmotic stress with ABA-independent way |
| *B4FY41 | protein kinase chloroplastic-like | rLsGsAsPLPAPAtGSPLPGSSR | S(3):97.5; T(14): 94.7; | 25 | 1.670 | 1.908 | 2.123 | 1.130 | 0.856 | 0.789 | 1.900 | 0.005  /0.012 | 0.925 | 0.218  /0.423 | 0.052 | Up-regulated by osmotic stress with ABA-dependent way |
| *B4FY62 | tpa: c3hc zinc finger-like family protein | lQPADsIEGTVIDRDcDEVDDAAQDSGAR | S(6): 99.9 | 18 | 0.422 | 0.493 | 0.412 | 1.112 | 1.030 | 0.980 | 0.442 | 0.000  /0.001 | 1.041 | 0.891  /0.965 | 0.006 | Down-regulated by osmotic stress with ABA-dependent way |
|  |  | dcDEVDDAAQDsGAR | S(12): 100.0 | 36 | 2.981 | 1.879 | 2.006 | 1.355 | 1.439 | 1.402 | 2.288 | 0.001  /0.003 | 1.399 | 0.017  /0.056 | 0.139 | Up-regulated by osmotic stress with ABA-independent way |
| B4FYD7 | ranbp1 domain containing protein | dGGDDtGGESEQk | T(6): 97.0 | 19 | 0.454 | 0.603 | 0.345 | 1.103 | 0.777 | 1.233 | 0.467 | 0.000  /0.001 | 1.038 | 0.512  /0.725 | 0.113 | Down-regulated by osmotic stress with ABA-independent way |
| *B4FYL6 | uncharacterized protein LOC100274014 | eTLEGsIPENDLNLSDSDVGQPGR | S(6): 99.4 | 29 | 0.335 | 0.644 | 0.513 | 0.686 | 0.912 | 1.182 | 0.497 | 0.036  /0.050 | 0.927 | 0.411  /0.634 | 0.072 | Down-regulated by osmotic stress with ABA-independent way |
| B4FZ13 | unknown | dTTGHsADGk | S(6): 100.0 | 10 | 0.090 | 0.302 | 0.240 | 0.891 | 1.323 | 1.233 | 0.211 | 0.000  /0.000 | 1.149 | 0.138  /0.312 | 0.005 | Down-regulated by osmotic stress with ABA-dependent way |
| B4FZ38 | fructose- -bisphosphatase | dGsPPR | S(3): 100.0 | 14 | 2.120 | 1.567 | 1.758 | 0.723 | 0.599 | 0.681 | 1.815 | 0.012  /0.023 | 0.668 | 0.004  /0.015 | 0.012 | Up-regulated by osmotic stress with ABA-dependent way |
| *B4FZ91 | cold induced | sSVALDEEQDcHTPGR | S(1):70.0 | 39 | 1.924 | 1.343 | 1.612 | 1.128 | 0.941 | 0.900 | 1.626 | 0.036  /0.050 | 0.990 | 0.875  /0.964 | 0.034 | Up-regulated by osmotic stress with ABA-dependent way |
| B4FZY1 | na+ h+ antiporter | rPAsLR | S(4): 100.0 | 14 | 0.641 | 0.621 | 0.553 | 1.032 | 0.988 | 1.099 | 0.605 | 0.048  ~~/0.060~~ | 1.040 | 0.880  /0.962 | 0.016 | Down-regulated by osmotic stress with ABA-dependent way |
|  |  | gFVPFVPGsPTESsLPLLPGNEN | S(9): 70.0; | 17 | 2.946 | 2.823 | 2.969 | 1.139 | 0.936 | 0.789 | 2.913 | 0.000  /0.000 | 0.954 | 0.396  /0.625 | 0.003 | Up-regulated by osmotic stress with ABA-dependent way |
| *B4G0Y5 | katanin p60 atpase-containing subunit | aDsTSSDAEEGk | S(3): 78.4 | 10 | 0.651 | 0.479 | 0.631 | 1.401 | 0.989 | 1.274 | 0.587 | 0.019  /0.032 | 1.221 | 0.085  /0.223 | 0.012 | Down-regulated by osmotic stress with ABA-dependent way |
| B4G0Z1 | e3 ubiquitin-protein ligase ubr7-like | lGVDVNtmPAITDk | T(12): 99.6 | 11 | 0.601 | 0.499 | 0.558 | 1.230 | 1.110 | 1.192 | 0.553 | 0.001  /0.004 | 1.177 | 0.221  /0.424 | 0.000 | Down-regulated by osmotic stress with ABA-dependent way |
| *B4G1E6 | pro-resilin precursor | dDDDsDDEk | S(5): 100.0 | 36 | 1.812 | 1.503 | 1.588 | 0.998 | 1.412 | 1.212 | 1.634 | 0.039  ~~/ 0.054~~ | 1.207 | 0.164  /0.355 | 0.179 | Up-regulated by osmotic stress with ABA-independent way |
| *B4G1V3 | ribonucleoprotein chloroplastic-like | gGGGGGGGGsFVDSGNk | S(10): 100.0 | 16 | 0.447 | 0.624 | 0.497 | 0.727 | 0.984 | 0.901 | 0.522 | 0.014  /0.027 | 0.871 | 0.190  /0.391 | 0.011 | Down-regulated by osmotic stress with ABA-dependent way |
| *B4G217 | peptidyl-prolyl cis-trans isomerase g-like isoform x4 | mTNsEQGR | S(4): 99.9 | 15 | 0.314 | 0.212 | 0.406 | 0.897 | 1.232 | 0.973 | 0.311 | 0.000  /0.000 | 1.034 | 0.851  /0.955 | 0.040 | Down-regulated by osmotic stress with ABA-dependent way |
| *B6SJN1 | auxin-repressed kda protein | rAQSTPSTPTtPVtPSSSTTPR | T(14): 92.2 | 31 | 3.034 | 1.266 | 1.871 | 1.050 | 1.141 | 0.853 | 2.057 | 0.006  /0.013 | 1.014 | 0.745  /0.899 | 0.192 | Up-regulated by osmotic stress with ABA-independent way |
| *B6SKU4;C0PIL1 | kinesin light chain-like protein | rLssPLPR | S(3): 97.6 | 23 | 0.433 | 0.558 | 0.597 | 0.881 | 0.824 | 1.028 | 0.523 | 0.030  /0.044 | 0.911 | 0.375  /0.605 | 0.888 | Down-regulated by osmotic stress with ABA-independent way |
| *B6SQN8 | protein phosphatase inhibitor 2 containing protein | tGsLtAEEADEDNR | S(3): 99.9; T(5): 100.0 | 11 | 2.012 | 1.501 | 2.709 | 1.212 | 1.059 | 1.383 | 2.074 | 0.002  /0.006 | 1.218 | 0.180  /0.380 | 0.079 | Up-regulated by osmotic stress with ABA-independent way |
| *B6SQQ5 | hypothetical protein | sQAQDsDAELQQPSPSPEVDPLAR | S(6): 99.8 | 14 | 2.187 | 3.630 | 2.801 | 1.123 | 0.899 | 0.970 | 2.873 | 0.000  /0.000 | 0.997 | 0.800  /0.934 | 0.060 | Up-regulated by osmotic stress with ABA-independent way |
| *B6SS20 | tpa: phototropin family protein kinase | dALPAEVEAPAPAPAPAPPEsTTEk | S(21):83.3 | 16 | 1.555 | 2.456 | 2.051 | 0.784 | 1.321 | 1.032 | 2.021 | 0.003  /0.007 | 1.046 | 0.853  /0.953 | 0.012 | Up-regulated by osmotic stress with ABA-dependent way |
|  |  | sEGEQEPVEPAPPVMAsPLVAPGtPSGGASLk | S(17):100.0; T(24): 100.0 | 38 | 2.111 | 1.681 | 1.499 | 0.977 | 1.700 | 1.138 | 1.763 | 0.019  /0.032 | 1.271 | 0.380  /0.602 | 0.284 | Up-regulated by osmotic stress with ABA-independent way |
| *B6SSK6;B4FA24 | nucleolar rna helicase 2 | sSsFGSR | S(3): 100.0 | 41 | 0.315 | 0.403 | 0.736 | 1.022 | 0.785 | 0.837 | 0.485 | 0.001  /0.004 | 0.881 | 0.138  /0.314 | 0.152 | Down-regulated by osmotic stress with ABA-independent way |
| B6SSY1 | calcium ion binding protein | kGsIDGAGNNNFDSFSEAGWSR | S(3): 96.0 | 20 | 2.989 | 4.194 | 1.709 | 1.234 | 1.785 | 0.982 | 2.964 | 0.001  /0.000 | 1.334 | 0.138  /0.121 | 0.080 | Up-regulated by osmotic stress with ABA-independent way |
| *B6STN4 | chlorophyll a-b binding protein 2 | vGsFGEGR | S(3): 100.0 | 32 | 0.578 | 0.445 | 0.659 | 1.094 | 1.003 | 1.064 | 0.561 | 0.016  /0.029 | 1.054 | 0.994  /1.000 | 0.008 | Down-regulated by osmotic stress with ABA-dependent way |
| *B6SVJ7 | duf21 domain-containing protein at1g47330-like | aDGsSPSYGSTAVSR | S(4): 96.3 | 13 | 0.343 | 0.214 | 0.398 | 1.077 | 0.783 | 1.232 | 0.318 | 0.000  /0.000 | 1.031 | 0.634  /0.812 | 0.012 | Down-regulated by osmotic stress with ABA-dependent way |
| B6SVR9 | protein kinase | hsQPDLsGPPPPk | S(2):100.0;S(7): 100.0 | 14 | 0.537 | 0.661 | 0.654 | 0.896 | 1.092 | 1.230 | 0.617 | 0.045  ~~/0.059~~ | 1.073 | 0.567  /0.771 | 0.019 | Down-regulated by osmotic stress with ABA-dependent way |
| *B6SW01 | zinc finger ccch type domain-containing protein zfn-like 3 | dSSANPPPsPGTTYGPVGSISk | S(9): 83.6 | 16 | 0.534 | 0.424 | 0.624 | 0.980 | 0.883 | 1.039 | 0.527 | 0.005  /0.012 | 0.967 | 0.698  /0.861 | 0.001 | Down-regulated by osmotic stress with ABA-dependent way |
| B6SW97 | protein fam188a-like | mSLQGsPPAQPEPk | S(6): 99.9 | 22 | 0.344 | 0.497 | 0.543 | 0.890 | 1.116 | 1.230 | 0.461 | 0.001  /0.002 | 1.079 | 0.469  /0.687 | 0.004 | Down-regulated by osmotic stress with ABA-dependent way |
| B6SWM4 | bel1-related homeotic protein 30 | qDLLtPNSYQGDDNR | T(5): 99.8 | 14 | 0.585 | 0.456 | 0.657 | 0.991 | 0.788 | 1.223 | 0.566 | 0.015  /0.027 | 1.001 | 0.908  /0.974 | 0.024 | Down-regulated by osmotic stress with ABA-dependent way |
| B6SWV6 | nad kinase 1 | sLSPAPIPIPAsPGIR | S(12): 100.0 | 15 | 3.760 | 2.977 | 4.933 | 1.234 | 0.789 | 1.123 | 3.890 | 0.000  /0.000 | 1.049 | 0.351  /0.585 | 0.029 | Up-regulated by osmotic stress with ABA-dependent way |
| *B6SX66 | uncharacterized protein LOC100275650 | qQQEAEEGsGPDEQEQEQQk | S(9): 100.0 | 58 | 1.745 | 2.141 | 2.867 | 0.988 | 1.593 | 0.835 | 2.251 | 0.001  /0.002 | 1.139 | 0.320  /0.547 | 0.139 | Up-regulated by osmotic stress with ABA-independent way |
| B6SXI8 | tpa: protein kinase superfamily protein | sGPGPsFANR | S(6): 100.0 | 16 | 0.390 | 0.602 | 0.556 | 1.234 | 0.765 | 0.959 | 0.516 | 0.003  /0.008 | 0.986 | 0.726  /0.872 | 0.143 | Down-regulated by osmotic stress with ABA-independent way |
| *B6SY05 | arginine serine-rich splicing factor rsp41 | eRsPGAR | S(3): 100.0 | 17 | 1.680 | 1.781 | 2.356 | 0.878 | 0.880 | 0.825 | 1.939 | 0.008  /0.018 | 0.861 | 0.218  /0.423 | 0.042 | Up-regulated by osmotic stress with ABA-dependent way |
| *B6SYP7 | cdpk-related protein kinase | aDHDADPSGAGSVAPPsPLPANGAPLPAtPR | S(17):93.8; T(29): 100.0 | 20 | 2.122 | 1.693 | 1.507 | 0.723 | 0.996 | 1.233 | 1.774 | 0.018  /0.031 | 0.984 | 0.926  /0.984 | 0.138 | Up-regulated by osmotic stress with ABA-independent way |
| B6SZ39 | hypothetical protein | iGytLQAISASStVYR | Y(3): 100.0; T(4): 100.0 | 14 | 3.234 | 2.752 | 1.999 | 0.789 | 1.012 | 0.823 | 2.662 | 0.000  /0.000 | 0.875 | 0.265  /0.480 | 0.040 | Up-regulated by osmotic stress with ABA-dependent way |
| *B6T0F0 | probable -trehalose-phosphate synthase | vmSVASPAsPTSPPAPAPPR | S(6): 80.0 | 16 | 1.700 | 1.580 | 1.553 | 0.878 | 1.067 | 1.222 | 1.611 | 0.041  ~~/0.056~~ | 1.056 | 0.681  /0.857 | 0.061 | Up-regulated by osmotic stress with ABA-independent way |
| B6T195 | mitochondrial import inner membrane translocase subunit tim14 | xtPLIAGLAVAAtALAGR | T(2):100.0 | 13 | 2.210 | 2.004 | 2.451 | 0.989 | 1.024 | 0.719 | 2.222 | 0.018  /0.031 | 0.911 | 0.163  /0.356 | 0.027 | Up-regulated by osmotic stress with ABA-independent way |
| *B6T245 | zn- - containing protein | gsPmPVSsPWSGGALAENTDNIASR | S(2): 100.0 | 20 | 1.642 | 1.999 | 1.501 | 1.048 | 0.877 | 1.211 | 1.714 | 0.022  /0.036 | 1.045 | 0.780  /0.918 | 0.111 | Up-regulated by osmotic stress with ABA-independent way |
|  |  | gsPMPVsSPWSGGALAENTDNIASR | S(2): 100.0; S(7): 96.2 | 56 | 0.551 | 0.350 | 0.626 | 0.813 | 0.782 | 2.077 | 0.509 | 0.005  /0.012 | 1.224 | 0.085  /0.223 | 0.194 | Down-regulated by osmotic stress with ABA-independent way |
| B6T2A6 | stem-specific protein tsjt1-like | iNsMPR | S(3): 100.0 | 18 | 0.563 | 0.305 | 0.445 | 0.748 | 0.655 | 0.823 | 0.438 | 0.000  /0.002 | 0.742 | 0.025  /0.079 | 0.037 | Down-regulated by osmotic stress with ABA-dependent way |
| B6T5Q3 | uncharacterized protein LOC100276146 | kcsTPAGGGGGGVGGGGGGGsDQPGk | S(21): 100.0 | 19 | 0.446 | 0.211 | 0.302 | 0.989 | 1.030 | 1.111 | 0.320 | 0.000  /0.000 | 1.043 | 0.755  /0.897 | 0.015 | Down-regulated by osmotic stress with ABA-dependent way |
| *B6T671 | uncharacterized loc101221005 | iSEQSNTLASSDsSTSSR | S(13): 78.2 | 15 | 1.499 | 1.908 | 1.502 | 1.093 | 0.899 | 1.212 | 1.637 | 0.047  ~~/0.060~~ | 1.068 | 0.537  /0.745 | 0.125 | Up-regulated by osmotic stress with ABA-dependent way |
| *B6T6H6 | dna binding protein | gQLsFSsR | S(4): 99.9; S(7): 97.1 | 17 | 2.023 | 1.545 | 1.883 | 1.230 | 0.899 | 1.158 | 1.817 | 0.011  /0.022 | 1.096 | 0.407  /0.631 | 0.003 | Up-regulated by osmotic stress with ABA-dependent way |
| *B6T6R3 | probable calcium-binding protein cml22-like | wAGLGTEDDDsDEASP | S(11): 96.3 | 47 | 0.389 | 0.527 | 0.578 | 1.001 | 1.209 | 0.877 | 0.498 | 0.002  /0.005 | 1.029 | 0.188  /0.389 | 0.046 | Down-regulated by osmotic stress with ABA-dependent way |
| *B6T6V5 | ubiquitin carboxyl-terminal hydrolase 6-like | eNEGSSSsAGESSSmDIDk | S(8): 79.5 | 17 | 0.612 | 0.584 | 0.658 | 1.312 | 1.113 | 1.215 | 0.618 | 0.029  /0.043 | 1.213 | 0.169  /0.363 | 0.008 | Down-regulated by osmotic stress with ABA-dependent way |
|  |  | sALLsYSDTVR | S(5): 75.0 | 13 | 0.580 | 0.592 | 0.512 | 1.799 | 1.064 | 1.200 | 0.561 | 0.008  /0.017 | 1.354 | 0.048  /0.136 | 0.070 | Down-regulated by osmotic stress with ABA-dependent way |
| *B6T727 | rna polymerase-associated protein leo1-like | kAVVFDDsDED | S(8): 100.0 | 31 | 0.534 | 0.637 | 0.641 | 1.210 | 1.081 | 1.034 | 0.604 | 0.024  /0.038 | 1.108 | 0.625  /0.810 | 0.029 | Down-regulated by osmotic stress with ABA-dependent way |
| *B6T7C2 | eukaryotic translation initiation factor 5 | aTTATGsDEDHssSPTRSHDGDk | S(18): 88.6 | 12 | 1.565 | 2.011 | 1.774 | 0.678 | 0.877 | 0.778 | 1.783 | 0.013  /0.025 | 0.777 | 0.055  /0.150 | 0.005 | Up-regulated by osmotic stress with ABA-dependent way |
| *B6T7F1 | ribonuclease p | yDNDEDAHsPGGR | S(9): 100.0 | 47 | 0.644 | 0.830 | 0.632 | 1.352 | 1.448 | 1.280 | 0.702 | 0.027  /0.041 | 1.360 | 0.035  /0.107 | 0.002 | Down-regulated by osmotic stress with ABA-dependent way |
| *B6T7I1 | rna binding protein | mmMESNPYFAVNAGsPLDVSk | Y(8): 99.4 | 28 | 0.456 | 0.667 | 0.680 | 0.879 | 1.171 | 0.966 | 0.601 | 0.044  ~~/0.058~~ | 1.005 | 0.943  /0.981 | 0.024 | Down-regulated by osmotic stress with ABA-dependent way |
| B6T7W0 | nucleolin 2-like | aEkPGAtxVWPAAGGAVtNASsSk | T(7):96.5;T(18): 96.5 | 15 | 3.312 | 2.721 | 2.566 | 0.890 | 1.187 | 1.230 | 2.866 | 0.000  /0.000 | 1.102 | 0.225  /0.429 | 0.034 | Up-regulated by osmotic stress with ABA-dependent way |
| *B6T883 | multidomain cystatin | sLEGAFVLNQHQPAEHDESSsQ | S(21): 96.2 | 15 | 0.302 | 0.447 | 0.453 | 0.999 | 1.105 | 1.119 | 0.401 | 0.000  /0.000 | 1.075 | 0.446  /0.672 | 0.000 | Down-regulated by osmotic stress with ABA-dependent way |
| B6TB14 | splicing arginine serine-rich 6 | dNGEGNGsDR | S(8): 100.0 | 16 | 0.457 | 0.733 | 0.627 | 1.222 | 1.136 | 0.913 | 0.605 | 0.026  /0.040 | 1.090 | 0.261  /0.477 | 0.078 | Down-regulated by osmotic stress with ABA-dependent way |
| *B6TC04 | fibrous sheath cabyr-binding | vDANEDsmEsSEEmVTPR | S(7):84.5;S(10): 74.5 | 15 | 2.122 | 1.754 | 1.523 | 1.072 | 1.018 | 1.031 | 1.800 | 0.011  /0.022 | 1.040 | 0.945  /0.979 | 0.042 | Up-regulated by osmotic stress with ABA-dependent way |
| B6TD33 | zinc finger ccch domain-containing protein 11-like | ePGEGtSS | T(6):83.3 | 17 | 1.594 | 1.623 | 1.856 | 1.226 | 1.112 | 1.345 | 1.691 | 0.027  /0.042 | 1.228 | 0.149  /0.331 | 0.010 | Up-regulated by osmotic stress with ABA-dependent way |
| *B6TDL6 | uncharacterized membrane protein at1g16860-like | lSGPQsSGVNPmAR | S(6):80.0 | 16 | 0.456 | 0.655 | 0.674 | 0.899 | 1.123 | 0.952 | 0.595 | 0.047  ~~/0.060~~ | 0.991 | 0.687  /0.860 | 0.022 | Down-regulated by osmotic stress with ABA-dependent way |
|  |  | rLsGPQSsGVNPmAR | S(3):100.0;S(7): 94.2 | 19 | 1.688 | 1.682 | 2.122 | 0.934 | 1.133 | 1.232 | 1.830 | 0.018  /0.031 | 1.100 | 0.880  /0.962 | 0.018 | Up-regulated by osmotic stress with ABA-dependent way |
| *B6TE60 | probable proteasome inhibitor-like | nsDGRsSPIQSSGDPGPR | S(2): 93.7 | 12 | 0.456 | 0.656 | 0.669 | 0.988 | 1.031 | 0.911 | 0.593 | 0.026  /0.040 | 0.977 | 0.458  /0.679 | 0.045 | Down-regulated by osmotic stress with ABA-dependent way |
| *B6TEF1 | rpm1-interacting protein 4-like isoform x4 | mINPNDPAENPEAFSVAAPsPPPGR | S(20): 100.0 | 53 | 1.652 | 1.765 | 1.598 | 0.993 | 1.152 | 1.038 | 1.678 | 0.019  /0.032 | 1.061 | 0.811  /0.942 | 0.002 | Up-regulated by osmotic stress with ABA-dependent way |
| *B6TEZ2 | hypothetical protein | aDGGVAsTSNSPR | S(7): 94.6 | 30 | 1.585 | 2.233 | 2.726 | 0.968 | 1.111 | 0.868 | 2.181 | 0.001  /0.003 | 0.983 | 0.497  /0.710 | 0.012 | Up-regulated by osmotic stress with ABA-dependent way |
| *B6TFI9 | gata transcription factor 25 | aSLEGESPAPGcDPGsQGSGLDFASR | S(16): 91.5 | 14 | 3.565 | 2.908 | 2.655 | 0.878 | 1.088 | 1.234 | 3.043 | 0.000  /0.000 | 1.066 | 0.580  /0.776 | 0.034 | Up-regulated by osmotic stress with ABA-dependent way |
| B6THF5 | iaa7 - auxin-responsive aux iaa family member | sAAAVVDtSLLSLGYsAPAFsPR | S(21): 98.2 | 14 | 2.092 | 2.012 | 2.533 | 1.324 | 1.253 | 1.312 | 2.212 | 0.001  /0.003 | 1.296 | 0.047  /0.134 | 0.013 | Up-regulated by osmotic stress with ABA-dependent way |
| *B6THP3 | transcription factor 21 | sGsmDGATsPFEGEsALSSGLPDYAk | S(3): 91.7 | 39 | 1.967 | 1.566 | 1.416 | 2.032 | 1.146 | 1.329 | 1.650 | 0.045  ~~/0.060~~ | 1.502 | 0.042  /0.122 | 0.412 | Up-regulated by osmotic stress with ABA-independent way |
| *B6TI42 | at-hook protein 1 | qQQQQQLAPSPAPLNLAPTGVAAGPSsPPSR | S(27): 78.9 | 47 | 0.546 | 0.520 | 0.661 | 0.888 | 0.962 | 0.720 | 0.575 | 0.024  /0.038 | 0.923 | 0.709  /0.865 | 0.276 | Down-regulated by osmotic stress with ABA-independent way |
|  |  | ePFGLPktPAtPPSSGGTQGLR | T(8):100.0; T(11): 100.0 | 12 | 0.212 | 0.403 | 0.402 | 0.998 | 1.323 | 1.209 | 0.339 | 0.000  /0.000 | 1.177 | 0.182  /0.383 | 0.002 | Down-regulated by osmotic stress with ABA-dependent way |
| *B6TJW1 | hypothetical protein | eGSQLEPDGsSAR | S(10):80. | 13 | 1.978 | 2.148 | 2.376 | 1.130 | 1.116 | 1.099 | 2.167 | 0.001  /0.003 | 1.115 | 0.469  /0.687 | 0.014 | Up-regulated by osmotic stress with ABA-dependent way |
| *B6TK35 | hypothetical protein | aVAVDSEQGSPEsPEQEk | S(13): 100.0 | 72 | 0.590 | 0.654 | 0.543 | 0.942 | 0.967 | 1.031 | 0.596 | 0.021  /0.035 | 0.980 | 0.715  /0.867 | 0.019 | Down-regulated by osmotic stress with ABA-dependent way |
| *B6TM01 | transducin wd-40 repeat | vSNNDSEPDsPSGSPNR | S(10): 100.0 | 49 | 1.608 | 1.264 | 1.921 | 1.070 | 0.804 | 1.020 | 1.598 | 0.022  /0.036 | 0.965 | 0.801  /0.933 | 0.043 | Up-regulated by osmotic stress with ABA-dependent way |
| *B6TM56 | chloroplast outer envelope 24 kd protein | nSADGAGAADAEsR | S(13): 100.0 | 18 | 0.314 | 0.192 | 0.303 | 1.380 | 1.233 | 1.423 | 0.270 | 0.000  /0.000 | 1.345 | 0.022  /0.072 | 0.000 | Down-regulated by osmotic stress with ABA-dependent way |
| *B6TPC9 | zn- - containing protein | gsPMLVsSPWSGGAIAENNDNIASR | S(2): 100.0; S(7): 96.4 | 95 | 0.151 | 0.248 | 0.230 | 1.184 | 0.926 | 1.118 | 0.210 | 0.000  /0.000 | 1.076 | 0.791  /0.926 | 0.014 | Down-regulated by osmotic stress with ABA-dependent way |
| *B6TPG2 | 60s ribosomal protein l26-1 | fTADDVAAAAGGAAAtGAsLQEID | T(16): 100.0; S(19): 100.0 | 20 | 0.456 | 0.387 | 0.523 | 1.301 | 1.299 | 1.286 | 0.455 | 0.000  /0.002 | 1.295 | 0.075  /0.202 | 0.003 | Down-regulated by osmotic stress with ABA-dependent way |
| *B6TRA9 | auxin-repressed protein | sESSsAPsSPSPASGAPDSPFGAATTPR | S(5): 88. | 22 | 3.032 | 2.944 | 2.797 | 1.466 | 1.356 | 1.372 | 2.924 | 0.000  /0.000 | 1.398 | 0.021  /0.068 | 0.001 | Up-regulated by osmotic stress with ABA-dependent way |
| *B6TS38 | ribose-5-phosphate isomerase | gsAAAsPPPSGk | S(2): 100.0; S(6): 100.0 | 37 | 0.621 | 0.988 | 0.244 | 1.212 | 1.013 | 0.867 | 0.618 | 0.013  /0.025 | 1.031 | 0.698  /0.861 | 0.167 | Down-regulated by osmotic stress with ABA-independent way |
| *B6TSD3 | tpa: arm repeat-containing protein containing family protein | sSsAINAAATELLDLsR | S(16): 100.0 | 19 | 0.556 | 0.373 | 0.646 | 1.433 | 1.033 | 1.111 | 0.525 | 0.004  /0.011 | 1.192 | 0.100  /0.253 | 0.030 | Down-regulated by osmotic stress with ABA-dependent way |
| *B6U194 | zinc finger c-x8-c-x5-c-x3-h type family protein | dWNQNFEVsPTDYLPQDSR | S(9): 80.0 | 12 | 0.401 | 0.393 | 0.536 | 0.901 | 0.993 | 0.821 | 0.443 | 0.000  /0.001 | 0.905 | 0.411  /0.634 | 0.038 | Down-regulated by osmotic stress with ABA-dependent way |
| *B6U1Z4 | atp binding protein | iEDIDPANDsDAsEEGDGDGDDLSVR | S(10):100.0; S(13): 100.0 | 54 | 2.299 | 2.427 | 2.469 | 0.978 | 1.076 | 0.978 | 2.398 | 0.000  /0.001 | 1.011 | 0.967  /0.987 | 0.001 | Up-regulated by osmotic stress with ABA-dependent way |
| B6U2V3 | dead-box atp-dependent rna helicase 38-like | aAsPEkk | S(3): 100.0 | 12 | 0.644 | 0.505 | 0.433 | 0.918 | 1.032 | 1.088 | 0.527 | 0.004  /0.011 | 1.013 | 0.487  /0.699 | 0.049 | Down-regulated by osmotic stress with ABA-dependent way |
| *B6U3A0 | glycine-rich rna-binding protein 7 | tGRYsPGSR | S(5): 99.9 | 19 | 0.357 | 0.386 | 0.484 | 0.873 | 1.130 | 0.869 | 0.409 | 0.000  /0.000 | 0.968 | 0.163  /0.356 | 0.035 | Down-regulated by osmotic stress with ABA-dependent way |
| *B6U4F1 | gbp18 type transcription partial | sAPHAGGMNt | T(10): 100.0 | 26 | 0.454 | 0.602 | 0.589 | 1.001 | 0.899 | 0.958 | 0.548 | 0.006  /0.014 | 0.953 | 0.715  /0.869 | 0.032 | Down-regulated by osmotic stress with ABA-dependent way |
| *B6U6U2 | hexose transporter | gQsALGsALGLIsR | S(3):100.0;S(7): 100.0;S(13): 100.0 | 77 | 1.683 | 1.692 | 1.634 | 1.071 | 1.048 | 1.095 | 1.670 | 0.026  /0.041 | 1.071 | 0.580  /0.776 | 0.003 | Up-regulated by osmotic stress with ABA-dependent way |
| B6U8H2 | atp binding protein | iIGATDsDSR | S(7): 97.6 | 10 | 2.225 | 1.599 | 1.853 | 1.210 | 0.800 | 1.023 | 1.892 | 0.007  /0.015 | 1.011 | 0.885  /0.963 | 0.006 | Up-regulated by osmotic stress with ABA-dependent way |
| B6U937 | probable sugar phosphate/phosphate translocator at3g17430-like | tQtGSsSNR | S(6): 79.5 | 10 | 6.023 | 4.674 | 3.324 | 0.758 | 0.813 | 0.869 | 4.674 | 0.000  /0.000 | 0.813 | 0.106  /0.263 | 0.041 | Up-regulated by osmotic stress with ABA-dependent way |
| *B6UB08 | zinc finger protein 652-a- partial | dLVVDtDDGGNANR | T(6): 100.0 | 46 | 0.570 | 0.521 | 0.564 | 0.809 | 0.812 | 1.089 | 0.552 | 0.006  /0.013 | 0.903 | 0.101  /0.252 | 0.057 | Down-regulated by osmotic stress with ABA-independent way |
| *B6UBN4 | j domain-containing protein required for chloroplast accumulation response 1-like isoform x2 | nDDGTSYAYsVPTsPNASMNNYLAQGAAR | S(14): 91.7 | 40 | 0.461 | 0.401 | 0.501 | 1.027 | 0.890 | 1.100 | 0.454 | 0.000  /0.001 | 1.006 | 0.891  /0.965 | 0.003 | Down-regulated by osmotic stress with ABA-dependent way |
|  |  | gMDSSmPtsPSQQMSNR | S(9): 99.4 | 13 | 0.556 | 0.634 | 0.662 | 1.121 | 1.034 | 1.196 | 0.617 | 0.050  /0.062 | 1.117 | 0.200  /0.396 | 0.010 | Down-regulated by osmotic stress with ABA-dependent way |
| *B6UE07 | 60s acidic ribosomal protein p2a | eEsDDDMGFSLFD | S(3): 100.0 | 51 | 1.920 | 2.079 | 1.631 | 1.013 | 0.995 | 1.174 | 1.877 | 0.003  /0.008 | 1.061 | 0.261  /0.477 | 0.049 | Up-regulated by osmotic stress with ABA-dependent way |
|  |  | fASVPcGGGGVAVAAAsPAAGGAAPTAEAk | S(17): 80.0 | 27 | 0.498 | 0.487 | 0.536 | 1.371 | 1.104 | 1.240 | 0.507 | 0.002  /0.006 | 1.238 | 0.127  /0.298 | 0.010 | Down-regulated by osmotic stress with ABA-dependent way |
| *B6UEI4 | protein gdap2 homolog | tVSANLVDLPLSNSGSALk | T(1):80.0 | 51 | 1.771 | 2.008 | 1.464 | 0.787 | 0.824 | 0.712 | 1.748 | 0.018  /0.031 | 0.774 | 0.041  /0.121 | 0.016 | Up-regulated by osmotic stress with ABA-dependent way |
| *B6UEP1 | transcription factor hy5 | rGSGSTAsGEGHGQ | S(8): 100.0 | 15 | 4.523 | 3.565 | 3.707 | 0.734 | 0.899 | 0.763 | 3.932 | 0.000  /0.000 | 0.798 | 0.041  /0.121 | 0.011 | Up-regulated by osmotic stress with ABA-dependent way |
| B6UHY2 | autophagy-related protein 18b-like | eFNLLDAySGSPk | Y(8): 83.3 | 18 | 1.864 | 2.453 | 1.734 | 0.734 | 0.845 | 0.766 | 2.017 | 0.004  /0.010 | 0.782 | 0.018  /0.061 | 0.023 | Up-regulated by osmotic stress with ABA-dependent way |
| B6UIQ0 | histone h2a | aDIGsASQEF | S(5): 80.0 | 25 | 1.580 | 1.634 | 1.571 | 0.979 | 1.231 | 1.188 | 1.595 | 0.048  ~~/0.061~~ | 1.133 | 0.218  /0.422 | 0.022 | Up-regulated by osmotic stress with ABA-dependent way |
| *B7ZXP0 | tpa: snrk sapk family protein kinase | sTVGTPAYIAPEVLLk | S(1):82.9 | 25 | 2.465 | 1.987 | 2.093 | 0.887 | 1.022 | 0.852 | 2.182 | 0.001  /0.004 | 0.920 | 0.227  /0.432 | 0.019 | Up-regulated by osmotic stress with ABA-dependent way |
| *B7ZXU2 | serrate-related c2h2 zinc-finger family protein | lGGsDGNsEDDMDNDk | S(4):100.0; S(8): 100.0 | 30 | 1.694 | 1.721 | 1.417 | 1.220 | 1.179 | 1.274 | 1.611 | 0.048  ~~/0.061~~ | 1.224 | 0.151  /0.333 | 0.088 | Up-regulated by osmotic stress with ABA-independent way |
| *B7ZYN1 | serine arginine repetitive matrix protein 2-like | sEGSSSsSFGR | S(7): 79.6 | 27 | 0.601 | 0.552 | 0.705 | 0.850 | 0.798 | 0.844 | 0.620 | 0.038  ~~/0.053~~ | 0.831 | 0.190  /0.391 | 0.028 | Down-regulated by osmotic stress with ABA-dependent way |
| *B7ZYP6 | pyruvate orthophosphate dikinase | sDsGAGR | S(3): 100.0 | 36 | 0.598 | 0.555 | 0.614 | 1.030 | 1.002 | 0.965 | 0.589 | 0.000  /0.000 | 0.999 | 0.937  /0.981 | 0.005 | Down-regulated by osmotic stress with ABA-dependent way |
| *B7ZYR5 | tpa: leucine-rich repeat receptor-like protein kinase family protein | aATSSAAAAAGsGATR | S(12):80.0 | 20 | 0.469 | 0.388 | 0.299 | 1.257 | 0.899 | 1.233 | 0.385 | 0.000  /0.000 | 1.130 | 0.109  /0.268 | 0.027 | Down-regulated by osmotic stress with ABA-dependent way |
| *B7ZZ27 | spf1-like dna-binding protein | dGNSSAFDQNEQSNDTTSGLsGAk | S(21): 100.0 | 84 | 0.585 | 0.672 | 0.451 | 1.123 | 1.041 | 0.823 | 0.569 | 0.012  /0.023 | 0.996 | 0.832  /0.953 | 0.017 | Down-regulated by osmotic stress with ABA-dependent way |
| B8A0K2 | calmodulin-binding family protein | vASPtSPLAPVQASLPQPS | T(5): 78.9 | 13 | 1.833 | 1.655 | 1.689 | 1.293 | 1.223 | 1.423 | 1.726 | 0.024  /0.038 | 1.313 | 0.074  /0.201 | 0.035 | Up-regulated by osmotic stress with ABA-dependent way |
| B8A0M9 | tpa: map kinase family protein isoform 1 | vAFNDTPTTVFWtDyVATR | T(13): 90.9 | 17 | 3.123 | 2.722 | 2.978 | 1.312 | 0.889 | 1.432 | 2.941 | 0.000  /0.000 | 1.211 | 0.169  /0.363 | 0.003 | Up-regulated by osmotic stress with ABA-dependent way |
| *B8A134 | heterogeneous nuclear ribonucleoprotein 1-like | sPAGGQNYAmSR | S(1): 100.0 | 12 | 0.587 | 0.543 | 0.618 | 0.897 | 0.834 | 0.847 | 0.583 | 0.015  /0.028 | 0.859 | 0.202  /0.398 | 0.008 | Down-regulated by osmotic stress with ABA-dependent way |
|  |  | lGsPIGYVGLNDDSGSILSSMSR | S(3): 95.1 | 13 | 2.221 | 2.340 | 2.179 | 0.987 | 1.021 | 1.163 | 2.247 | 0.001  /0.003 | 1.057 | 0.289  /0.509 | 0.006 | Up-regulated by osmotic stress with ABA-dependent way |
| *B8A1S1 | uncharacterized membrane protein at1g16860-like | qNsGPLPPVLPtTGLITSGPISSGPLNSSGAPR | S(3): 97.5 | 10 | 1.566 | 2.133 | 1.811 | 1.488 | 1.494 | 1.433 | 1.837 | 0.009  /0.019 | 1.472 | 0.009  /0.032 | 0.153 | Up-regulated by osmotic stress with ABA-independent way |
| *B8A287 | c2 domain-containing expressed | tSPsPSSSAAk | S(4): 100.0 | 22 | 1.885 | 1.678 | 1.657 | 1.029 | 1.131 | 1.155 | 1.740 | 0.042  ~~/0.057~~ | 1.105 | 0.838  /0.953 | 0.029 | Up-regulated by osmotic stress with ABA-dependent way |
| *B8A2I8 | lish domain-containing | sDssTTSTQSFAFPVLQR | S(1): 94.2 | 11 | 0.634 | 0.644 | 0.618 | 1.233 | 1.034 | 0.999 | 0.632 | 0.043  ~~/0.058~~ | 1.089 | 0.853  /0.953 | 0.023 | Down-regulated by osmotic stress with ABA-dependent way |
| B8A305 | heterogeneous nuclear ribonucleoprotein 1-like isoform x1 | eANPGGsGGGR | S(7): 100.0 | 11 | 0.565 | 0.612 | 0.671 | 1.210 | 0.988 | 1.102 | 0.616 | 0.031  /0.045 | 1.100 | 0.508  /0.722 | 0.028 | Down-regulated by osmotic stress with ABA-dependent way |
| *B8A307 | transmembrane expressed | dQEGGQPTGPEVVADDEVTsHR | S(20): 80.0 | 37 | 0.585 | 0.304 | 0.650 | 1.078 | 0.812 | 0.978 | 0.513 | 0.004  /0.010 | 0.956 | 0.358  /0.591 | 0.017 | Down-regulated by osmotic stress with ABA-dependent way |
|  |  | sNsVSTtGNENLR | S(3): 95.6 | 31 | 1.793 | 1.729 | 1.653 | 1.137 | 1.052 | 1.088 | 1.725 | 0.012  /0.023 | 1.092 | 0.558  /0.765 | 0.003 | Up-regulated by osmotic stress with ABA-dependent way |
| *B8A367 | cysteine chloroplastic chromoplastic-like | lAPcPsLVR | S(6): 100.0 | 25 | 0.468 | 0.456 | 0.479 | 0.870 | 0.875 | 0.849 | 0.468 | 0.000  /0.002 | 0.865 | 0.199  /0.398 | 0.001 | Down-regulated by osmotic stress with ABA-dependent way |
| *B8A396 | bud13 homolog | dtPsPVHGDAAAk | T(2): 100.0; S(4): 100.0 | 16 | 0.604 | 0.654 | 0.583 | 1.076 | 0.984 | 1.119 | 0.614 | 0.044  ~~/0.058~~ | 1.060 | 0.720  /0.868 | 0.018 | Down-regulated by osmotic stress with ABA-dependent way |
| *B8A3C8 | nucleotide binding protein | ekPASQGsQPsTPSASSGMR | S(8): 99.2 | 12 | 1.700 | 1.650 | 1.730 | 1.223 | 1.134 | 0.994 | 1.693 | 0.030  /0.044 | 1.117 | 0.926  /0.984 | 0.019 | Up-regulated by osmotic stress with ABA-dependent way |
| C0HE85 | eukaryotic translation initiation factor 2a-like | kAAEASGsPADES | S(8): 96.8 | 19 | 0.254 | 0.437 | 0.365 | 0.987 | 1.086 | 1.232 | 0.352 | 0.000  /0.000 | 1.102 | 0.607  /0.802 | 0.007 | Down-regulated by osmotic stress with ABA-dependent way |
| *C0HE93 | tumor susceptibility gene 101 family protein | sPPPSSSAGGAQYAQQFLNTALSQR | S(1): 100.0 | 78 | 0.269 | 0.350 | 0.301 | 0.922 | 1.068 | 0.995 | 0.307 | 0.000  /0.000 | 0.995 | 0.897  /0.966 | 0.030 | Down-regulated by osmotic stress with ABA-dependent way |
| *C0HHJ0 | set domain protein sdg111 | tRSNIsLDGDDDPYSGNQTER | T(1): 98.1 | 24 | 0.288 | 0.452 | 0.424 | 1.052 | 0.962 | 0.902 | 0.388 | 0.000  /0.000 | 0.972 | 0.771  /0.912 | 0.023 | Down-regulated by osmotic stress with ABA-dependent way |
| *C0HHU2 | Uncharacterized protein | aHGTAVGLPsDDDMGNSEVGHNALGAGR | S(10):80.0 | 11 | 0.422 | 0.543 | 0.593 | 0.799 | 0.889 | 0.991 | 0.520 | 0.004  /0.010 | 0.877 | 0.350  /0.587 | 0.001 | Down-regulated by osmotic stress with ABA-dependent way |
| *C0HIM6 | integrin-linked protein kinase family protein | qLsSGAAR | S(3): 98.4 | 31 | 0.614 | 0.690 | 0.434 | 0.897 | 0.870 | 1.011 | 0.579 | 0.016  /0.029 | 0.926 | 0.317  /0.545 | 0.100 | Down-regulated by osmotic stress with ABA-independent way |
|  |  | gGPDGSsAHQQLAVPENLDATmR | S(7):80.0 | 16 | 0.239 | 0.499 | 0.200 | 1.016 | 1.234 | 0.899 | 0.312 | 0.000  /0.000 | 1.050 | 0.558  /0.767 | 0.001 | Down-regulated by osmotic stress with ABA-dependent way |
| C0HIN5 | arginine serine-rich splicing factor rs2z37a transcript i | gNNGDDEHRGsPRGsQsP | S(11):100.0; S(15):100.0; S(17): 100.0 | 15 | 0.336 | 0.301 | 0.485 | 0.756 | 0.801 | 0.785 | 0.374 | 0.000  /0.000 | 0.781 | 0.063  /0.175 | 0.020 | Down-regulated by osmotic stress with ABA-dependent way |
| *C0HIQ2 | something about silencing protein 10-like isoform x4 | qIAGGDDsmDEQEDETQENVWGR | S(8): 97.2 | 39 | 2.677 | 2.247 | 2.567 | 0.733 | 0.983 | 1.011 | 2.497 | 0.000  /0.001 | 0.909 | 0.643  /0.820 | 0.015 | Up-regulated by osmotic stress with ABA-dependent way |
|  |  | qIAGGDDsMDEQEDETQENVWGR | S(8): 95.7 | 26 | 0.476 | 0.721 | 0.654 | 1.234 | 1.362 | 1.041 | 0.617 | 0.032  /0.046 | 1.212 | 0.227  /0.430 | 0.032 | Down-regulated by osmotic stress with ABA-dependent way |
| *C0HIV2 | serine hydroxymethyltransferase 4-like | nAVFGDSSALsPGGVR | S(11): 100.0 | 42 | 0.481 | 0.656 | 0.611 | 1.210 | 0.812 | 0.980 | 0.583 | 0.017  /0.029 | 1.001 | 0.851  /0.955 | 0.130 | Down-regulated by osmotic stress with ABA-independent way |
| C0P2B1 | phd zinc finger | vEsSLVGSDDVLDSASDSPPsVk | S(21): 74.8 | 15 | 2.334 | 2.410 | 2.309 | 1.350 | 1.597 | 1.441 | 2.351 | 0.000  /0.001 | 1.463 | 0.008  /0.029 | 0.003 | Up-regulated by osmotic stress with ABA-dependent way |
| C0P3H1 | ubiquitin carboxyl-terminal hydrolase isozyme l5-like | sAsPSTS | S(3): 96.4 | 16 | 0.555 | 0.512 | 0.597 | 0.957 | 0.879 | 1.020 | 0.555 | 0.006  /0.013 | 0.952 | 0.704  /0.860 | 0.002 | Down-regulated by osmotic stress with ABA-dependent way |
| C0P425 | udp-glycosyltransferase 73c5-like | mAIEDVANGRPtANGSGcDVk | T(12):50.0; S(16): 50.0 | 12 | 0.439 | 0.388 | 0.462 | 1.069 | 1.338 | 0.987 | 0.430 | 0.000  /0.001 | 1.131 | 0.667  /0.841 | 0.032 | Down-regulated by osmotic stress with ABA-dependent way |
| *C0P5V5 | tpa: act-domain containing protein kinase family protein | gAsPPPPPSAGGAAGR | S(3): 100.0 | 20 | 0.568 | 0.481 | 0.511 | 1.158 | 1.013 | 1.077 | 0.520 | 0.001  /0.002 | 1.083 | 0.616  /0.807 | 0.001 | Down-regulated by osmotic stress with ABA-dependent way |
| *C0P8G0 | ataxin-2 c-terminal region family expressed | sQAAILsPtAGSmSPNAAPWVk | S(7): 99.2; T(9): 90.3 | 51 | 0.524 | 0.458 | 0.299 | 1.312 | 1.205 | 0.892 | 0.427 | 0.000  /0.001 | 1.136 | 0.198  /0.398 | 0.007 | Down-regulated by osmotic stress with ABA-dependent way |
| *C0P8J5 | tpa: act-domain containing protein kinase family protein | sVQVSPILDGNQtDsDSNTAGEEVASR | S(15): 96.2 | 79 | 2.607 | 2.539 | 3.067 | 1.210 | 0.968 | 1.231 | 2.738 | 0.001  /0.003 | 1.136 | 0.023  /0.074 | 0.006 | Up-regulated by osmotic stress with ABA-dependent way |
| C0P8S9 | heterogeneous nuclearribonucleoprotein a2 | ssQGGGGYR | S(2): 100.0 | 12 | 0.597 | 0.567 | 0.685 | 0.812 | 0.845 | 0.815 | 0.616 | 0.029  /0.044 | 0.824 | 0.114  /0.276 | 0.040 | Down-regulated by osmotic stress with ABA-dependent way |
| *C0P9I0 | unknown | eTGDGEEGEEEDASAAtGDEVVk | T(17): 99.9 | 32 | 1.788 | 2.152 | 1.998 | 1.234 | 0.975 | 1.209 | 1.979 | 0.004  /0.011 | 1.139 | 0.967  /0.987 | 0.044 | Up-regulated by osmotic stress with ABA-dependent way |
|  |  | eTGDGEEGEEEDAsAAtGDEVVk | S(14):100.0; T(17): 100.0 | 61 | 2.165 | 1.965 | 1.788 | 1.111 | 1.067 | 0.892 | 1.973 | 0.004  /0.010 | 1.023 | 0.620  /0.811 | 0.003 | Up-regulated by osmotic stress with ABA-dependent way |
| *C0P9I5 | tbc1 domain family member 15-like | sLSSLELPGVASVANAMsR | S(18): 99.9 | 37 | 0.571 | 0.456 | 0.658 | 1.018 | 0.999 | 0.871 | 0.562 | 0.011  /0.022 | 0.963 | 0.934  /0.982 | 0.055 | Down-regulated by osmotic stress with ABA-independent way |
| *C0P9K3 | inner membrane protein albino3 | aLAAGDSNAsSSTYDMEDEESDDETTEEGGPVEEASSTGSDk | S(10):42.3; S(11): 42.3 | 69 | 3.010 | 2.243 | 2.378 | 1.423 | 1.323 | 1.121 | 2.544 | 0.000  /0.001 | 1.289 | 0.042  /0.121 | 0.023 | Up-regulated by osmotic stress with ABA-dependent way |
| *C0PAY9 | snare-interacting protein keule | gsDDGYssDSVLk | S(2): 99.9 | 29 | 2.311 | 1.888 | 1.577 | 1.231 | 0.963 | 0.899 | 1.925 | 0.006  /0.013 | 1.031 | 0.720  /0.863 | 0.017 | Up-regulated by osmotic stress with ABA-dependent way |
| *C0PB33 | transcription elongation factor spt5-like | aATPAQGEDEGIEDGEEAAVLsPR | S(22): 100.0 | 90 | 1.722 | 1.790 | 1.687 | 1.367 | 1.211 | 1.013 | 1.733 | 0.022  /0.036 | 1.197 | 0.541  /0.748 | 0.030 | Up-regulated by osmotic stress with ABA-dependent way |
| *C0PBN0 | transcription factor vip1-like | sGsmDGAtSPFEGESAPTSVLPDYAk | S(3): 85.0 | 30 | 2.324 | 1.957 | 1.655 | 1.247 | 0.633 | 0.998 | 1.979 | 0.004  /0.010 | 0.959 | 0.337  /0.572 | 0.035 | Up-regulated by osmotic stress with ABA-dependent way |
| C0PEW7 | vacuolar amino acid transporter 1-like | lSNsFLAITDsFR | S(11): 94.9 | 29 | 1.653 | 1.553 | 1.653 | 1.041 | 1.141 | 1.241 | 1.620 | 0.041  ~~/0.056~~ | 1.141 | 0.357  /0.592 | 0.019 | Up-regulated by osmotic stress with ABA-dependent way |
| *C0PG75 | ankyrin repeat domain-containing protein 13b-like | vSDLVIEsPSNVQTRPGR | S(8): 99.9 | 13 | 0.563 | 0.664 | 0.650 | 0.899 | 1.322 | 1.112 | 0.626 | 0.039  ~~/0.054~~ | 1.111 | 0.458  /0.683 | 0.035 | Down-regulated by osmotic stress with ABA-dependent way |
| C0PGM9 | pentatricopeptide repeat-containing protein at5g27270-like | rRFVPEEAtysLLIsASsR | T(9):100.0; Y(10):99.9; S(11):99.9; S(15): 95.8 | 19 | 0.278 | 0.473 | 0.332 | 0.780 | 0.881 | 1.023 | 0.361 | 0.000  /0.000 | 0.895 | 0.112  /0.274 | 0.023 | Down-regulated by osmotic stress with ABA-dependent way |
| *C0PH11 | wpp domain-interacting protein 2-like isoform x1 | sSFVPLEVsPPPPPTLTR | S(9): 100.0 | 26 | 0.439 | 0.389 | 0.590 | 0.878 | 0.877 | 1.147 | 0.473 | 0.001  /0.003 | 0.967 | 0.527  /0.737 | 0.005 | Down-regulated by osmotic stress with ABA-dependent way |
| *C0PHF7 | fas-associated factor 2-b | dSNTQtGSSGQGPIIEDVTGR | T(6):82.1 | 15 | 1.562 | 2.181 | 1.851 | 0.899 | 1.211 | 1.037 | 1.865 | 0.007  /0.016 | 1.049 | 0.811  /0.932 | 0.012 | Up-regulated by osmotic stress with ABA-dependent way |
| *C0PJF1 | basic proline-rich | sPSQQPPR | S(1): 100.0 | 15 | 1.765 | 1.646 | 1.523 | 0.651 | 0.745 | 0.867 | 1.645 | 0.032  /0.046 | 0.754 | 0.023  /0.072 | 0.021 | Up-regulated by osmotic stress with ABA-dependent way |
|  |  | rPPsPPAPAPPAAEELTEAGTEER | S(4): 100.0 | 38 | 1.534 | 1.926 | 1.727 | 0.878 | 1.045 | 1.008 | 1.729 | 0.018  /0.031 | 0.977 | 0.956  /0.982 | 0.008 | Up-regulated by osmotic stress with ABA-dependent way |
| C0PJV8 | actin-related protein 5-like | mSkDNDDDsNDDDESELVR | S(9): 99.2 | 20 | 0.299 | 0.491 | 0.479 | 0.890 | 1.024 | 1.234 | 0.423 | 0.000  /0.001 | 1.049 | 0.929  /0.982 | 0.011 | Down-regulated by osmotic stress with ABA-dependent way |
| *C0PKH3 | serine threonine-protein kinase afc3-like | gGAsPPWR | S(4): 100.0 | 20 | 0.590 | 0.626 | 0.665 | 1.130 | 0.978 | 1.133 | 0.627 | 0.039  /0.054 | 1.080 | 0.386  /0.611 | 0.014 | Down-regulated by osmotic stress with ABA-dependent way |
| C0PKN2 | phosphoglycerate dehydrogenase | gLVEPVsSTFVNLVNADYtAk | T(19): 50.0 | 11 | 2.234 | 2.940 | 2.592 | 0.877 | 1.210 | 1.045 | 2.589 | 0.000  /0.000 | 1.044 | 1.000  /1.001 | 0.005 | Up-regulated by osmotic stress with ABA-dependent way |
| *C0PL59 | eukaryotic translation initiation factor 3 subunit 1 35kda | qQALTsAEEPDEPPLsPTSQk | S(6): 95.8 | 18 | 0.586 | 0.455 | 0.627 | 0.943 | 1.118 | 1.206 | 0.556 | 0.008  /0.017 | 1.089 | 0.868  /0.963 | 0.028 | Down-regulated by osmotic stress with ABA-dependent way |
| *C0PLA9 | nodulin-like protein | eEVTEDSENASSSTTALGGsNQDLSSGk | S(20): 99.9 | 25 | 1.992 | 1.592 | 1.512 | 0.880 | 0.486 | 0.987 | 1.699 | 0.042  /0.037 | 0.784 | 0.000  /0.179 | 0.042 | Up-regulated by osmotic stress with ABA-dependent way |
| C0PLQ0 | hypothetical protein | sSsVIsVAPSASELAAGmGREDGR | S(1): 75.5 | 22 | 2.244 | 2.367 | 1.932 | 0.951 | 1.001 | 1.211 | 2.181 | 0.001  /0.003 | 1.054 | 0.943  /0.981 | 0.031 | Up-regulated by osmotic stress with ABA-dependent way |
| *C0PLZ2 | probable peptide nitrate transporter at5g13400-like | tPLGAAYEPPSAAAGGGGTtPVNIR | T(20): 95.8 | 36 | 0.522 | 0.526 | 0.580 | 1.392 | 0.823 | 1.129 | 0.543 | 0.043  /0.058 | 1.115 | 0.400  /0.628 | 0.075 | Down-regulated by osmotic stress with ABA-dependent way |
| *C0PM56 | chloroplast post-illumination chlorophyll fluorescence increase protein | lDIVSGcTDPSSDmFDPLATVDDGScPLEsDSEE | S(30):50.0; S(32): 50.0 | 45 | 1.522 | 1.645 | 1.864 | 0.871 | 0.815 | 1.466 | 1.677 | 0.039  /0.054 | 1.051 | 0.179  /0.380 | 0.038 | Up-regulated by osmotic stress with ABA-dependent way |
|  |  | lDIVSGcTDPSSDMFDPLATVDDGScPLESDsEE | S(32): 80.0 | 44 | 2.158 | 1.488 | 1.640 | 1.517 | 0.428 | 1.018 | 1.762 | 0.018  /0.031 | 0.988 | 0.472  /0.686 | 0.032 | Up-regulated by osmotic stress with ABA-dependent way |
| *C0PMQ0 | pre-mrna-splicing factor syf1-like | qQAGNNEDIELPDEsDDEEPDVQIAEk | S(15): 100.0 | 54 | 1.660 | 1.758 | 1.899 | 1.131 | 0.916 | 1.112 | 1.772 | 0.016  /0.029 | 1.053 | 0.940  /0.981 | 0.018 | Up-regulated by osmotic stress with ABA-dependent way |
| *C0PNN7 | atp synthase gamma chain chloroplast (h(+)-transporting two-sector atpase f -atpase atpc1) | nLsIAYNR | S(3): 100.0 | 35 | 0.659 | 0.537 | 0.659 | 0.880 | 0.929 | 1.001 | 0.618 | 0.032  /0.045 | 0.936 | 0.407  /0.634 | 0.024 | Down-regulated by osmotic stress with ABA-dependent way |
| *C0PPH0 | unknown | eGtGAGSsAR | S(8): 97.2 | 15 | 0.604 | 0.456 | 0.654 | 0.981 | 0.978 | 1.234 | 0.571 | 0.013  /0.025 | 1.064 | 0.840  /0.950 | 0.015 | Down-regulated by osmotic stress with ABA-dependent way |
| C4IYD7 | c-type lectin receptor-like tyrosine-protein kinase at1g52310-like isoform x1 | sGtsTSATsPmLPLEVRtPR | S(9):87.8; T(18): 87.8 | 15 | 0.292 | 0.452 | 0.504 | 1.011 | 0.876 | 0.977 | 0.416 | 0.000  /0.000 | 0.955 | 0.443  /0.672 | 0.028 | Down-regulated by osmotic stress with ABA-dependent way |
| C4J0D7 | protein smg7-like | rFDSVsSNVGSLQSk | S(6):78.9 | 14 | 1.712 | 1.568 | 1.583 | 0.898 | 1.013 | 1.221 | 1.621 | 0.041  ~~/0.056~~ | 1.008 | 0.972  /0.987 | 0.001 | Up-regulated by osmotic stress with ABA-dependent way |
| C4J0E7 | pyrrolidone-carboxylate peptidase | hVNSLcHYIFLVsLHsRGR | S(13):99.4; S(16): 100.0 | 15 | 2.467 | 1.965 | 2.230 | 0.889 | 0.988 | 1.121 | 2.221 | 0.001  /0.003 | 0.999 | 0.696  /0.863 | 0.022 | Up-regulated by osmotic stress with ABA-dependent way |
| C4J107 | tpa: protein kinase domain superfamily protein | sQsVsPR | S(3):100.0;S(5): 100.0 | 13 | 2.023 | 1.534 | 1.748 | 1.222 | 1.012 | 0.968 | 1.768 | 0.015  /0.027 | 1.067 | 0.783  /0.918 | 0.016 | Up-regulated by osmotic stress with ABA-dependent way |
| *C4J1U3 | adp-ribosylation factor gtpase-activating protein agd3-like | aLQQEQPVsPPDGsPk | S(9):100.0; S(14): 100.0 | 30 | 2.286 | 2.333 | 2.652 | 1.429 | 1.253 | 1.253 | 2.424 | 0.000  /0.001 | 1.312 | 0.289  /0.509 | 0.019 | Up-regulated by osmotic stress with ABA-dependent way |
| *C4J2P1 | protein kinase superfamily protein | asPEPGEVSGGR | S(2): 100.0 | 12 | 1.990 | 1.501 | 1.695 | 1.210 | 0.899 | 0.867 | 1.729 | 0.025  /0.039 | 0.992 | 0.268  /0.484 | 0.009 | Up-regulated by osmotic stress with ABA-dependent way |
|  |  | sVsPADSSVPGQWk | S(1):100.0; S(3): 100.0 | 15 | 0.389 | 0.521 | 0.539 | 0.899 | 1.231 | 1.183 | 0.483 | 0.001  /0.004 | 1.104 | 0.239  /0.444 | 0.009 | Down-regulated by osmotic stress with ABA-dependent way |
| *C4J2S2 | unc93-like protein 3-like isoform x1 | aGADAGLASAAGDVEEAAPLVAGPGsR | S(26): 100.0 | 20 | 3.200 | 2.452 | 1.989 | 0.987 | 1.009 | 1.001 | 2.547 | 0.000  /0.000 | 0.999 | 1.000  /1.001 | 0.049 | Up-regulated by osmotic stress with ABA-dependent way |
| C4J2X3 | methylcytosine binding domain protein | sSIIAQcFGtTSNDDTAck | T(10):32.7; T(11): 32.7; S(12): 32.7 | 10 | 0.197 | 0.382 | 0.545 | 0.846 | 1.140 | 1.260 | 0.375 | 0.000  /0.000 | 1.082 | 0.380  /0.604 | 0.002 | Down-regulated by osmotic stress with ABA-dependent way |
| *C4J747 | ninja-family protein 8 | hASADAPAQDNsATLPAFPAGNQAtSAEN | S(12):79.8; | 26 | 1.756 | 1.745 | 1.933 | 0.791 | 0.840 | 0.867 | 1.811 | 0.000  /0.002 | 0.833 | 0.171  /0.364 | 0.002 | Up-regulated by osmotic stress with ABA-dependent way |
| *C4J9N7 | uncharacterized protein LOC100502251 | qVsNAk | S(3): 100.0 | 22 | 0.586 | 0.516 | 0.565 | 0.879 | 0.793 | 0.878 | 0.556 | 0.011  /0.022 | 0.850 | 0.309  /0.534 | 0.001 | Down-regulated by osmotic stress with ABA-dependent way |
| *C4J9U3 | transmembrane protein 184c-like isoform x2 | vIsGIDDPLLNGSLSDNSGPk | S(3): 100.0 | 25 | 0.391 | 0.517 | 0.525 | 0.899 | 1.231 | 1.183 | 0.478 | 0.001  /0.004 | 1.104 | 0.239  /0.444 | 0.009 | Down-regulated by osmotic stress with ABA-dependent way |
| C4JC53 | pur alpha-1 | sTIIVPAGsSGEEGWEAFR | S(9): 80.0; | 15 | 0.389 | 0.503 | 0.474 | 0.698 | 0.910 | 0.788 | 0.455 | 0.000  /0.002 | 0.799 | 0.070  /0.189 | 0.009 | Down-regulated by osmotic stress with ABA-dependent way |
| *E1U817 | HRGP | dLGDNNLPsPTR | S(9): 100.0 | 54 | 0.336 | 0.602 | 0.470 | 1.133 | 1.243 | 1.246 | 0.469 | 0.001  /0.003 | 1.207 | 0.130  /0.302 | 0.004 | Down-regulated by osmotic stress with ABA-dependent way |
| *E9JVD2 | aldose reductase | gTNtSPENFVTPDFPATWGAmEk | T(4):78.9 | 16 | 0.196 | 0.286 | 0.252 | 0.788 | 0.946 | 1.103 | 0.245 | 0.000  /0.000 | 0.946 | 0.621  /0.808 | 0.012 | Down-regulated by osmotic stress with ABA-dependent way |
| *G2J5R7 | tpa_exp: homeodomain leucine zipper family iv protein | sGSDNVDGAsGDEIDPDNSNPR | S(1): 93.7; S(10): 99.9 | 28 | 0.466 | 0.387 | 0.523 | 0.969 | 0.812 | 0.671 | 0.459 | 0.000  /0.002 | 0.817 | 0.097  /0.249 | 0.080 | Down-regulated by osmotic stress with ABA-independent way |
| *K7TFK8 | e3 ubiquitin-protein ligase upl1-like | dVsNAsELATEMQYER | S(3):100.0;S(6): 100.0 | 33 | 2.132 | 1.929 | 1.958 | 1.153 | 1.099 | 0.900 | 2.007 | 0.009  /0.019 | 1.051 | 0.473  /0.683 | 0.005 | Up-regulated by osmotic stress with ABA-dependent way |
|  |  | lRPGQPDAVQDAStSDmEDASTSSGGQR | T(14):79.2 | 26 | 1.656 | 1.626 | 1.701 | 1.420 | 1.766 | 1.087 | 1.661 | 0.029  /0.043 | 1.424 | 0.020  /0.065 | 0.390 | Up-regulated by osmotic stress with ABA-independent way |
| *K7TL05 | 14-3-3-like protein gf14-12 | hDsSEGQ | S(3): 99.0 | 32 | 1.406 | 1.591 | 1.508 | 0.918 | 0.973 | 0.984 | 1.502 | 0.047  ~~/0.060~~ | 0.958 | 0.648  /0.824 | 0.053 | Up-regulated by osmotic stress with ABA-independent way |
| *K7TRF3 | at-hook protein 1 | tAAAGVTEAtGAQSGGGGSTPNPDGk | T(10): 95.3 | 33 | 0.531 | 0.557 | 0.377 | 0.966 | 0.888 | 0.945 | 0.488 | 0.001  /0.004 | 0.933 | 0.517  /0.727 | 0.023 | Down-regulated by osmotic stress with ABA-dependent way |
| K7TS17 | tpa: crinkly4-like receptor protein kinase family protein | nIVcLLGccADsGER | S(12): 100.0 | 12 | 2.530 | 1.927 | 1.678 | 1.110 | 1.244 | 1.321 | 2.045 | 0.003  /0.008 | 1.225 | 0.121  /0.288 | 0.121 | Up-regulated by osmotic stress with ABA-dependent way |
| *K7TTT8 | pre-mrna-splicing factor slu7-like | dTEkDDENAGsEDEEDDLR | S(11): 100.0 | 30 | 0.493 | 0.635 | 0.665 | 1.001 | 1.246 | 1.423 | 0.597 | 0.024  /0.038 | 1.223 | 0.127  /0.298 | 0.013 | Down-regulated by osmotic stress with ABA-dependent way |
| K7TV96 | homeobox dna-binding domain superfamily protein | gGPGsPLPLkLE | S(5): 100.0 | 25 | 0.496 | 0.546 | 0.628 | 0.945 | 0.808 | 1.106 | 0.557 | 0.009  /0.019 | 0.953 | 0.632  /0.812 | 0.028 | Down-regulated by osmotic stress with ABA-dependent way |
| *K7TWA4 | regulatory-associated protein of tor 1-like | ssSWLDmNSGNNLIk | S(2):79.3 | 27 | 0.645 | 0.567 | 0.690 | 0.864 | 1.211 | 1.014 | 0.634 | 0.047  ~~/0.0.60~~ | 1.030 | 0.950  /0.979 | 0.090 | Down-regulated by osmotic stress with ABA-independent way |
| K7TWL5 | casein kinase i | mAtEsDsDSDAR | T(3): 98.6 | 12 | 0.391 | 0.491 | 0.447 | 1.291 | 1.127 | 0.902 | 0.443 | 0.000  /0.001 | 1.106 | 0.525  /0.737 | 0.004 | Down-regulated by osmotic stress with ABA-dependent way |
| *K7TX27 | myosin heavy chain homolog-like protein | kPDtASDPGGEEDSEAR | T(4): 96.9 | 24 | 0.535 | 0.456 | 0.624 | 1.231 | 1.003 | 1.432 | 0.538 | 0.005  /0.012 | 1.222 | 0.143  /0.320 | 0.012 | Down-regulated by osmotic stress with ABA-dependent way |
| *K7TXK2 | far upstream element-binding protein 1-like | gVsPQPGGGAAAYGAGGQWTA | S(3): 100.0 | 89 | 0.585 | 0.640 | 0.842 | 0.919 | 0.931 | 0.909 | 0.689 | 0.046  ~~/0.060~~ | 0.919 | 0.472  /0.686 | 0.108 | Down-regulated by osmotic stress with ABA-independent way |
| K7TXP1 | protein artemis-like | aSDsstVVGsskGLNASLR | S(4): 89.8; S(5): 89.8; T(6): 89.8; S(10):87.5; S(11): 87.5 | 13 | 1.988 | 2.155 | 2.001 | 1.134 | 0.965 | 0.974 | 2.048 | 0.002  /0.007 | 1.024 | 0.822  /0.944 | 0.009 | Up-regulated by osmotic stress with ABA-dependent way |
| *K7TZ83 | sucrose-phosphate synthase family protein | eAAEELsDGEkDGAPDAAQQPVSVAAPDGR | S(7): 100.0 | 34 | 0.576 | 0.420 | 0.368 | 1.045 | 0.924 | 1.230 | 0.455 | 0.000  /0.002 | 1.067 | 0.034  /0.104 | 0.040 | Down-regulated by osmotic stress with ABA-dependent way |
| *K7U0Y3 | dag protein | qQAPAQTQTESASs | S(14): 50.0 | 69 | 0.281 | 0.189 | 0.168 | 1.105 | 0.940 | 0.937 | 0.213 | 0.000  /0.000 | 0.994 | 0.584  /0.777 | 0.001 | Down-regulated by osmotic stress with ABA-dependent way |
| *K7U162 | rna polymerase ii-associated factor 1 homolog | vEDIDQYSEEYsE | S(12): 99.9 | 36 | 1.043 | 0.285 | 0.453 | 0.663 | 0.915 | 1.162 | 0.594 | 0.000  /0.000 | 0.914 | 0.458  /0.679 | 0.458 | Down-regulated by osmotic stress with ABA-independent way |
| *K7U1F3 | transcription regulatory protein snf2 | sVENsADGVSNPTWtPDkGR | S(1):79.1 | 15 | 0.389 | 0.530 | 0.577 | 0.788 | 1.017 | 1.333 | 0.499 | 0.002  /0.006 | 1.046 | 0.956  /0.982 | 0.036 | Down-regulated by osmotic stress with ABA-dependent way |
| *K7U2M6 | heat shock protein sti | dVEPEPEAEPmDLtDEEk | T(14): 100.0 | 18 | 0.464 | 0.705 | 0.453 | 0.974 | 1.049 | 1.230 | 0.541 | 0.006  /0.014 | 1.084 | 0.989  /0.997 | 0.050 | Down-regulated by osmotic stress with ABA-dependent way |
|  |  | dVEPEPEAEPMDLtDEEk | T(14): 100.0 | 13 | 0.289 | 0.167 | 0.270 | 1.066 | 0.689 | 1.210 | 0.242 | 0.000  /0.000 | 0.988 | 0.686  /0.861 | 0.026 | Down-regulated by osmotic stress with ABA-dependent way |
| K7U2V8 | zinc transporter | sAsTPR | S(3): 97.7 | 15 | 1.733 | 1.930 | 1.833 | 0.945 | 1.113 | 1.045 | 1.832 | 0.007  /0.016 | 1.034 | 0.760  /0.901 | 0.000 | Up-regulated by osmotic stress with ABA-dependent way |
| *K7U4E0 | protein furry homolog isoform x1 | asEmDAVGLVFLsSADVQIR | S(2): 95.5 | 10 | 0.498 | 0.543 | 0.568 | 1.122 | 0.968 | 0.924 | 0.536 | 0.006  /0.013 | 1.005 | 0.532  /0.742 | 0.028 | Down-regulated by osmotic stress with ABA-dependent way |
|  |  | sGQLLPALItmSGPLSGVR | S(1):100.0; T(10): 88.1 | 15 | 0.484 | 0.654 | 0.605 | 1.560 | 1.232 | 1.337 | 0.581 | 0.015  /0.027 | 1.376 | 0.037  /0.112 | 0.033 | Down-regulated by osmotic stress with ABA-dependent way |
| *K7U4Z8 | neurofilament heavy polypeptide-like | qkQELtSGDEQTPDSSk | T(6):80.0 | 27 | 0.180 | 0.342 | 0.357 | 1.321 | 0.976 | 1.102 | 0.293 | 0.000  /0.000 | 1.133 | 0.512  /0.725 | 0.032 | Down-regulated by osmotic stress with ABA-dependent way |
| K7U5I6 | denn (aex-3) domain-containing | gFsSAPDLSGTYNPEVLTTQk | S(3):87.3 | 12 | 0.489 | 0.587 | 0.617 | 0.678 | 0.917 | 0.804 | 0.564 | 0.011  /0.022 | 0.800 | 0.095  /0.246 | 0.038 | Down-regulated by osmotic stress with ABA-dependent way |
| K7U6X8 | serine arginine repetitive matrix protein 2-like isoform x2 | qYRsPsADR | S(4): 99.9; S(6): 100.0 | 11 | 0.626 | 0.504 | 0.613 | 0.866 | 0.945 | 0.941 | 0.581 | 0.017  /0.030 | 0.917 | 0.616  /0.809 | 0.029 | Down-regulated by osmotic stress with ABA-dependent way |
| *K7U769 | repressor protein | sNGsDPENDESR | S(4): 100.0 | 31 | 0.456 | 0.478 | 0.826 | 1.230 | 0.971 | 0.904 | 0.587 | 0.016  /0.029 | 1.035 | 0.584  /0.777 | 0.157 | Down-regulated by osmotic stress with ABA-independent way |
| K7U7F5 | multidrug resistance | tVALVGGSGsGk | S(10): 94.1 | 11 | 1.785 | 2.011 | 1.567 | 0.976 | 0.978 | 1.111 | 1.788 | 0.012  /0.023 | 1.022 | 0.834  /0.953 | 0.045 | Up-regulated by osmotic stress with ABA-dependent way |
| *K7U7Q6 | nuclear matrix constituent protein 1-like | gEQsSEQPSER | S(4): 100.0 | 26 | 0.211 | 0.410 | 0.366 | 0.696 | 1.011 | 0.806 | 0.329 | 0.000  /0.000 | 0.838 | 0.097  /0.249 | 0.009 | Down-regulated by osmotic stress with ABA-dependent way |
| K7U808 | nucleic acid binding protein | ymAYsPSPSTTPHsPR | Y(1): 88.9; S(14): 99.9 | 16 | 0.598 | 0.564 | 0.652 | 1.050 | 0.870 | 0.942 | 0.605 | 0.031  /0.045 | 0.954 | 0.627  /0.809 | 0.021 | Down-regulated by osmotic stress with ABA-dependent way |
| K7U926 | stress enhanced protein chloroplastic-like isoform x2 | sLsIIR | S(3): 100.0 | 13 | 2.120 | 1.782 | 1.570 | 0.845 | 1.023 | 1.234 | 1.824 | 0.015  /0.028 | 1.034 | 0.934  /0.982 | 0.101 | Up-regulated by osmotic stress with ABA-dependent way |
| K7U9P2 | vacuolar protein sorting-associated protein 13a | lVLsmIEFFRsVssRINIGHLEk | S(4):100.0; S(11): 100.0;S(13): 100.0;S(14): 100.0 | 16 | 0.345 | 0.582 | 0.479 | 0.678 | 1.037 | 0.657 | 0.469 | 0.001  /0.003 | 0.791 | 0.083  /0.221 | 0.057 | Down-regulated by osmotic stress with ABA-independent way |
| *K7UAA9 | dna binding protein | pTEEGsDGGGGAGAGAGEk | S(6): 99.8 | 13 | 2.038 | 1.877 | 2.643 | 1.230 | 1.022 | 1.432 | 2.186 | 0.001  /0.003 | 1.228 | 0.137  /0.314 | 0.017 | Up-regulated by osmotic stress with ABA-dependent way |
| *K7UAH5 | pre-mrna-splicing factor cwc22 homolog | dSPsDVEDLT | S(4): 99.9 | 33 | 2.013 | 1.659 | 1.489 | 1.366 | 0.999 | 1.009 | 1.720 | 0.029  /0.043 | 1.125 | 0.838  /0.953 | 0.009 | Up-regulated by osmotic stress with ABA-dependent way |
| K7UAY1 | serine threonine-protein kinase ctr1 | tNVDPSIsIPGFVsSQIDNPTTTk | S(8): 93.9 | 28 | 0.298 | 0.390 | 0.407 | 1.434 | 0.883 | 1.223 | 0.365 | 0.000  /0.000 | 1.180 | 0.029  /0.090 | 0.048 | Down-regulated by osmotic stress with ABA-dependent way |
| *K7UBL3 | zinc finger c-x8-c-x5-c-x3-h type family protein | gANEEVsSINVDEDPNVPYERsPNAAIAk | S(7): 95.1; S(22): 100.0 | 35 | 0.409 | 0.627 | 0.570 | 0.925 | 1.328 | 1.624 | 0.535 | 0.002  /0.007 | 1.293 | 0.131  /0.303 | 0.041 | Down-regulated by osmotic stress with ABA-dependent way |
| *K7UBY5 | heterogeneous nuclear ribonucleoprotein r-like | qPsEEPEEQVDLEGDDDGmDDDDAGYR | S(3): 100.0 | 82 | 1.789 | 1.801 | 1.894 | 0.867 | 0.887 | 1.080 | 1.828 | 0.010  /0.021 | 0.945 | 0.845  /0.952 | 0.002 | Up-regulated by osmotic stress with ABA-dependent way |
|  |  | rGsRDDsEEPEEDDDNDER | S(3):100.0; S(7): 100.0 | 29 | 1.923 | 2.088 | 2.265 | 1.031 | 1.176 | 0.963 | 2.092 | 0.002  /0.005 | 1.057 | 0.880  /0.962 | 0.016 | Up-regulated by osmotic stress with ABA-dependent way |
|  |  | dDsEEPEEDDDNDER | S(3): 100.0 | 55 | 1.577 | 1.779 | 2.056 | 1.208 | 1.302 | 1.436 | 1.804 | 0.002  /0.014 | 1.315 | 0.880  /0.396 | 0.021 | Up-regulated by osmotic stress with ABA-dependent way |
| K7UCU2 | nitrilase-associated protein | sNNYk | S(1): 100.0 | 10 | 0.528 | 0.568 | 0.608 | 0.678 | 0.810 | 0.947 | 0.568 | 0.012  /0.023 | 0.812 | 0.112  /0.274 | 0.047 | Down-regulated by osmotic stress with ABA-dependent way |
| K7UDG0 | plant-specific domain tigr01627 family protein | ssPTHVR | S(1): 80.0; S(2): 80.0 | 11 | 0.585 | 0.634 | 0.496 | 1.000 | 0.991 | 1.130 | 0.572 | 0.015  /0.027 | 1.040 | 0.960  /0.983 | 0.031 | Down-regulated by osmotic stress with ABA-dependent way |
| K7UE59 | ring zinc finger domain superfamily protein | cMVsLsPPPPk | S(4): 100.0; S(6): 100.0 | 21 | 0.498 | 0.556 | 0.587 | 1.332 | 1.376 | 1.385 | 0.547 | 0.002  /0.007 | 1.364 | 0.020  /0.066 | 0.000 | Down-regulated by osmotic stress with ABA-dependent way |
| K7UHH6 | zinc finger c-x8-c-x5-c-x3-h type family protein | eQGsIGITANDDPyNGNEmSPSDQR | S(4): 100.0 | 15 | 0.245 | 0.360 | 0.495 | 0.808 | 0.904 | 1.007 | 0.367 | 0.000  /0.000 | 0.906 | 0.443  /0.672 | 0.001 | Down-regulated by osmotic stress with ABA-dependent way |
| *K7UJC3 | serine threonine-protein kinase at5g01020-like | fmDPGLEAQysPRAAEAAAk | Y(10):100.0; S(11): 100.0 | 16 | 0.198 | 0.321 | 0.330 | 1.222 | 0.892 | 1.187 | 0.283 | 0.000  /0.000 | 1.100 | 0.230  /0.433 | 0.025 | Down-regulated by osmotic stress with ABA-dependent way |
| *K7UKZ7 | transcription elongation factor spt6-like | eSEmDEHsGFsDGDGTGk | S(8):100.0; S(11): 99.7 | 23 | 0.648 | 0.596 | 0.653 | 1.300 | 1.101 | 1.321 | 0.632 | 0.042  ~~/0.057~~ | 1.241 | 0.063  /0.174 | 0.007 | Down-regulated by osmotic stress with ABA-dependent way |
| *K7ULJ1 | transcription factor voz1-like | aELSVPsPASSLQNSQGNNR | SS(7): 75.0 | 12 | 2.040 | 1.507 | 1.712 | 1.220 | 0.999 | 1.108 | 1.753 | 0.019  /0.032 | 1.109 | 0.473  /0.683 | 0.020 | Up-regulated by osmotic stress with ABA-dependent way |
| *K7UMX4 | solute carrier family facilitated glucose transporter member 8 | qSsLNAAGTssMAVLR | S(3):95.2; S(10): 95.4; S(11): 95.4 | 26 | 1.728 | 1.821 | 1.691 | 0.912 | 1.121 | 1.054 | 1.747 | 0.018  /0.031 | 1.029 | 0.701  /0.860 | 0.005 | Up-regulated by osmotic stress with ABA-dependent way |
| *K7UN43 | hypothetical protein ZEAMMB73_955518 | dGSAISTDQPNEDVEELNLmsPMR | S(21): 99.0 | 16 | 0.323 | 0.495 | 0.614 | 0.799 | 0.896 | 0.987 | 0.477 | 0.001  /0.003 | 0.894 | 0.375  /0.605 | 0.005 | Down-regulated by osmotic stress with ABA-dependent way |
| *K7UNQ6 | proline-rich receptor-like protein kinase perk2-like | aSsSSTSAADPNPNk | S(3): 89.7 | 14 | 0.389 | 0.297 | 0.423 | 1.040 | 0.899 | 1.212 | 0.370 | 0.000  /0.001 | 1.050 | 0.817  /0.943 | 0.007 | Down-regulated by osmotic stress with ABA-dependent way |
| K7US87 | hypothetical protein ZEAMMB73_913406 | sVtPPPQDDER | T(3): 90.8 | 15 | 2.204 | 1.870 | 2.644 | 1.264 | 1.211 | 1.430 | 2.239 | 0.000  /0.002 | 1.302 | 0.099  /0.251 | 0.028 | Up-regulated by osmotic stress with ABA-dependent way |
| *K7UT89 | jumonji-like transcription factor family protein | dTVAEDSAHATEEsGEENLQEk | T(11):80.0; S(14): 80.0 | 21 | 2.897 | 1.900 | 2.402 | 1.432 | 0.987 | 1.206 | 2.399 | 0.000  /0.001 | 1.208 | 0.186  /0.389 | 0.017 | Up-regulated by osmotic stress with ABA-dependent way |
|  |  | dTVAEDsAHAtEEsGEENLQEk | T(11):99.9; S(14): 100.0 | 25 | 2.187 | 3.704 | 2.880 | 0.977 | 1.498 | 1.244 | 2.924 | 0.000  /0.000 | 1.240 | 0.120  /0.287 | 0.028 | Up-regulated by osmotic stress with ABA-dependent way |
| *K7UTR3 | cell division cycle atpase-like | nASVSSDmsDLASQGSGNPVR | S(9): 75.0 | 41 | 3.070 | 2.562 | 2.438 | 1.320 | 1.291 | 1.189 | 2.690 | 0.002  /0.005 | 1.267 | 0.131  /0.303 | 0.013 | Up-regulated by osmotic stress with ABA-dependent way |
| K7UW53 | proline-rich receptor-like protein kinase perk1-like | fFGSYSSSDyDSGQYNEDmk | Y(10):84.5 | 16 | 2.260 | 2.011 | 1.922 | 1.070 | 1.230 | 0.964 | 2.064 | 0.003  /0.008 | 1.088 | 0.429  /0.657 | 0.014 | Up-regulated by osmotic stress with ABA-dependent way |
| *K7UWK7 | bah domain containing protein | sSISGsPAk | S(6): 97.8 | 13 | 0.398 | 0.547 | 0.517 | 1.088 | 0.878 | 0.983 | 0.487 | 0.001  /0.004 | 0.983 | 0.868  /0.963 | 0.042 | Down-regulated by osmotic stress with ABA-dependent way |
| *K7UYU6 | hypothetical protein ZEAMMB73_652457 | sAPLLAsSSSSR | S(7): 96.8 | 33 | 1.844 | 2.444 | 1.655 | 1.015 | 1.222 | 0.988 | 1.981 | 0.003  /0.009 | 1.075 | 0.929  /0.982 | 0.032 | Up-regulated by osmotic stress with ABA-dependent way |
| *K7UZK2 | zinc finger ccch domain-containing protein 44-like | nVDVDsDGER | S(6): 100.0 | 29 | 2.282 | 1.968 | 2.223 | 1.365 | 1.063 | 1.211 | 2.158 | 0.001  /0.002 | 1.213 | 0.192  /0.391 | 0.001 | Up-regulated by osmotic stress with ABA-dependent way |
| K7V021 | transcriptional regulator superman-like | mEsGAsGNmESk | S(3): 100.0; S(6): 100.0 | 25 | 0.446 | 0.578 | 0.562 | 1.111 | 0.999 | 1.093 | 0.529 | 0.005  /0.011 | 1.068 | 0.541  /0.748 | 0.017 | Down-regulated by osmotic stress with ABA-dependent way |
| *K7V0H3 | protein hasty 1-like | vSGDNSAAGNSAStGDLSTEk | T(14): 79.9 | 54 | 2.024 | 1.852 | 1.537 | 1.083 | 1.200 | 1.064 | 1.804 | 0.011  /0.022 | 1.116 | 0.634  /0.812 | 0.037 | Up-regulated by osmotic stress with ABA-dependent way |
| K7V0J1 | protein ric1 homolog | qSsDLk | S(3): 100.0 | 23 | 0.489 | 0.657 | 0.644 | 0.671 | 0.931 | 0.764 | 0.597 | 0.021  /0.035 | 0.789 | 0.041  /0.121 | 0.050 | Down-regulated by osmotic stress with ABA-dependent way |
| K7V0U5 | myb dna-binding domain superfamily protein | mAHLQNEEGyHk | Y(10): 100.0 | 13 | 0.609 | 0.556 | 0.662 | 0.865 | 0.767 | 0.961 | 0.609 | 0.027  /0.042 | 0.864 | 0.265  /0.481 | 0.010 | Down-regulated by osmotic stress with ABA-dependent way |
| K7V1I2 | arginine serine-rich splicing factor sr45_2 transcript i | gGtPPR | T(3): 100.0 | 18 | 0.310 | 0.308 | 0.387 | 0.859 | 0.949 | 0.827 | 0.335 | 0.000  /0.000 | 0.878 | 0.234  /0437 | 0.011 | Down-regulated by osmotic stress with ABA-dependent way |
| *K7V3F9 | phd finger protein | vAVPPkDDDDEsGEEYEEEER | S(12): 99.9 | 17 | 1.905 | 1.459 | 1.646 | 1.139 | 1.292 | 1.256 | 1.670 | 0.031  /0.045 | 1.229 | 0.103  /0.256 | 0.128 | Up-regulated by osmotic stress with ABA-independent way |
| K7V3T9 | hypothetical protein ZEAMMB73_910657 | aQLsPsAEVQDsGGSSPHNQSSSk | S(4): 75.4 | 13 | 0.325 | 0.435 | 0.217 | 0.877 | 0.988 | 1.213 | 0.326 | 0.000  /0.000 | 1.026 | 0.983  /0.944 | 0.042 | Down-regulated by osmotic stress with ABA-dependent way |
| *K7V4D9 | e3 ubiquitin-protein ligase upl4-like | aDsPSEGLTcGSQNLPAETcPk | S(3): 99.8 | 23 | 2.888 | 2.521 | 2.666 | 1.010 | 0.987 | 0.949 | 2.692 | 0.000  /0.000 | 0.982 | 0.665  /0.841 | 0.003 | Up-regulated by osmotic stress with ABA-dependent way |
| K7V6A5 | peroxin pex14 | sSFAPAPAEPtAGSFSR | T(11): 95.1 | 18 | 0.493 | 0.512 | 0.458 | 1.023 | 0.877 | 0.943 | 0.488 | 0.001  /0.004 | 0.948 | 0.627  /0.809 | 0.011 | Down-regulated by osmotic stress with ABA-dependent way |
| *K7V792 | splicing factor 3b subunit 1-like isoform x1 | mADADAtPAAGGATPGATPSGAWDAtPk | T(7): 96.0 | 21 | 1.487 | 2.023 | 1.819 | 0.769 | 0.967 | 0.671 | 1.776 | 0.021  /0.034 | 0.802 | 0.190  /0.391 | 0.018 | Up-regulated by osmotic stress with ABA-dependent way |
|  |  | lLAtPTPLGtPLYAIPEENR | T(10): 99.0 | 48 | 0.617 | 0.5270 | 0.473 | 0.969 | 0.939 | 0.919 | 0.537 | 0.042  /0.057 | 0.942 | 0.579  /0.787 | 0.015 | Down-regulated by osmotic stress with ABA-dependent way |
|  |  | mADADAtPAAGGAtPGAtPSGAWDAtPk | T(7):100.0; T(14): 100.0;T(18): 99.9; T(26): 96.9 | 22 | 0.278 | 0.321 | 0.359 | 0.999 | 1.210 | 1.171 | 0.319 | 0.000  /0.000 | 1.127 | 0.271  /0.487 | 0.004 | Down-regulated by osmotic stress with ABA-dependent way |
| *K7V7L2 | o-acyltransferase wsd1-like | eSDLEAVGEPmsPAGR | S(12): 100.0 | 38 | 0.431 | 0.391 | 0.555 | 1.262 | 1.278 | 1.240 | 0.459 | 0.000  /0.002 | 1.260 | 0.114  /0.275 | 0.006 | Down-regulated by osmotic stress with ABA-dependent way |
| *K7V9B4 | hypothetical protein ZEAMMB73_812027 | gGGRAAAGAsGQGR | S(10): 100.0 | 13 | 1.788 | 1.460 | 1.507 | 0.979 | 1.046 | 1.111 | 1.585 | 0.047  ~~/0.060~~ | 1.045 | 0.065  /0.178 | 0.027 | Up-regulated by osmotic stress with ABA-dependent way |
| *K7VAC7 | bzip transcription factor superfamily protein | rGPGSSSAGGDsQ | S(12): 100.0 | 40 | 1.959 | 1.642 | 1.497 | 1.380 | 1.488 | 0.855 | 1.699 | 0.023  /0.037 | 1.241 | 0.593  /0.786 | 0.096 | Up-regulated by osmotic stress with ABA-independent way |
| *K7VAI6 | lectin-like protein | mAAEDGEADSPAPAPVsPTGAAAASGGGTEk | S(17): 100.0 | 72 | 1.503 | 2.055 | 1.994 | 1.104 | 0.995 | 1.280 | 1.851 | 0.012  /0.023 | 1.126 | 0.303  /0.527 | 0.050 | Up-regulated by osmotic stress with ABA-dependent way |
| K7VAN4 | duf1421 domain family protein | sStsVNGTADDmLPSYDFQPIR | S(1):79.9 | 17 | 0.338 | 0.409 | 0.368 | 0.678 | 0.798 | 0.912 | 0.372 | 0.000  /0.000 | 0.796 | 0.079  /0.210 | 0.020 | Down-regulated by osmotic stress with ABA-dependent way |
| *K7VBC2 | vacuolar proton atpase a1-like | fLGTSEmDPDSEPDsAR | S(15): 100.0 | 34 | 2.080 | 1.857 | 1.646 | 1.012 | 0.903 | 1.030 | 1.861 | 0.007  /0.016 | 0.982 | 0.407  /0.634 | 0.023 | Up-regulated by osmotic stress with ABA-dependent way |
|  |  | fLGTSEMDPDSEPDsAR | S(15): 98.6 | 22 | 0.595 | 0.356 | 0.391 | 0.984 | 1.053 | 1.126 | 0.447 | 0.000  /0.001 | 1.054 | 0.745  /0.889 | 0.031 | Down-regulated by osmotic stress with ABA-dependent way |
| *K7VBH0 | phd-finger family expressed | aIEsDGAVDsDsDDTDGQVDk | S(4): 100.0 | 18 | 0.567 | 0.665 | 0.632 | 1.332 | 1.424 | 1.571 | 0.621 | 0.033  /0.047 | 1.442 | 0.012  /0.042 | 0.005 | Down-regulated by osmotic stress with ABA-dependent way |
| *K7VD89 | kh domain-containing protein at4g18375-like | nNtSmEPPGYEFDSNDGk | T(3):50.0 | 25 | 2.210 | 1.900 | 1.930 | 0.967 | 1.132 | 0.991 | 2.013 | 0.004  /0.010 | 1.030 | 0.920  /0.983 | 0.019 | Up-regulated by osmotic stress with ABA-dependent way |
| *K7VDG7 | duf1296 domain containing family protein | sPtTTTAIQSTIQSIk | S(1): 80.0 | 23 | 1.571 | 1.882 | 1.559 | 1.140 | 1.070 | 1.142 | 1.671 | 0.027  /0.041 | 1.117 | 0.351  /0.985 | 0.051 | Up-regulated by osmotic stress with ABA-dependent way |
| K7VDS0 | glucan endo- -beta-glucosidase 12-like | nPVQTscDFGGTAALTTADPSk | S(6): 83.6 | 13 | 0.398 | 0.516 | 0.582 | 0.899 | 1.161 | 1.433 | 0.499 | 0.002  /0.003 | 1.164 | 0.309  /0.535 | 0.022 | Down-regulated by osmotic stress with ABA-dependent way |
| K7VFF7 | peroxiredoxin-2e- chloroplastic-like | gFLGAPIRAVAssAsAAPAAAAk | S(12): 100.0; S(13): 100.0; S(15): 100.0 | 12 | 0.378 | 0.454 | 0.471 | 1.110 | 0.899 | 0.968 | 0.434 | 0.000  /0.001 | 0.992 | 0.777  /0.916 | 0.024 | Down-regulated by osmotic stress with ABA-dependent way |
| *K7VGC6 | cyclin-dependent kinase family protein | iPDLNLQDGPmVLsPPR | S(14): 100.0 | 40 | 1.550 | 1.722 | 1.521 | 1.101 | 1.161 | 0.878 | 1.597 | 0.046  ~~/0.060~~ | 1.047 | 0.326  /0.555 | 0.010 | Up-regulated by osmotic stress with ABA-dependent way |
| *K7VH66 | math domain-containing protein at5g43560-like isoform x2 | sMsEAAGR | S(3): 100.0 | 16 | 0.651 | 0.508 | 0.603 | 1.021 | 0.770 | 0.891 | 0.587 | 0.017  /0.029 | 0.894 | 0.371  /0.601 | 0.011 | Down-regulated by osmotic stress with ABA-dependent way |
|  |  | iDALEEGsSDGSDmsNR | S(8):80.0 | 22 | 2.230 | 2.669 | 3.356 | 1.001 | 1.167 | 1.330 | 2.752 | 0.000  /0.000 | 1.166 | 0.289  /0.509 | 0.021 | Up-regulated by osmotic stress with ABA-dependent way |
| K7VHJ5 | anthocyanidin -o-glucosyltransferase-like | qFLSDVVNVsPDR | S(10): 100.0 | 14 | 0.456 | 0.528 | 0.541 | 0.877 | 1.322 | 1.078 | 0.508 | 0.002  /0.007 | 1.092 | 0.611  /0.805 | 0.034 | Down-regulated by osmotic stress with ABA-dependent way |
| *K7VIK4 | protein early responsive to dehydration 15-like | lVSAsPAFR | S(5): 100.0 | 22 | 0.516 | 0.655 | 0.703 | 0.675 | 0.669 | 0.682 | 0.625 | 0.037  /0.050 | 0.675 | 0.004  /0.514 | 0.455 | Down-regulated by osmotic stress with ABA-independent way |
| K7VLM3 | epidermal growth factor receptor substrate 15-like 1-like isoform x1 | fDsIsSSkGEDVSGFDTGNSSR | S(3): 94.2 | 11 | 4.123 | 3.170 | 3.494 | 0.979 | 1.331 | 1.136 | 3.596 | 0.000  /0.000 | 1.149 | 0.370  /0.602 | 0.023 | Up-regulated by osmotic stress with ABA-dependent way |
| K7VLY6 | blue-light photoreceptor phr2-like | lNsAtYSVISPLPSSTPGLSR | S(3): 94.4 | 15 | 0.655 | 0.567 | 0.622 | 1.211 | 1.044 | 0.908 | 0.615 | 0.030  /0.044 | 1.054 | 0.822  /0.946 | 0.032 | Down-regulated by osmotic stress with ABA-dependent way |
| *K7VN66 | leucine-rich repeat receptor-like protein kinase family protein | gLTASGGDFTSsSk | S(11):48.4; S(12): 48.4 | 25 | 0.544 | 0.609 | 0.449 | 1.052 | 0.960 | 1.003 | 0.534 | 0.005  /0.012 | 1.005 | 0.972  //0.988 | 0.017 | Down-regulated by osmotic stress with ABA-dependent way |
| *K7VQB2 | loricrin-like isoform x1 | gsGDYGSGGGGFDR | S(2): 100.0 | 13 | 1.699 | 1.500 | 1.566 | 0.877 | 1.112 | 0.984 | 1.588 | 0.045  ~~/0.060~~ | 0.991 | 0.891  /0.962 | 0.041 | Up-regulated by osmotic stress with ABA-dependent way |
| *K7VQW5 | mediator of rna polymerase ii transcription subunit 12-like | eQsSPGPTAGTNQIk | S(3):80.0 | 23 | 1.911 | 2.309 | 1.705 | 0.787 | 0.835 | 0.872 | 1.942 | 0.025  /0.039 | 0.831 | 0.214  /0.420 | 0.025 | Up-regulated by osmotic stress with ABA-dependent way |
| K7VYU4 | gap sh3 binding protein | sSGsFNR | S(4): 100.0 | 19 | 0.539 | 0.649 | 0.732 | 0.912 | 0.823 | 0.831 | 0.640 | 0.046  ~~/0.060~~ | 0.855 | 0.138  /0.314 | 0.119 | Down-regulated by osmotic stress with ABA-independent way |
| *K7VZB7 | hua2-like protein 3-like isoform x1 | tNsFSNk | S(3): 100.0 | 30 | 0.588 | 0.498 | 0.569 | 0.888 | 1.030 | 0.778 | 0.552 | 0.008  /0.018 | 0.899 | 0.346  /0.583 | 0.069 | Down-regulated by osmotic stress with ABA-dependent way |
| *K7VZN2 | asf sf2-like pre-mrna splicing factor srp32 | sPNVsPANGEAAsPk | S(5):96.2; S(13): 100.0 | 25 | 2.560 | 2.171 | 1.990 | 1.278 | 1.273 | 1.303 | 2.240 | 0.000  /0.002 | 1.285 | 0.090  /0.233 | 0.032 | Up-regulated by osmotic stress with ABA-dependent way |
| *K7W1A6 | filament-like plant protein 4-like | dLQSAsPmTEsPSNk | S(6):95.7; S(11): 95.7 | 17 | 2.189 | 2.780 | 2.178 | 0.987 | 0.881 | 0.937 | 2.382 | 0.000  /0.001 | 0.935 | 0.439  /0.669 | 0.024 | Up-regulated by osmotic stress with ABA-dependent way |
| *K7W2F9 | sf16 protein | vAtPAAsMAGGR | T(3):100.0; S(7): 100.0 | 22 | 1.799 | 2.094 | 2.441 | 1.020 | 0.883 | 0.688 | 2.111 | 0.002  /0.005 | 0.864 | 0.317  /0.545 | 0.047 | Up-regulated by osmotic stress with ABA-dependent way |
| *K7W3J4 | dna cytosine methyltransferase zmet3 | tADDsDDDDTLEIWDDEDAGGR | S(5): 79.1 | 17 | 2.962 | 2.405 | 3.123 | 1.016 | 0.898 | 0.956 | 2.830 | 0.000  /0.000 | 0.957 | 0.692  /0.862 | 0.011 | Up-regulated by osmotic stress with ABA-dependent way |
| *K7W753 | probable aminotransferase acs10-like | nRPAPAPAPAPAPGsPPPAAASPILR | S(15): 100.0 | 11 | 2.755 | 2.381 | 2.280 | 1.270 | 1.251 | 1.330 | 2.472 | 0.000  /0.000 | 1.284 | 0.115  /0.862 | 0.017 | Up-regulated by osmotic stress with ABA-dependent way |
| *K7W829 | sister chromatid cohesion protein pds5 homolog b-like | lDGHTLsNEESDNDtLSVWk | S(7):77.6 | 22 | 0.399 | 0.552 | 0.517 | 1.021 | 0.994 | 1.058 | 0.489 | 0.001  /0.005 | 1.024 | 0.880  /0.962 | 0.009 | Down-regulated by osmotic stress with ABA-dependent way |
| *K7WE98 | low quality protein: protein scar2-like | qAVADDDDDDSWsE | S(13): 97.8 | 19 | 4.210 | 3.555 | 3.774 | 0.980 | 1.210 | 1.090 | 3.846 | 0.000  /0.000 | 1.093 | 0.558  /0.765 | 0.009 | Up-regulated by osmotic stress with ABA-dependent way |
| *M1GS93 | splicing arginine serine-rich 2 | aAcsGsP | S(4):100.0; S(6): 100.0 | 26 | 0.555 | 0.564 | 0.560 | 0.849 | 0.835 | 0.916 | 0.559 | 0.011  /0.022 | 0.867 | 0.199  /0.398 | 0.007 | Down-regulated by osmotic stress with ABA-dependent way |
| O04014 | tpa: 40s ribosomal protein s6 | asAAtSA | S(2):100.0; T(5): 100.0 | 29 | 2.017 | 1.865 | 1.947 | 0.992 | 1.254 | 0.913 | 1.943 | 0.005  /0.011 | 1.053 | 0.920  /0.983 | 0.024 | Up-regulated by osmotic stress with ABA-dependent way |
| *O48547 | nonphototropic hypocotyl protein expressed | vsEELR | S(2): 100.0 | 16 | 1.773 | 1.541 | 1.476 | 0.981 | 0.989 | 1.121 | 1.597 | 0.044  /0.058 | 1.030 | 0.358  /0.591 | 0.046 | Up-regulated by osmotic stress with ABA-dependent way |
|  |  | ssETGsR | S(2): 99.8; S(6): 99.9 | 14 | 2.123 | 2.050 | 1.541 | 1.112 | 0.861 | 1.052 | 1.905 | 0.005  /0.013 | 1.008 | 0.691  /0.862 | 0.051 | Up-regulated by osmotic stress with ABA-dependent way |
|  |  | eDPLLDsDDERPDsFDDDFR | S(7):100.0; S(14): 100.0 | 25 | 1.602 | 1.902 | 1.817 | 1.176 | 1.113 | 1.036 | 1.774 | 0.013  /0.025 | 1.109 | 0.380  /0.604 | 0.031 | Up-regulated by osmotic stress with ABA-dependent way |
| *P22275 | phosphoenolpyruvate carboxylase | dYEEVAAEGGsDDGDEEEEY | S(11):80.0 | 49 | 2.127 | 2.512 | 2.106 | 1.137 | 1.035 | 1.098 | 2.248 | 0.002  /0.006 | 1.090 | 0.740  /0.886 | 0.018 | Up-regulated by osmotic stress with ABA-dependent way |
| *P31927 | sucrose-phosphate synthase | gAGGGGGGGDPRsPTk | S(13): 99.8 | 32 | 0.679 | 0.511 | 0.618 | 1.209 | 1.059 | 0.953 | 0.603 | 0.035  /0.049 | 1.074 | 0.903  /0.970 | 0.020 | Down-regulated by osmotic stress with ABA-dependent way |
| P49106 | 14-3-3-like protein gf14-6 | rDsSEGQ | S(3): 100.0 | 25 | 2.650 | 2.292 | 2.121 | 1.012 | 0.958 | 1.203 | 2.354 | 0.000  /0.001 | 1.058 | 0.926  /0.984 | 0.025 | Up-regulated by osmotic stress with ABA-dependent way |
| *Q071L2 | almt1 | vLRDLAMAtRtmtVPsPVNITmATAVk | T(9): 98.9; T(11): 98.9; T(13): 98.9 | 24 | 0.637 | 0.612 | 0.631 | 0.934 | 1.110 | 0.999 | 0.627 | 0.039  ~~/0.054~~ | 1.014 | 0.573  /0.773 | 0.022 | Down-regulated by osmotic stress with ABA-dependent way |
| Q41802 | win1 precursor | mVsGGR | S(3): 100.0 | 16 | 0.533 | 0.615 | 0.705 | 0.702 | 0.677 | 0.705 | 0.618 | 0.032  /0.045 | 0.695 | 0.008  /0.026 | 0.259 | Down-regulated by osmotic stress with ABA-independent way |
| *Q6JN48 | ethylene-insensitive protein 2-like | sIVDSTPYVSDDGPPsLTFSR | S(16): 98.0 | 91 | 0.629 | 0.417 | 0.661 | 0.786 | 0.913 | 0.922 | 0.569 | 0.011  /0.022 | 0.874 | 0.258  /0.474 | 0.013 | Down-regulated by osmotic stress with ABA-dependent way |
|  |  | sYYDPsSVDGNENAGSPAYSk | S(6): 80.9 | 10 | 0.398 | 0.470 | 0.412 | 1.604 | 1.416 | 1.355 | 0.427 | 0.000  /0.001 | 1.458 | 0.010  /0.035 | 0.007 | Down-regulated by osmotic stress with ABA-dependent way |
| *Q6UNK5 | abc transporter b family member 1-like | nsVSsPIMTR | S(2):96.7;S(5): 96.6 | 25 | 2.429 | 2.169 | 2.175 | 1.294 | 1.330 | 1.378 | 2.258 | 0.001  /0.003 | 1.334 | 0.047  /0.135 | 0.013 | Up-regulated by osmotic stress with ABA-dependent way |
| Q7XBD9 | gag-pol precursor | tAFItPFGAFcYTSmsFGLk | S(16): 86.9 | 12 | 2.121 | 1.502 | 1.746 | 1.022 | 0.716 | 0.841 | 1.790 | 0.012  /0.023 | 0.860 | 0.196  /0.397 | 0.009 | Up-regulated by osmotic stress with ABA-dependent way |
| *Q8W149 | cell division cycle 5-like | eIQtPNPMAtPLAsPGPGItPR | T(4):100.0; T(10): 100.0;S(14): 100.0;T(20): 100.0 | 36 | 1.565 | 1.654 | 2.036 | 0.878 | 1.011 | 1.202 | 1.752 | 0.030  /0.044 | 1.030 | 0.568  /0.770 | 0.006 | Up-regulated by osmotic stress with ABA-dependent way |
|  |  | eIQtPNPMATPLAsPGPGITPR | T(4):100.0; S(14): 99.5 | 14 | 1.965 | 2.114 | 2.337 | 1.012 | 0.945 | 0.840 | 2.139 | 0.001  /0.004 | 0.932 | 0.621  /0.808 | 0.017 | Up-regulated by osmotic stress with ABA-dependent way |
| Q94FZ6 | af292500_1granule-bound starch synthase | tGFxMGRLSVDcNVVEPADVk | T(1):80.0 | 14 | 2.032 | 1.887 | 1.894 | 1.111 | 0.899 | 1.391 | 1.938 | 0.004  /0.011 | 1.134 | 0.279  /0.495 | 0.034 | Up-regulated by osmotic stress with ABA-dependent way |
| *Q94IQ9 | methyl- binding domain containing expressed | sDAEPAAAAAPVPGtNSDAAATDPAPGTk | T(15):78.8 | 42 | 4.463 | 4.189 | 5.820 | 1.130 | 1.004 | 1.432 | 4.824 | 0.000  /0.000 | 1.189 | 0.403  /0.632 | 0.011 | Up-regulated by osmotic stress with ABA-dependent way |
| *Q9ATM4 | aquaporin pip2-7 | aLGSFRsNA | S(7): 100 | 16 | 0.656 | 0.611 | 0.650 | 0.762 | 0.718 | 0.755 | 0.639 | 0.021  /0.035 | 0.745 | 0.028  /0.088 | 0.000 | Down-regulated by osmotic stress with ABA-dependent way |
|  |  | aLGsFR | S(4): 100.0 | 17 | 0.559 | 0.702 | 0.728 | 0.831 | 0.825 | 0.824 | 0.663 | 0.047  ~~/0.060~~ | 0.827 | 0.138  /0.314 | 0.096 | Down-regulated by osmotic stress with ABA-independent way |
|  |  | aLGsFRsNA | S(4): 100.0; S(7): 100.0 | 17 | 0.436 | 0.436 | 0.347 | 0.862 | 0.923 | 0.822 | 0.406 | 0.000  /0.000 | 0.869 | 0.153  /0.336 | 0.002 | Down-regulated by osmotic stress with ABA-dependent way |
| *Q9ATN2 | nod26-like membrane integral protein | mQsQLAADEFDTV | S(3): 100.0 | 45 | 2.286 | 2.147 | 1.851 | 1.296 | 1.377 | 1.297 | 2.094 | 0.000  /0.001 | 1.323 | 0.021  /0.069 | 0.026 | Up-regulated by osmotic stress with ABA-dependent way |
| *Q9FER8 | hmgi y protein | tEGAAsPSPSPAPAGDGSTPGkR | S(6): 97.1 | 42 | 0.362 | 0.330 | 0.385 | 1.003 | 0.933 | 0.856 | 0.359 | 0.000  /0.000 | 0.931 | 0.537  /0.747 | 0.008 | Down-regulated by osmotic stress with ABA-dependent way |
| *Q9FYS5 | hmgi y protein | tDGAASPSPSPAPAPAGDGSTPGk | S(6):82.6 | 23 | 3.760 | 4.398 | 4.646 | 0.868 | 1.011 | 0.947 | 4.268 | 0.000  /0.000 | 0.942 | 0.453  /0.678 | 0.005 | Up-regulated by osmotic stress with ABA-dependent way |
| *Q9XF58 | aquaporin pip2-4-like | lGsSAsFSR | S(3): 97.3 | 27 | 0.494 | 0.498 | 0.714 | 1.192 | 1.203 | 1.126 | 0.569 | 0.002  /0.006 | 1.174 | 0.221  /0.423 | 0.025 | Down-regulated by osmotic stress with ABA-dependent way |

**Note**: ^a, b^ Each value represents the average of three biological replicas. The average is significant at a p <0.05 and a false discovery rate (FDR) < 0.05 level. Moreover, these peptides whose UniProt ID are signed with * are also significant at FDR<0.01. FDR values attained by Benjamini-Hochberg method were shown in column and were used to adjust p-values (correction for multiple comparisons). These phosphopeptides whose FDR values were signed with delete line ‘’ were not significant. ‘-’, not measured. T-test is used to identify whether the difference is significant. A T-test value <0.05 is considered to be significant. OS = osmotic stress.

**Table S2**︱Proteins and their phosphoralation peptides with more than 1.5-fold changes only in maize mutant *vp5* under osmotic stress

| **UniProt ID** | **Protein name** | **Sequence of phosphorylati-on peptides** | **PhosphoRS-Site Probabilities**  **(>75%)** | **Ion score** | ***Vp5*: OS/control** | | | ***vp5*: OS/control** | | | ***Vp5*: OS/control** | | ***vp5*: OS/control** | | T-test | **Regulation of ABA and osmotic stress for peptides phosphosites** |
| --- | --- | --- | --- | --- | --- | --- | --- | --- | --- | --- | --- | --- | --- | --- | --- | --- |
|  |  |  |  |  | 1 | 2 | 3 | 1 | 2 | 3 | Average | **P-Value /FDR** | **Average** | **P-Value**  **/FDR** |  |  |
| *A0MBZ8 | gck-like kinase mik | fSSYEDMSNSGTVVQTQNEDPEtPR | T(23): 99.9 | 22 | — | — | — | 0.423 | 0.454 | 0.347 |  | 1.000  /1.035 | 0.408 | 0.000  /0.000 |  | Up-regulated by ABA |
| B4F808 | nucleic acid binding protein | eLALLNstLREDsPHPGsVsPFsNGGmkR | S(7):100.0;T(8):100.0;S(13): 100.0;S(18):100.0;S(20): 100.0;S(23): 100.0 | 29 | — | — | — | 0.415 | 0.526 | 0.289 |  | 1.000  /1.035 | 0.410 | 0.000 |  | Up-regulated by ABA |
|  |  | eLALLNstLREDSPHPGsVsPFsNGGmkR | S(7):76.5;T(8): 76.5;S(13):76.5; S(18):76.5;S(20): 97.0; S(23): 97.0; | 22 | — | — | — | 1.561 | 1.728 | 1.489 |  | 1.000  /1.035 | 1.593 | 0.007 |  | Down-regulated by ABA |
| *B4F9V2 | vesicle-associated protein 1-2-like | vTYVAPPQPPsPVPEEsEEGsPPR | S(11):100.0;S(17):100.0; S(21): 100.0 | 20 | 1.230 | 1.286 | 1.323 | 1.787 | 1.611 | 1.616 | 1.280 | 0.615  /0.700 | 1.671 | 0.001  /0.003 | 0.042 | Down-regulated by ABA |
| B4FBI8 | probable long-chain-alcohol o-fatty-acyltransferase 4-like | sVsLVScAVk | S(3):8 0.0 | 13 | 0.815 | 1.012 | 0.923 | 0.654 | 0.663 | 0.587 | 0.917 | 0.722  /0.808 | 0.635 | 0.001  /0.006 | 0.043 | Up-regulated by ABA |
| B4FCK4 | 40s ribosomal protein s9 | kAsGGGGDDEEEE | S(3): 100.0 | 19 | 0.987 | 1.082 | 1.020 | 1.560 | 1.487 | 1.550 | 1.030 | 0.643  /0.725 | 1.532 | 0.004  /0.016 | 0.010 | Down-regulated by ABA |
| *B4FDJ3 | epidermal growth factor receptor substrate 15-like 1-like isoform x2 | fDsFsSNADNGGNDTFAR | S(3):76.6 | 14 | 0.891 | 0.674 | 0.799 | 1.636 | 1.676 | 1.453 | 0.788 | 0.280  /0.333 | 1.588 | 0.002  /0.008 | 0.017 | Down-regulated by ABA |
| *B4FKD1 | nucleoporin nup53-like | eGSPMDGVVQyQQQSPTTPSGQQSQQQk | Y(11):20.0; S(15): 20.0; T(17): 20.0; T(18): 20.0; S(20): 20.0 | 29 | 1.110 | 1.492 | 1.298 | 1.913 | 2.084 | 1.872 | 1.300 | 0.251  /0.301 | 1.956 | 0.000  /0.000 | 0.012 | Down-regulated by ABA |
| B4FLR0 | protein grpe | vSAGPGPENsGDDDPTVVEDSVAPQkVEDVEEDGFDDGDAE | S(10): 93.8 | 25 | — | — | — | 2.566 | 2.763 | 3.371 |  | 1.000  /1.035 | 2.900 | 0.000  /0.000 |  | Down-regulated by ABA |
| *B4FMB8 | nudix hydrolase 24 | gDESDLsGYFR | S(7): 100.0 | 47 | 1.123 | 0.967 | 0.895 | 0.694 | 0.670 | 0.567 | 0.995 | 0.997  /1.071 | 0.644 | 0.003  /0.000 | 0.013 | Up-regulated by ABA |
| *B4FMI7 | wd repeat-containing protein 89 homolog | gRSsPVVsGSPSQNSDGSmSSWR | S(20): 83.9 | 17 | 1.221 | 1.262 | 1.276 | 1.776 | 1.660 | 1.676 | 1.253 | 0.321  /0.378 | 1.704 | 0.000  /0.002 | 0.013 | Down-regulated by ABA |
| *B4FPT5;B6SUQ3 | uncharacterized protein LOC100272657 | tLVQSLQEDDSSDNtAPR | T(15): 99.9 | 12 | 0.990 | 1.232 | 0.869 | 1.598 | 1.887 | 1.477 | 1.030 | 0.932  /1.014 | 1.654 | 0.001  /0.004 | 0.001 | Down-regulated by ABA |
| B4FQ59 | phosphoribulokinase precursor | lTsVFGGAAEPPk | T(2): 50.0; S(3): 50.0 | 12 | 1.098 | 0.798 | 1.044 | 1.788 | 1.512 | 1.432 | 0.980 | 0.858  /0.938 | 1.577 | 0.002  /0.008 | 0.029 | Down-regulated by ABA |
| *B4FRW3 | gbp17 type transcription partial | vQEPEAsDEDEDDEEEDIEEDEk | S(7): 100.0 | 45 | 1.332 | 1.375 | 1.305 | 2.520 | 2.109 | 1.912 | 1.337 | 0.280  /0.333 | 2.180 | 0.000  /0.000 | 0.041 | Down-regulated by ABA |
| B4FS09 | sodium hydrogen exchanger 6-like | nYLTPFFTsQTDDDNDDDFSQQPQNR | T(8): 24.6; S(9): 24.6; T(11): 24.6; S(20): 24.6 | 12 | — | — | — | 2.301 | 2.340 | 2.201 |  | 1.000  /1.035 | 2.281 | 0.000  /0.000 |  | Down-regulated by ABA |
| B4FTJ1 | sterile alpha motif domain-containing protein | mVLMItDGAAAVAGAGGLSGGGGARLssAk | T(6): 99.3 | 10 | 1.433 | 1.213 | 1.028 | 0.512 | 0.367 | 0.421 | 1.225 | 0.326  /0.383 | 0.433 | 0.000  /0.000 | 0.014 | Up-regulated by ABA |
| *B4FWI0 | 60s acidic ribosomal protein p0 | eEEkAPEPAEEsDEEMGFSLFDD | S(12): 96.2 | 12 | — | — | — | 1.752 | 1.687 | 1.681 |  | 1.000  /1.035 | 1.706 | 0.000  /0.002 |  | Down-regulated by ABA |
| *B4FX77 | zinc finger c-x8-c-x5-c-x3-h type family protein | eQGsIGItANEDPYNANEMSPSDQR | S(4): 100.0; T(8): 94.7 | 22 | 1.372 | 1.375 | 1.489 | 0.650 | 0.580 | 0.630 | 1.412 | 0.143  /0.175 | 0.620 | 0.001  /0.005 | 0.002 | Up-regulated by ABA |
| *B4FZ17 | dhhc-type zinc finger domain-containing protein | nTHPPEPESIDGINDtGVQTPQQFR | T(16): 49.0; T(20): 49.0 | 13 | — | — | — | 3.911 | 3.890 | 3.589 |  | 1.000  /1.035 | 3.797 | 0.000  /0.000 |  | Down-regulated by ABA |
| *B4FZG8 | GeBP transcription factor | eSAPsTANQSGDsQk | S(13): 99.8 | 14 | — | — | — | 2.819 | 3.450 | 2.656 |  | 1.000  /1.035 | 2.975 | 0.000  /0.000 |  | Down-regulated by ABA |
| B4FZM4 | cell division control protein 6 homolog | sPHAGtGTVcIPk | S(1): 33.3; T(6): 33.3; T(8): 33.3 | 16 | 0.712 | 0.648 | 0.696 | 0.567 | 0.654 | 0.650 | 0.685 | 0.055  /0.068 | 0.624 | 0.001  /0.005 | 0.298 | Down-regulated by osmotic stress with ABA-independent way |
| *B6SGQ1 | transposon protein | aTAPsPsSPTSPHVAPSALATNPAGAASPPLLEPLPLLR | S(5): 85.7 | 24 | 1.012 | 0.723 | 0.905 | 0.456 | 0.587 | 0.536 | 0.880 | 0.558  /0.637 | 0.526 | 0.000  /0.000 | 0.101 | Down-regulated by osmotic stress with ABA-independent way |
| B6SP24 | k(+) efflux antiporter 5-like | sSEGVFVGAFLSMSStAVVskFLVEk | S(15): 87.3; T(16): 87.3 | 17 | — | — | — | 0.454 | 0.436 | 0.424 |  | 1.000  /1.035 | 0.438 | 0.000  /0.000 |  | Up-regulated by ABA |
| *B6SSS0 | transposon protein mutator sub-class | wNLGGGGDskEsSPSR | S(9): 99.8; S(12): 99.8 | 19 | 0.965 | 1.041 | 0.983 | 1.734 | 2.045 | 1.787 | 0.996 | 0.979  /1.055 | 1.855 | 0.000  /0.000 | 0.007 | Down-regulated by ABA |
| B6SV26 | vacuolar amino acid transporter 1-like | kAssLQR | S(3): 100.0; S(4): 100.0 | 19 | 1.022 | 0.911 | 0.920 | 1.628 | 1.423 | 1.682 | 0.951 | 0.817  /0.899 | 1.578 | 0.002  /0.008 | 0.013 | Down-regulated by ABA |
| *B6SVF2 | gtp binding protein | asAEPLRFtVTPGDAFGDGPPVGmsEAAk | S(25): 99.7 | 12 | 1.012 | 1.402 | 1.327 | 0.298 | 0.421 | 0.374 | 1.247 | 0.346  /0.402 | 0.364 | 0.000  /0.000 | 0.009 | Up-regulated by ABA |
|  |  | asAEPLRFtVTPGDAFGDGPPVGMsEAAk | S(25): 92.9 | 17 | 1.228 | 1.122 | 1.432 | 0.664 | 0.598 | 0.675 | 1.261 | 0.329  /0.386 | 0.646 | 0.002  /0.008 | 0.013 | Up-regulated by ABA |
| B6SVI8 | protein lutein deficient chloroplastic-like | aATTPAmPAtGLssAGASPFR | S(18): 99.7 | 15 | — | — | — | 0.276 | 0.393 | 0.443 |  | 1.000  /1.035 | 0.371 | 0.000  /0.000 |  | Up-regulated by ABA |
| *B6SWM4 | bel1-related homeotic protein 30 | aPSPEENEDLQtPTSQAcQTSQLGESk | S(3): 48.1; T(12): 48.1 | 39 | 1.665 | 1.485 | 1.521 | 4.232 | 3.663 | 4.012 | 1.557 | 0.060  /0.074 | 3.969 | 0.000  /0.000 | 0.002 | Up-regulated by osmotic stress, but down-regulated by ABA |
| B6SWQ9 | dentin sialophospho | lFAQVsDSDEEVTLNNQADAQDGTTNSGTDR | S(6): 33.2; S(8): 33.2; T(13): 33.2 | 16 | — | — | — | 0.496 | 0.602 | 0.656 |  | 1.000  /1.035 | 0.585 | 0.000  /0.001 |  | Up-regulated by ABA |
| *B6SXR0 | uncharacterized protein LOC100275696 | vAPAsDAEDDDDDGGER | S(5): 100.0 | 37 | 1.151 | 1.054 | 0.955 | 1.550 | 1.502 | 1.571 | 1.053 | 0.858  /0.938 | 1.541 | 0.004  /0.014 | 0.018 | Down-regulated by ABA |
| *B6T2W8 | prp18 domain containing protein | vGGGDGGDGGAAGDsADDGDAkGsGDDADADkDSk | S(15): 100.0; S(24): 100.0 | 13 | 0.912 | 1.164 | 1.211 | 0.598 | 0.632 | 0.622 | 1.096 | 0.685  /0.770 | 0.617 | 0.000  /0.003 | 0.029 | Up-regulated by ABA |
| *B6T4N9 | nucleic acid binding protein | sSGDsGSSTPGHSDGDSSNR | S(5): 24.4; S(7): 24.4; S(8): 24.4; T(9): 24.4 | 14 | — | — | — | 2.417 | 1.987 | 3.133 |  | 1.000  /1.035 | 2.512 | 0.000  /0.000 |  | Down-regulated by ABA |
| *B6T6P7 | uba and ubx domain-containing protein | gPTGFPGAGGGDsGsDDDEPQEYYTGGEk | S(13): 99.9; S(15): 99.9 | 37 | 1.378 | 1.534 | 1.456 | 2.310 | 2.267 | 2.712 | 1.456 | 0.108  /0.132 | 2.430 | 0.000  /0.000 | 0.024 | Down-regulated by ABA |
| B6T927 | formin-like protein 7-like | eLASGstTSSPAPSPPPSTDSPPPAVDPDAFEk | S(4): 45.7; S(6): 45.7; T(7): 45.7 | 17 | 1.021 | 1.432 | 1.234 | 1.567 | 2.081 | 1.742 | 1.229 | 0.343  /0.399 | 1.797 | 0.000  /0.000 | 0.005 | Down-regulated by ABA |
| B6T9S5 | ferredoxin--nadp leaf isozyme | mAAVTAAAIsLsSSSASSxAAAAk | T(5): 42.3; S(10): 42.3; S(12): 42.3 | 16 | 0.912 | 0.863 | 0.893 | 1.770 | 1.498 | 1.506 | 0.889 | 0.596  /0.679 | 1.591 | 0.003  /0.212 | 0.012 | Down-regulated by ABA |
| B6TG72 | phd finger protein | vPQDEEESGDDDEDEEADEHNNtLcGTcGTNDSk | S(8): 32.2; T(23): 32.2; T(27): 32.2 | 10 | 1.320 | 1.344 | 1.462 | 0.431 | 0.660 | 0.778 | 1.375 | 0.162  /0.196 | 0.623 | 0.001  /0.003 | 0.008 | Up-regulated by ABA |
| *B6TJ15 | hypothetical protein | aAAVSDAASsPsSSSR | S(9): 33.3; S(10): 33.3; S(12): 33.3; S(13): 33.3; S(14): 33.3; S(15): 33.3 | 17 | 0.867 | 1.230 | 1.040 | 0.534 | 0.655 | 0.616 | 1.046 | 0.722  /0.808 | 0.602 | 0.000  /0.002 | 0.024 | Up-regulated by ABA |
| B6TZJ0 | hypothetical protein | aHtPtTPPk | T(3): 95.4; T(5): 95.4 | 20 | 0.900 | 1.121 | 1.032 | 2.120 | 1.567 | 1.598 | 1.018 | 0.685  /0.770 | 1.762 | 0.000  /0.001 | 0.091 | Down-regulated by ABA |
| B6U0R6 | tpa: duf827 domain containing family protein | nEAsVEDLQGMEDVSLSLEEYsELAAk | S(4): 40.0; S(15): 40.0; S(17): 40.0; Y(21): 40.0; S(22): 40.0 | 16 | — | — | — | 0.356 | 0.335 | 0.641 |  | 1.000  /1.035 | 0.444 | 0.000  /0.000 |  | Up-regulated by ABA |
| *B6U0Y9 | atp binding protein | aVQVSPILDGNQtDADSNTAGEEVASR | T(13): 96.4 | 52 | 1.391 | 1.690 | 1.557 | 1.624 | 1.600 | 1.558 | 1.546 | 0.060  /0.074 | 1.594 | 0.001  /0.006 | 0.667 | Up-regulated by osmotic stress with ABA-independent way |
| *B6U4I4 | microtubule-associated protein map65-1a | tASPGNNAVAAAPVDAVSQISVTDPVPStP | T(29): 97.7 | 28 | — | — | — | 1.790 | 1.426 | 2.110 |  | 1.000  /1.035 | 1.775 | 0.000  /0.001 |  | Down-regulated by ABA |
| *B6U5R0 | hypothetical protein | lPtVPPsAAAGDVsEVELSEAGSPDLGSR | T(3): 97.0 | 10 | — | — | — | 1.586 | 2.073 | 1.711 |  | 1.000  /1.035 | 1.790 | 0.005  /0.017 |  | Down-regulated by ABA |
| *B6UBN4 | j domain-containing protein required for chloroplast accumulation response 1-like isoform x2 | nDDGTSYAYsVPtSPNASmNNYLAQGAAR | S(10): 81.5 | 46 | — | — | — | 1.678 | 2.320 | 1.858 |  | 1.000  /1.035 | 1.952 | 0.000  /0.000 |  | Down-regulated by ABA |
| *B6UH65 | zinc transporter 2 precursor | sTGTAAtGGsDAGLEEGk | S(1): 48.4; T(2): 48.4; T(4): 48.4 | 10 | 0.686 | 0.645 | 0.665 | 2.999 | 3.120 | 2.973 | 0.665 | 0.069  /0.085 | 3.031 | 0.000  /0.000 | 0.001 | Down-regulated by ABA |
| B6UHC3 | probable calcium-binding protein cml11-like | gGSSVTETVTQAtQQQQsR | T(13): 41.7; S(18): 41.7 | 24 | — | — | — | 0.373 | 0.523 | 0.543 |  | 1.000  /1.035 | 0.480 | 0.000  /0.000 |  | Up-regulated by ABA |
| B6UI87 | uncharacterized protein LOC100279141 | vEDsSWDLQQkQWEsAAGGk | S(15): 100.0 | 10 | 1.112 | 1.434 | 1.453 | 1.776 | 2.121 | 1.798 | 1.333 | 0.218  /0.262 | 1.898 | 0.000  /0.000 | 0.036 | Down-regulated by ABA |
| B7ZYR5 | tpa: leucine-rich repeat receptor-like protein kinase family protein | atsEEERSGGtPPAAPtP | T(2): 98.7; T(17): 100.0 | 19 | 1.006 | 0.866 | 1.129 | 1.501 | 1.707 | 1.513 | 1.000 | 0.966  /1.045 | 1.574 | 0.003  /0.013 | 0.053 | Down-regulated by ABA |
| B8A3E3 | tpa: catalytic hydrolase | lFPAssVIAYNPALLsQEDEmLmAk | S(5): 75.0; S(6): 75.0; Y(10): 75.0; S(16): 75.0 | 12 | — | — | — | 0.625 | 0.567 | 0.651 |  | 1.000  /1.035 | 0.614 | 0.000  /0.002 |  | Up-regulated by ABA |
| *C0HF00 | vacuolar protein sorting-associated protein 41 homolog | sNSGQDsDGGMDDEDGSPSGQSR | S(7): 88.0 | 17 | 1.210 | 1.068 | 0.818 | 0.578 | 0.640 | 0.655 | 1.032 | 0.949  /1.029 | 0.624 | 0.003 | 0.095 | Up-regulated by ABA |
|  |  | sNSGQDsDGGmDDEDGSPSGQSR | S(1): 48.6; S(7): 48.6 | 14 | — | — | — | 2.131 | 1.531 | 1.762 |  | 1.000  /1.035 | 1.808 | 0.000  /0.000 |  | Down-regulated by ABA |
| *C0P5C4 | abc1 family protein | tsDADsEAGSGSGGGGR | S(2): 95.0 | 16 | 0.701 | 0.774 | 0.787 | 1.660 | 1.594 | 1.612 | 0.754 | 0.197  /0.237 | 1.622 | 0.001  /0.005 | 0.003 | Down-regulated by ABA |
| *C0P9I5 | tbc1 domain family member 15-like | sPDLYDLsDDSDYAAAASDQTAmR | S(8): 94.9 | 44 | 1.041 | 1.167 | 0.971 | 2.181 | 3.062 | 1.901 | 1.060 | 0.640  /0.724 | 2.381 | 0.000  /0.000 | 0.046 | Down-regulated by ABA |
| *C0PDG4 | unknown | tDsHGSAGVDGLETNESSLLSEPELQSTR | T(1): 66.6; S(3): 66.6; S(6): 66.6 | 27 | — | — | — | 1.645 | 2.561 | 1.905 |  | 1.000  /1.035 | 2.037 | 0.000  /0.000 |  | Down-regulated by ABA |
| *C0PHB9 | probable receptor-like protein kinase at5g56460-like | vSSTAkPEsPPkVQsPSEVDR | S(9): 100.0; S(17): 94.3 | 12 | 1.110 | 1.303 | 1.267 | 1.623 | 1.589 | 1.529 | 1.227 | 0.339  /0.396 | 1.580 | 0.002  /0.009 | 0.048 | Down-regulated by ABA |
| C4J5F6 | uncharacterized protein LOC100501718 | gPSGGsRAcTSAVSk | S(3): 25.0; S(6): 25.0; T(10): 25.0; S(11): 25.0 | 11 | 0.713 | 0.874 | 0.615 | 3.325 | 4.410 | 2.987 | 0.734 | 0.177  /0.214 | 3.574 | 0.000  /0.000 | 0.015 | Down-regulated by ABA |
| C4J5U4 | uncharacterized protein LOC100501774 | mLPPPPPPsPAsSAsASAPVGAR | S(9): 75.0; S(12): 75.0; S(13): 75.0; S(15): 75.0 | 14 | 0.891 | 1.023 | 0.912 | 1.880 | 2.330 | 1.719 | 0.942 | 0.791  /0.875 | 1.976 | 0.000  /0.000 | 0.019 | Down-regulated by ABA |
| C4J9M4 | myb dna-binding domain superfamily protein | eVmQVDGLTNDEVksHLQYR | T(9): 73.1 | 17 | 0.876 | 1.212 | 1.022 | 0.374 | 0.445 | 0.298 | 1.037 | 0.873  /0.951 | 0.372 | 0.000  /0.000 | 0.015 | Up-regulated by ABA |
| *C4JAR6 | rubisco subunit binding-protein beta subunit | sSEGTGSFPsPAAsPQPSR | S(10): 100.0; S(14): 99.7 | 19 | 0.988 | 1.185 | 1.000 | 1.523 | 1.534 | 1.634 | 1.058 | 0.802  /0.884 | 1.563 | 0.003  /0.011 | 0.026 | Down-regulated by ABA |
| *C4XVE1 | translocon-associated protein alpha subunit precursor | vELGTGTTDANIDEWLEGTsFVQR | S(20): 98.0 | 77 | 0.689 | 0.784 | 0.801 | 0.372 | 0.641 | 0.418 | 0.758 | 0.205  /0.247 | 0.477 | 0.000  /0.000 | 0.059 | Down-regulated by osmotic stress with ABA-independent way |
| *G3K3T2 | guanine-nucleotide-exchange protein | lQkPPPPPAAEAQAPSTPTSsPsTPTSSSAQPGPLR | S(29): 30.4 | 15 | — | — | — | 1.540 | 2.130 | 1.504 |  | 1.000  /1.035 | 1.725 | 0.000  /0.003 |  | Down-regulated by ABA |
| K7THT9 | hypothetical protein ZEAMMB73_869515 | dGGDTVsmkWLHDSSFGIDsk | S(20): 98.4 | 15 | — | — | — | 3.216 | 2.670 | 3.779 |  | 1.000  /1.035 | 3.222 | 0.000  /0.000 |  | Down-regulated by ABA |
| *K7TSC5 | c3h11 c3h type transcription partial | gSVYSPGQSAtSPGQHAYQGAVTSWPLSR | S(5): 24.0; S(9): 24.0; T(11): 24.0; S(12): 24.0 | 17 | 1.067 | 1.231 | 0.988 | 2.551 | 1.924 | 1.994 | 1.095 | 0.768  /0.853 | 2.156 | 0.000  /0.000 | 0.044 | Down-regulated by ABA |
| ***K7TTE2** | pentatricopeptide repeat-containing protein at2g13600-like | sAPsLPGAR | S(4): 100.0 | 21 | 0.776 | 0.833 | 0.835 | 1.571 | 1.655 | 1.397 | 0.815 | 0.362  /0.420 | 1.541 | 0.004  /0.014 | 0.013 | Down-regulated by ABA |
| K7TUB2 | hypothetical protein ZEAMMB73_551737 | lVLttEsLkHGLNMLQSmQNDNk | T(4): 95.6; T(5): 95.6 | 18 | — | — | — | 1.641 | 1.481 | 1.553 |  | 1.000  /1.035 | 1.558 | 0.001  /0.005 |  | Down-regulated by ABA |
| K7TVQ3 | thioredoxin domain-containing protein 9 homolog | qSGTGDssDsE | S(7): 100.0; S(8): 100.0; S(10): 100.0 | 21 | — | — | — | 3.111 | 2.321 | 2.527 |  | 1.000  /1.035 | 2.653 | 0.000  /0.000 |  | Down-regulated by ABA |
| *K7UCK7 | zinc finger ccch type domain-containing protein zfn-like 6 | sQPPDAAASPDASIssPSSLGGGGGDAADADAIEk | S(13): 39.2; S(15): 39.2; S(16): 39.2; S(18): 39.2; S(19): 39.2 | 22 | 0.998 | 0.899 | 1.031 | 0.656 | 0.645 | 0.663 | 0.976 | 0.976  /1.054 | 0.655 | 0.002  /0.008 | 0.011 | Up-regulated by ABA |
| K7UH11 | probable lysine-specific demethylase jmj14-like | fDQIADEDsDAEVADk | S(9): 100.0 | 12 | — | — | — | 0.598 | 0.662 | 0.644 |  | 1.000  /1.035 | 0.635 | 0.001  /0.004 |  | Up-regulated by ABA |
| *K7UQ69 | 14-3-3-like protein | dNLtLWSADsTTEDGIEEGNEASk | T(4): 91.1 | 26 | 0.789 | 0.929 | 0.960 | 0.567 | 0.653 | 0.638 | 0.893 | 0.624  /0.708 | 0.619 | 0.001  /0.004 | 0.011 | Up-regulated by ABA |
| *K7UVS4 | autophagy-related protein 18h-like isoform x2 | lSPQNLtPsPGVsPSTSPSSGSLVAR | T(7): 91.5 | 16 | — | — | — | 3.232 | 2.412 | 2.619 |  | 1.000  /1.035 | 2.754 | 0.000  /0.000 |  | Down-regulated by ABA |
| *K7UVX0 | hypothetical protein ZEAMMB73_982796, partial | aNVVsPVSNFDEALSEGsPLDTATR | S(5): 99.3; S(18): 88.9 | 38 | — | — | — | 2.016 | 2.509 | 1.782 |  | 1.000  /1.035 | 2.102 | 0.000  /0.000 |  | Down-regulated by ABA |
| *K7UZ73 | coiled-coil domain-containing protein 94-like | qTNVLQSLcQNYDsDDsE | S(7): 96.8; | 22 | 1.119 | 1.230 | 0.999 | 1.563 | 2.091 | 1.787 | 1.116 | 0.526  /0.062 | 1.814 | 0.003  /0.011 | 0.032 | Down-regulated by ABA |
| *K7V1A7 | chromatin structure-remodeling complex protein syd-like isoform x4 | dVSSIQsTSNELPNINsPLYEk | S(17): 99.7 | 39 | 1.032 | 1.200 | 1.213 | 1.502 | 1.752 | 1.650 | 1.148 | 0.516  /0.0593 | 1.634 | 0.001  /0.006 | 0.005 | Down-regulated by ABA |
| K7V2K3 | hypothetical protein ZEAMMB73_914991 | mLPAAAtAAAPDPScSTFPSSRRAPPVAsR | T(7): 28.6; S(14): 28.6; S(16): 28.6; T(17): 28.6; S(20): 28.6; S(21): 28.6; S(29): 28.6 | 22 | — | — | — | 2.887 | 1.899 | 2.623 |  | 1.000  /1.035 | 2.470 | 0.000  /0.000 |  | Down-regulated by ABA |
| *K7V4S4 | dentin sialophospho | aQDNSSPVVFDQYDsDVEQENLLDTFSSk | Y(13): 50.0; S(15): 50.0 | 32 | 0.881 | 0.822 | 1.089 | 0.615 | 0.580 | 0.769 | 0.931 | 0.740  /0.826 | 0.655 | 0.002  /0.009 | 0.007 | Up-regulated by ABA |
| *K7V5K5 | villin-2-like isoform 1 | tDVDRPVITPAGPsGPssPQSEAGESNVFR | S(17): 91.0; S(18): 91.0; S(21): 91.0 | 14 | 0.921 | 0.899 | 0.955 | 1.789 | 1.498 | 1.581 | 0.925 | 0.747  /0.832 | 1.623 | 0.001  /0.005 | 0.015 | Down-regulated by ABA |
| K7V5N5 | uncharacterized loc101210328 | gGAATPTRPPPEVSsPTSGGAkNmAVTSR | T(5): 16.4; T(7): 16.4; S(14): 16.4; S(15): 16.4; T(17): 16.4; S(18): 16.4 | 16 | 0.993 | 1.010 | 1.146 | 2.269 | 2.415 | 1.953 | 1.050 | 0.790  /0.875 | 2.212 | 0.000  /0.000 | 0.024 | Down-regulated by ABA |
| *K7V792 | splicing factor 3b subunit 1-like isoform x1 | mADADAtPAAGGAtPGATPSGAWDAtPk | T(7): 99.8; T(18): 92.0; T(26): 100.0 | 17 | — | — | — | 1.501 | 2.241 | 1.524 |  | 1.000  /1.035 | 1.755 | 0.000  /0.002 |  | Down-regulated by ABA |
| K7V9R4 | tpa: glycosyltransferase | asSPSGPGVPAALLPPVSSEEAANStLEsGVk | S(29): 99.6 | 14 | 0.988 | 1.181 | 1.321 | 0.289 | 0.376 | 0.402 | 1.163 | 0.502  /0.578 | 0.356 | 0.000  /0.000 | 0.006 | Up-regulated by ABA |
| *K7VDA6 | glycosyltransferase | sSAAAVRSsAGGAAGGDGAVADGGGAGPR | S(8): 39.5; S(9): 39.5 | 18 | 0.678 | 0.870 | 0.825 | 0.578 | 0.654 | 0.646 | 0.791 | 0.317  /0.375 | 0.626 | 0.001  /0.005 | 0.040 | Up-regulated by ABA |
| *K7VF00 | cell division cycle and apoptosis regulator protein 1 | nVEssADVGNSQTk | S(4): 100.0; S(5): 99.9 | 30 | 0.982 | 1.110 | 0.781 | 1.605 | 1.565 | 1.787 | 0.958 | 0.825  /0.905 | 1.652 | 0.001  /0.004 | 0.051 | Down-regulated by ABA |
| *K7VGC6 | cyclin-dependent kinase family protein | iPDLNLQDGPMVLsPPR | S(14): 100.0 | 42 | 1.101 | 1.199 | 1.389 | 0.523 | 0.658 | 0.766 | 1.230 | 0.401  /0.463 | 0.649 | 0.001  /0.006 | 0.002 | Up-regulated by ABA |
| K7VGD0 | myb dna-binding domain superfamily protein | fDPVNIDsDDDEWQAGDDGEYPITGR | S(8): 99.9 | 11 | — | — | — | 0.353 | 0.561 | 0.458 |  | 1.000  /1.035 | 0.457 | 0.000  /0.000 |  | Up-regulated by ABA |
| K7VHC0 | dead-box atp-dependent rna helicase family protein | vsDLDDDEDSDDDLVGFGDLDGk | S(2): 50.0; S(10): 50.0 | 15 | — | — | — | 2.101 | 1.709 | 1.501 |  | 1.000  /1.035 | 1.770 | 0.000  /0.001 |  | Down-regulated by ABA |
| *K7VKP3 | sap domain containing expressed | kDsPEGGsPEk | S(3): 100.0; S(8): 100.0 | 31 | 1.197 | 1.173 | 1.297 | 1.541 | 1.611 | 1.473 | 1.222 | 0.147  /0.178 | 1.542 | 0.004  /0.016 | 0.053 | Down-regulated by ABA |
| K7VLM3;B4FHS7 | epidermal growth factor receptor substrate 15-like 1-like isoform x1 | esYSDHGGSEsVFGDk | S(2): 33.3; Y(3): 33.3; S(4): 33.3; S(9): 95.2 | 10 | 1.612 | 1.493 | 1.501 | 1.566 | 1.481 | 1.489 | 1.535 | 0.067  /0.083 | 1.512 | 0.005  /0.017 | 0.176 | Up-regulated by osmotic stress with ABA-independent way |
| *P05022 | atp synthase cf1 alpha subunit | fSLQEQt | T(7): 100.0 | 43 | 1.275 | 0.994 | 1.210 | 2.659 | 1.939 | 3.123 | 1.160 | 0.446  /0.515 | 2.574 | 0.000  /0.000 | 0.037 | Down-regulated by ABA |
| *P11143 | probable mediator of rna polymerase ii transcription subunit 37c-like | myxGEGAGMGAAAGMDEDAPSGGSGAGPk | Y(2): 47.8; S(21): 47.8 | 18 | — | — | — | 2.165 | 2.369 | 2.992 |  | 1.000  /1.035 | 2.509 | 0.000  /0.000 |  | Down-regulated by ABA |
| P22275 | tubulin alpha chain | eDLAALEkDYEEVAAEGGsDDGDEEEEY | S(19): 50.0; Y(28): 50.0 | 17 | — | — | — | 0.365 | 0.431 | 0.456 |  | 1.000  /1.035 | 0.417 | 0.000  /0.000 |  | Up-regulated by ABA |
| *P31927 | sucrose-phosphate synthase | eATEDLAEDLsEGEkGDTIGELAPVETTk | S(11): 50.0; T(18): 50.0 | 19 | 1.456 | 1.276 | 1.634 | 1.567 | 1.459 | 2.803 | 1.455 | 0.105  /0.129 | 1.943 | 0.000 | 0.289 | Up-regulated by osmotic stress with ABA-independent way |
| Q41790 | calcium-dependent protein kinase | aPAPDsGR | S(6): 100.0 | 23 | 1.172 | 1.005 | 1.221 | 1.501 | 1.646 | 1.578 | 1.133 | 0.283  /0.335 | 1.575 | 0.000  /0.014 | 0.047 | Down-regulated by ABA |
| *Q8H6B1 | fact complex subunit spt16 | eHGAEsDsEEER | S(6): 100.0; S(8): 100.0 | 25 | 0.688 | 0.787 | 0.863 | 2.120 | 1.670 | 1.812 | 0.779 | 0.268 | 1.867 | 0.000  /0.000 | 0.024 | Down-regulated by ABA |
| Q9ZTL2 | cell wall invertase 1 | ssLsPDLykPTLAGLVDADISSGk | Y(8): 90.1; T(11): 90.1 | 11 | — | — | — | 0.302 | 0.375 | 0.454 |  | 1.000  /1.035 | 0.377 | 0.000  /0.000 |  | Up-regulated by ABA |

**Note**: ^a, b^ Each value represents the average of three biological replicas. The average is significant at a p <0.05 and a false discovery rate (FDR) < 0.05 level. Moreover, these peptides whose UniProt ID are signed with * are also significant under FDR<0.01. FDR values attained by Benjamini-Hochberg method were shown in column and were used to adjust p-values (correction for multiple comparisons). These phosphopeptides whose FDR values were signed with delete line ‘’ were not significant. ‘-’, not measured. T-test is used to identify whether the difference is significant. A T-test value <0.05 is considered to be significant. OS = osmotic stress.

**Table S3**︱Comparison of the phosphoprylation level and abundance of phosphoproteins (listed in Table 1) in maize leaves

| **Protein accession** | **Protein name** | **Phosphorylation level/protein abundance** | ***Vp5*: OS/control** | **P-Value** | ***vp5*: OS/control** | **P-Value** |
| --- | --- | --- | --- | --- | --- | --- |
|  |  |  |  |  |  |  |
| K7V8B2 | tata-binding protein-associated factor 172-like | Phosphorylation level | 3.301 | 0.000 | 0.425 | 0.000 |
|  |  | Protein abundance | 1.303 | 0.024 | 0.990 | 0.866 |
| C0PLA9 | nodulin-like protein | Phosphorylation level | 1.536 | 0.004 | 0.476 | 0.000 |
|  |  | Protein abundance | 0.925 | 0.491 | 0.854 | 0.478 |
| B4FBC | patellin family protein | Phosphorylation level | 2.416 | 0.000 | 0.452 | 0.000 |
|  |  | Protein abundance | 1.008 | 0.946 | 0.971 | 0.622 |
| K7TWZ6 | clustered mitochondria isoform x1 | Phosphorylation level | 2.394 | 0.000 | 0.551 | 0.000 |
|  |  | Protein abundance | 0.946 | 0.624 | 0.967 | 0.574 |
| K7V8M7 | mdr-like abc transporter | Phosphorylation level | 0.616 | 0.010 | 0.577 | 0.001 |
|  |  | Protein abundance | 0.882 | 0.267 | 0.924 | 0.186 |
| B8A0C6 | phosphatidate phosphatase lpin2-like | Phosphorylation level | 1.997 | 0.001 | 0.586 | 0.010 |
|  |  | Protein abundance | 0.965 | 0.753 | 0.956 | 0.451 |
| B4FWX5 | dihydroxy-acid mitochondrial-like | Phosphorylation level | 1.792 | 0.008 | 0.603 | 0.000 |
|  |  | Protein abundance | 1.029 | 0.807 | 1.038 | 0.529 |
| B4FS10 | TPA: hypothetical protein ZEAMMB73_767959 | Phosphorylation level | 0.544 | 0.006 | 0.646 | 0.000 |
|  |  | Protein abundance | 0.874 | 0.234 | 0.939 | 0.292 |
| B8A0C6 | phosphatidate phosphatase lpin2-like | Phosphorylation level | 1.788 | 0.011 | 0.621 | 0.001 |
|  |  | Protein abundance | 0.965 | 0.753 | 0.956 | 0.451 |
| K7V792 | splicing factor 3b subunit 1-like isoform x1 | Phosphorylation level | 1.790 | 0.001 | 0.650 | 0.001 |
|  |  | Protein abundance | 1.030 | 0.800 | 1.005 | 0.933 |
| B6U1M6 | transposon protein | Phosphorylation level | 0.527 | 0.005 | 1.530 | 0.005 |
|  |  | Protein abundance | 1.097 | 0.428 | 1.010 | 0.867 |
| B6U0Y9 | atp binding protein | Phosphorylation level | 1.520 | 0.000 | 1.598 | 0.000 |
|  |  | Protein abundance | 1.221 | 0.088 | 1.024 | 0.689 |
| B6SP06 | glycine-rich protein 2b | Phosphorylation level | 2.052 | 0.002 | 1.621 | 0.005 |
|  |  | Protein abundance | 0.828 | 0.095 | 1.038 | 0.529 |
| K7USN0 | 2og-fe oxygenase family protein | Phosphorylation level | 1.949 | 0.005 | 1.523 | 0.005 |
|  |  | Protein abundance | 0.891 | 0.308 | 0.934 | 0.253 |
| B4FKD1 | nucleoporin nup53-like | Phosphorylation level | 0.534 | 0.002 | 1.519 | 0.000 |
|  |  | Protein abundance | 0.919 | 0.455 | 0.984 | 0.787 |
| K7TW55 | translocase of chloroplast chloroplastic-like | Phosphorylation level | 1.628 | 0.004 | 1.798 | 0.000 |
|  |  | Protein abundance | 0.985 | 0.894 | 1.080 | 0.194 |
| M1H548 | arginine serine-rich protein 45-like | Phosphorylation level | 0.234 | 0.000 | 1.738 | 0.000 |
|  |  | Protein abundance | 1.005 | 0.966 | 0.977 | 0.697 |
| Q8W149 | cell division cycle 5-like | Phosphorylation level | 1.984 | 0.003 | 1.822 | 0.000 |
|  |  | Protein abundance | 0.895 | 0.327 | 0.988 | 0.840 |
| B8A298 | histone-lysine n- h3 lysine-9 specific suvh1-like | Phosphorylation level | 3.255 | 0.000 | 2.120 | 0.000 |
|  |  | Protein abundance | 1.081 | 0.505 | 0.954 | 0.431 |
| P24993 | photosystem ii phosphoprotein | Phosphorylation level | 1.761 | 0.002 | 2.987 | 0.000 |
|  |  | Protein abundance | 1.369 | 0.107 | 0.870 | 0.420 |

**Table S4︱Comparison of the phosphoprylation level and abundance of phosphoproteins (listed in Table S1) in maize leaves**

| **Protein accession** | **Protein name** | **Phosphorylation level**  **/protein abundance** | *Vp5*: OS/control | **P-Value** | *vp5*: OS/control | **P-Value** |
| --- | --- | --- | --- | --- | --- | --- |
|  |  |  |  |  |  |  |
| *B4FAW3 | photosystem i reaction center subunit ii | Phosphorylation level | 2.281 | 0.003 | 1.295 | 0.027 |
|  |  | Protein abundance | 0.775 | 0.024 | 0.968 | 0.586 |
| *B4FDJ3 | epidermal growth factor receptor substrate 15-like 1-like isoform x2 | Phosphorylation level | 0.204 | 0.000 | 0.912 | 0.467 |
|  |  | Protein abundance | 0.877 | 0.246 | 1.033 | 0.583 |
| *B4FFB7 | dead-box atp-dependent rna helicase family protein | Phosphorylation level | 0.639 | 0.043 | 0.903 | 0.416 |
|  |  | Protein abundance | 0.948 | 0.637 | 0.964 | 0.539 |
| B4FGQ3 | probable receptor-like protein kinase at5g56460-like | Phosphorylation level | 0.614 | 0.028 | 1.231 | 0.164 |
|  |  | Protein abundance | 0.950 | 0.650 | 1.047 | 0.438 |
| *B4FJ52 | bri1-kd interacting protein 128 | Phosphorylation level | 1.700 | 0.031 | 1.254 | 0.500 |
|  |  | Protein abundance | 0.914 | 0.427 | 1.047 | 0.438 |
| *B4FK28 | tpa: rna-binding protein | Phosphorylation level | 0.597 | 0.022 | 0.866 | 0.272 |
|  |  |  | 0.588 | 0.002 | 1.031 | 0.002 |
|  |  | Protein abundance | 0.876 | 0.242 | 0.981 | 0.748 |
| B4FLK4 | zinc finger protein 207-like isoform x1 | Phosphorylation level | 0.436 | 0.000 | 1.293 | 0.086 |
|  |  | Protein abundance | 1.073 | 0.547 | 0.960 | 0.494 |
| B4FN51 | protein grpe-like | Phosphorylation level | 1.792 | 0.014 | 0.908 | 0.487 |
|  |  | Protein abundance | 1.136 | 0.275 | 1.069 | 0.260 |
| *B4FPT5 | uncharacterized protein LOC100272657 | Phosphorylation level | 0.630 | 0.043 | 1.202 | 0.097 |
|  |  | Protein abundance | 0.898 | 0.342 | 1.096 | 0.122 |
| *B4FPV9 | actin-related protein 9-like | Phosphorylation level | 0.553 | 0.008 | 0.977 | 0.994 |
|  |  | Protein abundance | 1.068 | 0.574 | 1.057 | 0.349 |
| *B4FQ71 | tpa: duf1421 domain family protein | Phosphorylation level | 0.519 | 0.004 | 0.907 | 0.145 |
|  |  | Protein abundance | 1.042 | 0.725 | 1.020 | 0.738 |
| *B4FQT3 | heat shock protein sti | Phosphorylation level | 0.615 | 0.007 | 0.877 | 0.654 |
|  |  | Protein abundance | 0.967 | 0.767 | 1.046 | 0.448 |
| *B4FRW3 | gbp17 type transcription partial | Phosphorylation level | 1.716 | 0.023 | 1.227 | 0.121 |
|  |  | Protein abundance | 1.021 | 0.859 | 1.022 | 0.713 |
| B4FS10 | TPA: hypothetical protein ZEAMMB73_767959 | Phosphorylation level | 0.336 | 0.000 | 0.874 | 0.254 |
|  |  | Protein abundance | 0.874 | 0.234 | 0.939 | 0.292 |
| *B4FSE2 | protochlorophyllide reductase b | Phosphorylation level | 1.701 | 0.022 | 1.048 | 0.934 |
|  |  | Protein abundance | 0.935 | 0.553 | 0.935 | 0.261 |
| *B4FTA7 | pgr5-like a isoform 1 | Phosphorylation level | 1.754 | 0.017 | 0.913 | 0.453 |
|  |  | Protein abundance | 0.869 | 0.215 | 0.977 | 0.697 |
| B4FTR7 | tab2 protein | Phosphorylation level | 0.555 | 0.006 | 0.877 | 0.272 |
|  |  | Protein abundance | 1.054 | 0.653 | 0.984 | 0.787 |
| B4FTY8 | harpin inducing protein | Phosphorylation level | 1.770 | 0.014 | 0.835 | 0.287 |
|  |  | Protein abundance | 0.963 | 0.739 | 1.057 | 0.349 |
| *B4FUX9 | coiled-coil domain-containing protein 12-like | Phosphorylation level | 0.572 | 0.013 | 0.876 | 0.214 |
|  |  | Protein abundance | 0.934 | 0.546 | 1.032 | 0.595 |
| *B4FVB8 | serine threonine-protein kinase chloroplastic-like | Phosphorylation level | 1.509 | 0.037 | 0.943 | 0.949 |
|  |  | Protein abundance | 1.102 | 0.406 | 0.923 | 0.180 |
| *B4FWC4 | rna-binding protein 39-like isoform x1 | Phosphorylation level | 2.213 | 0.001 | 1.009 | 0.720 |
|  |  |  | 0.645 | 0.083 | 1.036 | 0.014 |
|  |  | Protein abundance | 0.843 | 0.131 | 1.027 | 0.653 |
| *B4FY62 | tpa: c3hc zinc finger-like family protein | Phosphorylation level | 0.442 | 0.000 | 1.041 | 0891 |
|  |  |  | 2.288 | 0.001 | 1.399 | 0.017 |
|  |  | Protein abundance | 0.984 | 0.887 | 1.112 | 0.973 |
| B4FYD7 | ranbp1 domain containing protein | Phosphorylation level | 0.467 | 0.000 | 1.038 | 0.512 |
|  |  | Protein abundance | 1.008 | 0.946 | 1.050 | 0.410 |
| B4FZ13 | unknown | Phosphorylation level | 0.211 | 0.000 | 1.149 | 0.138 |
|  |  | Protein abundance | 1.045 | 0.707 | 1.038 | 0.529 |
| *B4FZY1 | Na+/ H+ antiporter | Phosphorylation level | 2.913 | 0.000 | 0.954 | 0.396 |
|  |  | Protein abundance | 1.253 | 0.054 | 0.986 | 0.813 |
| *B4G0Y5 | katanin p60 atpase-containing subunit | Phosphorylation level | 0.587 | 0.019 | 1.221 | 0.085 |
|  |  | Protein abundance | 0.818 | 0.076 | 1.040 | 0.508 |
| B4G0Z1 | e3 ubiquitin-protein ligase ubr7-like | Phosphorylation level | 0.553 | 0.001 | 1.177 | 0.221 |
|  |  | Protein abundance | 1.005 | 0.966 | 0.984 | 0.787 |
| ***B4G1E6** | pro-resilin precursor | Phosphorylation level | 1.634 | 0.039 | 1.207 | 0.164 |
|  |  | Protein abundance | **1.085** | **0.485** | **1.889** | **0.000** |
| *B4G1V3 | ribonucleoprotein chloroplastic-like | Phosphorylation level | 0.522 | 0.014 | 0.871 | 0.190 |
|  |  | Protein abundance | 0.990 | 0.929 | 0.893 | 0.758 |
| *B4G217 | peptidyl-prolyl cis-trans isomerase g-like isoform x4 | Phosphorylation level | 0.311 | 0.000 | 1.034 | 0.851 |
|  |  | Protein abundance | 1.044 | 0.713 | 1.213 | 0.601 |
| *B6SJN1 | auxin-repressed kda protein | Phosphorylation level | 2.057 | 0.006 | 1.014 | 0.745 |
|  |  | Protein abundance | 0.995 | 0.965 | 1.053 | 0.383 |
| *B6SKU4;C0PIL1 | kinesin light chain-like protein | Phosphorylation level | 0.523 | 0.030 | 0.911 | 0.375 |
|  |  | Protein abundance | 0.915 | 0.432 | 1.119 | 0.658 |
| *B6SS20 | tpa: phototropin family protein kinase | Phosphorylation level | 2.021 | 0.003 | 1.046 | 0.853 |
|  |  |  | 1.763 | 0.019 | 1.271 | 0.380 |
|  |  | Protein abundance | 1.087 | 0.475 | 1.015 | 0.801 |
| *B6SSK6;B4FA24 | nucleolar rna helicase 2 | Phosphorylation level | 0.485 | 0.001 | 0.881 | 0.138 |
|  |  | Protein abundance | 0.790 | 0.037 | 0.989 | 0.853 |
| B6SSY1 | calcium ion binding protein | Phosphorylation level | 2.964 | 0.001 | 1.334 | 0.138 |
|  |  | Protein abundance | 1.011 | 0.925 | 0.958 | 0.473 |
| *B6STN4 | chlorophyll a-b binding protein 2 | Phosphorylation level | 0.561 | 0.016 | 1.054 | 0.994 |
|  |  | Protein abundance | **1.621** | **0.000** | **0.863** | **0.614** |
| *B6SW01 | zinc finger ccch type domain-containing protein zfn-like 3 | Phosphorylation level | 0.527 | 0.016 | 0.967 | 0.994 |
|  |  | Protein abundance | 1.060 | 0.618 | 1.104 | 0.095 |
| B6SW97 | protein fam188a-like | Phosphorylation level | 0.461 | 0.001 | 1.079 | 0.469 |
|  |  | Protein abundance | 0.982 | 0.872 | 1.028 | 0.641 |
| B6SWM4 | bel1-related homeotic protein 30 | Phosphorylation level | 0.566 | 0.015 | 1.001 | 0.908 |
|  |  | Protein abundance | 0.915 | 0.432 | 1.058 | 0.341 |
| *B6SX66 | uncharacterized protein LOC100275650 | Phosphorylation level | 2.251 | 0.001 | 1.139 | 0.320 |
|  |  | Protein abundance | 1.139 | 0.266 | 0.851 | 0.657 |
| *B6T0F0 | probable -trehalose-phosphate synthase | Phosphorylation level | 1.611 | 0.041 | 1.056 | 0.681 |
|  |  | Protein abundance | **1.342** | **0.012** | **0.912** | **0.123** |
| *B6T245 | zn- - containing protein | Phosphorylation level | 1.714 | 0.022 | 1.045 | 0.780 |
|  |  |  | 0.509 | 0.005 | 1.224 | 0.085 |
|  |  | Protein abundance | 0.984 | 0.887 | 0.886 | 0.743 |
| B6T2A6 | stem-specific protein tsjt1-like | Phosphorylation level | 0.438 | 0.000 | 0.742 | 0.125 |
|  |  | Protein abundance | 0.898 | 0.342 | 0.858 | 0.610 |
| *B6T671 | uncharacterized loc101221005 | Phosphorylation level | 1.637 | 0.047 | 1.068 | 0.537 |
|  |  | Protein abundance | 1.247 | 0.359 | 0.986 | 0.813 |
| *B6T6R3 | probable calcium-binding protein cml22-like | Phosphorylation level | 0.498 | 0.002 | 1.029 | 0.188 |
|  |  | Protein abundance | **1.482** | **0.001** | **0.862** | **0.613** |
| *B6T6V5 | ubiquitin carboxyl-terminal hydrolase 6-like | Phosphorylation level | 0.618 | 0.029 | 1.213 | 0.169 |
|  |  |  | 0.561 | 0.008 | 1.354 | 0.048 |
|  |  | Protein abundance | 0.981 | 0.865 | 1.025 | 0.677 |
| *B6T7C2 | eukaryotic translation initiation factor 5 | Phosphorylation level | 1.783 | 0.024 | 0.777 | 0.625 |
|  |  | Protein abundance | 0.991 | 0.936 | 1.021 | 0.726 |
| *B6T7I1 | rna binding protein | Phosphorylation level | 0.601 | 0.044 | 1.005 | 0.943 |
|  |  | Protein abundance | 0.932 | 0.534 | 0.954 | 0.431 |
| *B6T883 | multidomain cystatin | Phosphorylation level | 0.401 | 0.000 | 1.075 | 0.446 |
|  |  | Protein abundance | 0.966 | 0.760 | 1.022 | 0.713 |
| *B6TC04 | fibrous sheath cabyr-binding | Phosphorylation level | 1.800 | 0.011 | 1.040 | 0.945 |
|  |  | Protein abundance | 0.868 | 0.211 | 1.043 | 0.477 |
| B6TD33 | zinc finger ccch domain-containing protein 11-like | Phosphorylation level | 1.691 | 0.027 | 1.228 | 0.149 |
|  |  | Protein abundance | 0.915 | 0.432 | 0.944 | 0.335 |
| *B6TE60 | probable proteasome inhibitor-like | Phosphorylation level | 0.593 | 0.026 | 0.977 | 0.458 |
|  |  | Protein abundance | 0.983 | 0.880 | 1.005 | 0.933 |
| *B6TEF1 | rpm1-interacting protein 4-like isoform x4 | Phosphorylation level | 1.678 | 0.019 | 1.061 | 0.811 |
|  |  | Protein abundance | 1.121 | 0.329 | 1.078 | 0.205 |
| *B6TI42 | at-hook protein 1 | Phosphorylation level | 0.575 | 0.024 | 0.923 | 0.709 |
|  |  |  | 0.339 | 0.033 | 1.177 | 0.006 |
|  |  | Protein abundance | 1.156 | 0.215 | 0.998 | 0.973 |
| ***B6TM56** | chloroplast outer envelope 24 kd protein | Phosphorylation level | 0.270 | 0.000 | 1.345 | 0.022 |
|  |  | Protein abundance | **0.597** | **0.000** | **1.537** | **0.000** |
| *B6TPC9 | zn- - containing protein | Phosphorylation level | 0.653 | 0.000 | 1.076 | 0.791 |
|  |  | Protein abundance | **1.446** | **0.052** | **0.831** | **0.102** |
| *B6TPG2 | 60s ribosomal protein l26-1 | Phosphorylation level | 0.455 | 0.000 | 1.295 | 0.075 |
|  |  | Protein abundance | 0.867 | 0.207 | 0.951 | 0.400 |
| *B6TS38 | ribose-5-phosphate isomerase | Phosphorylation level | 0.618 | 0.013 | 1.031 | 0.698 |
|  |  | Protein abundance | 1.037 | 0.756 | 1.011 | 0.853 |
| *B6U194 | zinc finger c-x8-c-x5-c-x3-h type family protein | Phosphorylation level | 0.443 | 0.000 | 0.905 | 0.411 |
|  |  | Protein abundance | 1.118 | 0.340 | 0.896 | 0.066 |
| *B6U1Z4 | atp binding protein | Phosphorylation level | 2.398 | 0.000 | 1.011 | 0.967 |
|  |  | Protein abundance | 0.919 | 0.455 | 1.014 | 0.814 |
| *B6U3A0 | glycine-rich rna-binding protein 7 | Phosphorylation level | 0.409 | 0.000 | 0.968 | 0.163 |
|  |  | Protein abundance | 0.978 | 0.844 | 1.023 | 0.701 |
| *B6U6U2 | hexose transporter | Phosphorylation level | 1.670 | 0.026 | 1.071 | 0.580 |
|  |  | Protein abundance | 1.058 | 0.630 | 0.974 | 0.659 |
| *B6UB08 | zinc finger protein 652-a- partial | Phosphorylation level | 0.552 | 0.006 | 0.903 | 0.101 |
|  |  | Protein abundance | 1.011 | 0.925 | 1.081 | 0.188 |
| *B6UBN4 | j domain-containing protein required for chloroplast accumulation response 1-like isoform x2 | Phosphorylation level | 0.454 | 0.000 | 1.006 | 0.891 |
|  |  |  | 0.617 | 0.050 | 1.117 | 0.200 |
|  |  | Protein abundance | 1.060 | 0.618 | 0.985 | 0.800 |
| *B6UEI4 | protein gdap2 homolog | Phosphorylation level | 1.748 | 0.018 | 0.774 | 0.041 |
|  |  | Protein abundance | 0.849 | 0.148 | 1.000 | 1.000 |
| *B6UEP1 | transcription factor hy5 | Phosphorylation level | 3.932 | 0.000 | 0.798 | 0.041 |
|  |  | Protein abundance | 0.961 | 0.725 | 1.124 | 0.048 |
| B6UIQ0 | histone h2a | Phosphorylation level | 1.595 | 0.048 | 1.133 | 0.218 |
|  |  | Protein abundance | 1.181 | 0.155 | 0.983 | 0.774 |
| *B7ZXU2 | serrate-related c2h2 zinc-finger family protein | Phosphorylation level | 1.611 | 0.048 | 1.224 | 0.151 |
|  |  | Protein abundance | 0.869 | 0.215 | 0.969 | 0.598 |
| *B7ZYP6 | pyruvate orthophosphate dikinase | Phosphorylation level | 0.589 | 0.000 | 0.999 | 0.937 |
|  |  | Protein abundance | 0.943 | 0.604 | 0.987 | 0.827 |
| *B7ZYR5 | tpa: leucine-rich repeat receptor-like protein kinase family protein | Phosphorylation level | 0.385 | 0.000 | 1.130 | 0.109 |
|  |  | Protein abundance | 1.017 | 0.885 | 1.069 | 0.260 |
| *B7ZZ27 | spf1-like dna-binding protein | Phosphorylation level | 0.569 | 0.012 | 0.996 | 0.832 |
|  |  | Protein abundance | 0.932 | 0.534 | 1.002 | 0.973 |
| B8A0M9 | tpa: map kinase family protein isoform 1 | Phosphorylation level | 2.941 | 0.000 | 1.211 | 0.169 |
|  |  | Protein abundance | 1.087 | 0.475 | 0.989 | 0.853 |
| *B8A134 | heterogeneous nuclear ribonucleoprotein 1-like | Phosphorylation level | 0.583 | 0.015 | 0.859 | 0.202 |
|  |  |  | 2.247 | 0.001 | 1.057 | 0.289 |
|  |  | Protein abundance | 0.819 | 0.078 | 1.076 | 0.216 |
| *B8A287 | c2 domain-containing expressed | Phosphorylation level | 1.740 | 0.042 | 1.105 | 0.838 |
|  |  | Protein abundance | 1.001 | 0.993 | 1.056 | 0.357 |
| B8A305 | heterogeneous nuclear ribonucleoprotein 1-like isoform x1 | Phosphorylation level | 0.616 | 0.031 | 1.100 | 0.508 |
|  |  | Protein abundance | 0.932 | 0.534 | 1.040 | 0.508 |
| *B8A307 | transmembrane expressed | Phosphorylation level | 0.513 | 0.004 | 0.956 | 0.358 |
|  |  |  | 1.725 | 0.012 | 1.092 | 0.558 |
|  |  | Protein abundance | 0.975 | 0.823 | 1.033 | 0.583 |
| *B8A367 | cysteine chloroplastic chromoplastic-like | Phosphorylation level | 0.468 | 0.000 | 0.865 | 0.1999 |
|  |  | Protein abundance | 0.863 | 0.193 | 0.926 | 0.198 |
| C0HE85 | eukaryotic translation initiation factor 2a-like | Phosphorylation level | 0.352 | 0.000 | 1.102 | 0.607 |
|  |  | Protein abundance | 1.073 | 0.547 | 0.950 | 0.391 |
| *C0HE93 | tumor susceptibility gene 101 family protein | Phosphorylation level | 0.307 | 0.000 | 0.995 | 0.897 |
|  |  | Protein abundance | 0.909 | 0.399 | 1.035 | 0.561 |
| *C0HHJ0 | set domain protein sdg111 | Phosphorylation level | 0.388 | 0.000 | 0.972 | 0.771 |
|  |  | Protein abundance | 1.127 | 0.306 | 1.173 | 0.007 |
| *C0HHU2 | Uncharacterized protein | Phosphorylation level | 0.520 | 0.004 | 0.877 | 0.350 |
|  |  | Protein abundance | 0.882 | 0.267 | 1.093 | 0.133 |
| *C0HIM6 | integrin-linked protein kinase family protein | Phosphorylation level | 0.579 | 0.016 | 0.926 | 0.317 |
|  |  |  | 0.312 | 0.079 | 1.050 | 0.009 |
|  |  | Protein abundance | 0.969 | 0.781 | 0.999 | 0.987 |
| *C0HIV2 | serine hydroxymethyltransferase 4-like | Phosphorylation level | 0.583 | 0.017 | 1.001 | 0.851 |
|  |  | Protein abundance | 0.869 | 0.215 | 1.040 | 0.508 |
| C0P2B1 | phd zinc finger | Phosphorylation level | 2.351 | 0.000 | 1.463 | 0.008 |
|  |  | Protein abundance | 0.850 | 0.151 | 1.029 | 0.629 |
| C0P3H1 | ubiquitin carboxyl-terminal hydrolase isozyme l5-like | Phosphorylation level | 0.555 | 0.006 | 0.952 | 0.704 |
|  |  | Protein abundance | 1.057 | 0.635 | 0.999 | 0.987 |
| *C0P8J5 | tpa: act-domain containing protein kinase family protein | Phosphorylation level | 2.738 | 0.001 | 1.136 | 0.223 |
|  |  | Protein abundance | 1.017 | 0.885 | 1.040 | 0.508 |
| C0P8S9 | heterogeneous nuclearribonucleoprotein a2 | Phosphorylation level | 0.616 | 0.029 | 0.824 | 0.114 |
|  |  | Protein abundance | 0.781 | 0.029 | 1.002 | 0.973 |
| *C0P9I0 | unknown | Phosphorylation level | 1.979 | 0.004 | 1.139 | 0.967 |
|  |  |  | 1.973 | 0.004 | 1.023 | 0.620 |
|  |  | Protein abundance | 1.075 | 0.536 | 1.014 | 0.814 |
| *C0P9I5 | tbc1 domain family member 15-like | Phosphorylation level | 0.562 | 0.011 | 0.963 | 0.934 |
|  |  | Protein abundance | 0.888 | 0.294 | 1.068 | 0.267 |
| *C0PAY9 | snare-interacting protein keule | Phosphorylation level | 1.925 | 0.006 | 1.031 | 0.720 |
|  |  | Protein abundance | 1.034 | 0.775 | 0.939 | 0.292 |
| *C0PB33 | transcription elongation factor spt5-like | Phosphorylation level | 1.733 | 0.022 | 1.197 | 0.541 |
|  |  | Protein abundance | 1.110 | 0.372 | 0.949 | 0.381 |
| *C0PHF7 | fas-associated factor 2-b | Phosphorylation level | 1.865 | 0.007 | 1.049 | 0.811 |
|  |  | Protein abundance | 1.027 | 0.820 | 1.143 | 0.024 |
| *C0PLA9 | nodulin-like protein | Phosphorylation level | 1.699 | 0.042 | 0.784 | 0.236 |
|  |  | Protein abundance | 0.925 | 0.491 | 0.854 | 0.008 |
| *C0PMQ0 | pre-mrna-splicing factor syf1-like | Phosphorylation level | 1.772 | 0.016 | 1.053 | 0.940 |
|  |  | Protein abundance | 1.013 | 0.912 | 0.993 | 0.906 |
| *K7TFK8 | e3 ubiquitin-protein ligase upl1-like | Phosphorylation level | 2.007 | 0.009 | 1.051 | 0.473 |
|  |  |  | 1.661 | 0.029 | 1.424 | 0.020 |
|  |  | Protein abundance | 0.993 | 0.951 | 0.983 | 0.774 |
| *K7TL05 | 14-3-3-like protein gf14-12 | Phosphorylation level | 1.502 | 0.047 | 0.958 | 0.648 |
|  |  | Protein abundance | 1.192 | 0.133 | 1.185 | 0.004 |
| *K7TXK2 | far upstream element-binding protein 1-like | Phosphorylation level | 0.689 | 0.046 | 0.919 | 0.472 |
|  |  | Protein abundance | 1.002 | 0.986 | 1.070 | 0.253 |
| *K7TZ83 | sucrose-phosphate synthase family protein | Phosphorylation level | 0.455 | 0.000 | 1.067 | 0.180 |
|  |  | Protein abundance | 1.140 | 0.262 | 1.000 | 1.000 |
| *K7U0Y3 | dag protein | Phosphorylation level | 0.213 | 0.000 | 0.994 | 0.584 |
|  |  | Protein abundance | 0.804 | 0.054 | 1.035 | 0.561 |
| *K7U162 | rna polymerase ii-associated factor 1 homolog | Phosphorylation level | 0.594 | 0.000 | 0.914 | 0.458 |
|  |  | Protein abundance | 0.961 | 0.725 | 1.011 | 0.853 |
| ***K7U4E0** | protein furry homolog isoform x1 | Phosphorylation level | 0.536 | 0.006 | 1.005 | 0.532 |
|  |  |  | 0.581 | 0.015 | 1.376 | 0.037 |
|  |  | Protein abundance | 0.757 | 0.026 | 1.313 | 0.042 |
| *****K7U4Z8 | neurofilament heavy polypeptide-like | Phosphorylation level | 0.293 | 0.035 | 1.133 | 0.008 |
|  |  | Protein abundance | 0.992 | 0.943 | 1.017 | 0.776 |
| *K7U7Q6 | nuclear matrix constituent protein 1-like | Phosphorylation level | 0.329 | 0.000 | 0.838 | 0.097 |
|  |  | Protein abundance | 1.017 | 0.885 | 1.021 | 0.726 |
| K7UAY1 | serine threonine-protein kinase ctr1 | Phosphorylation level | 0.365 | 0.000 | 1.180 | 0.029 |
|  |  | Protein abundance | 0.896 | 0.332 | 0.993 | 0.906 |
| *K7UBL3 | zinc finger c-x8-c-x5-c-x3-h type family protein | Phosphorylation level | 0.535 | 0.002 | 1.293 | 0.131 |
|  |  | Protein abundance | 0.984 | 0.887 | 1.049 | 0.419 |
| K7UDG0 | plant-specific domain tigr01627 family protein | Phosphorylation level | 0.572 | 0.015 | 1.040 | 0.960 |
|  |  | Protein abundance | 1.066 | 0.585 | 0.976 | 0.684 |
| *K7UN43 | hypothetical protein ZEAMMB73_955518 | Phosphorylation level | 0.477 | 0.001 | 0.894 | 0.375 |
|  |  | Protein abundance | 0.976 | 0.830 | 0.937 | 0.276 |
| *K7UT89 | jumonji-like transcription factor family protein | Phosphorylation level | 2.399 | 0.000 | 1.208 | 0.186 |
|  |  |  | 2.924 | 0.000 | 1.240 | 0.120 |
|  |  | Protein abundance | 1.030 | 0.800 | 1.009 | 0.880 |
| *K7UTR3 | cell division cycle atpase-like | Phosphorylation level | 2.690 | 0.002 | 1.267 | 0.131 |
|  |  | Protein abundance | 0.965 | 0.753 | 1.046 | 0.448 |
| K7V1I2 | arginine serine-rich splicing factor sr45_2 transcript i | Phosphorylation level | 0.335 | 0.000 | 0.878 | 0.234 |
|  |  | Protein abundance | 0.905 | 0.378 | 1.008 | 0.893 |
| K7V3T9 | hypothetical protein ZEAMMB73_910657 | Phosphorylation level | 0.326 | 0.000 | 1.026 | 0.983 |
|  |  | Protein abundance | 1.096 | 0.433 | 1.093 | 0.133 |
| *K7V4D9 | e3 ubiquitin-protein ligase upl4-like | Phosphorylation level | 2.692 | 0.000 | 0.982 | 0.665 |
|  |  | Protein abundance | 1.049 | 0.682 | 0.975 | 0.672 |
| *K7VAC7 | bzip transcription factor superfamily protein | Phosphorylation level | 1.699 | 0.023 | 1.241 | 0.593 |
|  |  | Protein abundance | 0.815 | 0.071 | 1.044 | 0.467 |
| ***K7VBH0** | phd-finger family expressed | Phosphorylation level | 0.621 | 0.033 | 1.442 | 0.012 |
|  |  | Protein abundance | **1.379** | **0.006** | **1.046** | **0.448** |
| *K7VDG7 | duf1296 domain containing family protein | Phosphorylation level | 1.671 | 0.027 | 1.117 | 0.351 |
|  |  | Protein abundance | 0.869 | 0.215 | 1.009 | 0.880 |
| *K7VGC6 | cyclin-dependent kinase family protein | Phosphorylation level | 1.597 | 0.046 | 1.047 | 0.326 |
|  |  | Protein abundance | 1.034 | 0.775 | 0.968 | 0.586 |
| K7VHJ5 | anthocyanidin -o-glucosyltransferase-like | Phosphorylation level | 0.508 | 0.002 | 1.092 | 0.611 |
|  |  | Protein abundance | 0.974 | 0.816 | 0.984 | 0.787 |
| K7VLM3 | epidermal growth factor receptor substrate 15-like 1-like isoform x1 | Phosphorylation level | 3.596 | 0.000 | 1.149 | 0.370 |
|  |  | Protein abundance | 1.074 | 0.541 | 0.983 | 0.774 |
| *K7VQB2 | loricrin-like isoform x1 | Phosphorylation level | 1.588 | 0.045 | 0.991 | 0.891 |
|  |  | Protein abundance | 0.899 | 0.347 | 1.072 | 0.240 |
| K7VYU4 | gap sh3 binding protein | Phosphorylation level | 0.640 | 0.046 | 0.855 | 0.138 |
|  |  | Protein abundance | 0.949 | 0.644 | 0.996 | 0.947 |
| *K7W1A6 | filament-like plant protein 4-like | Phosphorylation level | 2.382 | 0.000 | 0.935 | 0.439 |
|  |  | Protein abundance | 0.881 | 0.263 | 1.006 | 0.920 |
| *O48547 | nonphototropic hypocotyl protein expressed | Phosphorylation level | 1.597 | 0.044 | 1.030 | 0.358 |
|  |  |  | 1.905 | 0.005 | 1.008 | 0.691 |
|  |  |  | 1.774 | 0.013 | 1.109 | 0.380 |
|  |  | Protein abundance | 1.148 | 0.238 | 0.950 | 0.391 |
| *P22275 | phosphoenolpyruvate carboxylase | Phosphorylation level | 2.248 | 0.002 | 1.090 | 0.740 |
|  |  | Protein abundance | 0.983 | 0.880 | 0.987 | 0.827 |
| *P31927 | sucrose-phosphate synthase | Phosphorylation level | 0.603 | 0.035 | 1.074 | 0.903 |
|  |  | Protein abundance | 1.002 | 0.986 | 0.985 | 0.800 |
| P49106 | 14-3-3-like protein gf14-6 | Phosphorylation level | 2.354 | 0.000 | 1.058 | 0.926 |
|  |  | Protein abundance | 1.090 | 0.461 | 1.102 | 0.101 |
| *Q6JN48 | ethylene-insensitive protein 2-like | Phosphorylation level | 0.636 | 0.011 | 0.874 | 0.258 |
|  |  |  | 0.427 | 0.000 | 1.458 | 0.010 |
|  |  | Protein abundance | 1.023 | 0.846 | 1.025 | 0.677 |
| *Q6UNK5 | abc transporter b family member 1-like | Phosphorylation level | 2.258 | 0.001 | 1.334 | 0.047 |
|  |  | Protein abundance | 1.103 | 0.402 | 0.994 | 0.920 |
| *Q8W149 | cell division cycle 5-like | Phosphorylation level | 1.752 | 0.030 | 1.030 | 0.568 |
|  |  |  | 2.139 | 0.001 | 0.932 | 0.628 |
|  |  | Protein abundance | 0.895 | 0.327 | 0.988 | 0.840 |
| *Q94IQ9 | methyl- binding domain containing expressed | Phosphorylation level | 4.824 | 0.000 | 1.189 | 0.403 |
|  |  | Protein abundance | 0.925 | 0.491 | 1.058 | 0.341 |
| *Q9FER8 | hmgi y protein | Phosphorylation level | 0.359 | 0.000 | 0.931 | 0.537 |
|  |  | Protein abundance | 0.911 | 0.410 | 0.990 | 0.866 |

**Table S5︱Comparison of the phosphoprylation level and abundance of phosphoproteins (listed in Table S2) in maize leaves**

| **Protein accession** | **Protein name** | **Phosphorylation level/protein abundance** | ***Vp5*: OS/control** | **P-Value** | ***vp5*: OS/control** | **P-Value** |
| --- | --- | --- | --- | --- | --- | --- |
|  |  |  |  |  |  |  |
| *B4F9V2 | vesicle-associated protein 1-2-like | Phosphorylation level | 1.280 | 0.615 | 1.671 | 0.001 |
|  |  | Protein abundance | 1.132 | 0.289 | 0.993 | 0.906 |
| *B4FDJ3 | epidermal growth factor receptor substrate 15-like 1-like isoform x2 | Phosphorylation level | 0.788 | 0.280 | 1.588 | 0.002 |
|  |  | Protein abundance | 0.877 | 0.246 | 1.033 | 0.583 |
| *B4FKD1 | nucleoporin nup53-like | Phosphorylation level | 1.300 | 0.251 | 1.956 | 0.000 |
|  |  | Protein abundance | 0.919 | 0.455 | 0.984 | 0.787 |
| *B4FMB8 | nudix hydrolase 24 | Phosphorylation level | 0.995 | 0.997 | 0.644 | 0.003 |
|  |  | Protein abundance | 1.000 | 1.000 | 0.878 | 0.029 |
| *B4FPT5;B6SUQ3 | uncharacterized protein LOC100272657 | Phosphorylation level | 1.030 | 0.932 | 1.654 | 0.001 |
|  |  | Protein abundance | 0.898 | 0.342 | 1.096 | 0.122 |
| B4FQ59 | phosphoribulokinase precursor | Phosphorylation level | 0.980 | 0.858 | 1.577 | 0.002 |
|  |  | Protein abundance | 1.114 | 0.356 | 0.932 | 0.239 |
| *B4FRW3 | gbp17 type transcription partial | Phosphorylation level | 1.337 | 0.280 | 2.180 | 0.000 |
|  |  | Protein abundance | 1.021 | 0.859 | 1.022 | 0.713 |
| *B4FWI0 | 60s acidic ribosomal protein p0 | Phosphorylation level |  |  | 1.706 | 0.000 |
|  |  | Protein abundance | 1.063 | 0.601 | 1.041 | 0.497 |
| *B4FX77 | zinc finger c-x8-c-x5-c-x3-h type family protein | Phosphorylation level | 1.412 | 0.143 | 0.620 | 0.001 |
| *B4FZG8 | GeBP transcription factor | Phosphorylation level |  |  | 2.975 | 0.000 |
|  |  | Protein abundance | 0.989 | 0.922 | 1.024 | 0.689 |
| *B6SGQ1 | transposon protein | Phosphorylation level | 0.880 | 0.558 | 0.526 | 0.000 |
|  |  | Protein abundance | 0.997 | 0.979 | 0.911 | 0.119 |
| *B6SSS0 | transposon protein mutator sub-class | Phosphorylation level | 0.996 | 0.979 | 1.855 | 0.000 |
|  |  | Protein abundance | 0.915 | 0.432 | 1.072 | 0.240 |
| B6SWQ9 | dentin sialophospho | Phosphorylation level |  |  | 0.585 | 0.000 |
|  |  | Protein abundance | 0.915 | 0.432 | 1.058 | 0.341 |
| B6T927 | formin-like protein 7-like | Phosphorylation level | 1.229 | 0.343 | 1.797 | 0.000 |
|  |  | Protein abundance | 0.981 | 0.865 | 0.928 | 0.211 |
| *B6U0Y9 | atp binding protein | Phosphorylation level | 1.546 | 0.060 | 1.594 | 0.001 |
|  |  | Protein abundance | 1.221 | 0.088 | 1.024 | 0.689 |
| *B6U4I4 | microtubule-associated protein map65-1a | Phosphorylation level |  |  | 1.775 | 0.000 |
|  |  | Protein abundance | 0.906 | 0.383 | 0.980 | 0.735 |
| *B6UBN4 | j domain-containing protein required for chloroplast accumulation response 1-like isoform x2 | Phosphorylation level |  |  | 1.952 | 0.000 |
|  |  | Protein abundance | 1.060 | 0.618 | 0.985 | 0.800 |
| *B6UH65 | zinc transporter 2 precursor | Phosphorylation level | 0.665 | 0.069 | 3.031 | 0.000 |
|  |  | Protein abundance | 1.101 | 0.410 | 0.987 | 0.827 |
| B7ZYR5 | tpa: leucine-rich repeat receptor-like protein kinase family protein | Phosphorylation level | 1.000 | 0.996 | 1.574 | 0.003 |
|  |  | Protein abundance | 1.017 | 0.885 | 1.069 | 0.260 |
| *C0P5C4 | abc1 family protein | Phosphorylation level | 0.754 | 0.001 | 1.622 | 0.000 |
|  |  | Protein abundance | 1.153 | 0.223 | 1.080 | 0.194 |
| *C0P9I5 | tbc1 domain family member 15-like | Phosphorylation level | 1.060 | 0.197 | 2.381 | 0.000 |
|  |  | Protein abundance | 0.888 | 0.294 | 1.068 | 0.267 |
| *C0PHB9 | probable receptor-like protein kinase at5g56460-like | Phosphorylation level | 1.227 | 0.039 | 1.580 | 0.002 |
|  |  | Protein abundance | 1.111 | 0.368 | 1.003 | 0.960 |
| *G3K3T2 | guanine-nucleotide-exchange protein | Phosphorylation level |  | 1.000 | 1.725 | 0.000 |
|  |  | Protein abundance | 1.002 | 0.986 | 1.008 | 0.893 |
| K7TUB2 | hypothetical protein ZEAMMB73_551737 | Phosphorylation level |  | 1.000 | 1.558 | 0.001 |
|  |  | Protein abundance | 0.915 | 0.432 | 1.001 | 0.987 |
| K7TVQ3 | thioredoxin domain-containing protein 9 homolog | Phosphorylation level |  | 1.000 | 2.653 | 0.000 |
|  |  | Protein abundance | 1.078 | 0.521 | 0.915 | 0.137 |
| ***K7UCK7** | zinc finger ccch type domain-containing protein zfn-like 6 | Phosphorylation level | 0.976 | 0.976 | 0.655 | 0.002 |
|  |  | Protein abundance | **0.703** | **0.002** | **0.978** | **0.710** |
| *K7UVS4 | autophagy-related protein 18h-like isoform x2 | Phosphorylation level |  | 1.000 | 2.754 | 0.000 |
|  |  | Protein abundance | 0.877 | 0.246 | 1.077 | 0.210 |
| *K7V4S4 | dentin sialophospho | Phosphorylation level | 0.931 | 0.740 | 0.655 | 0.002 |
|  |  | Protein abundance | 0.858 | 0.176 | 1.001 | 0.987 |
| *K7V792 | splicing factor 3b subunit 1-like isoform x1 | Phosphorylation level |  | 1.000 | 1.755 | 0.000 |
|  |  | Protein abundance | 1.030 | 0.800 | 1.005 | 0.933 |
| *K7VDA6 | glycosyltransferase | Phosphorylation level | 0.791 | 0.317 | 0.626 | 0.001 |
|  |  | Protein abundance | 0.994 | 0.958 | 1.192 | 0.003 |
| *K7VF00 | cell division cycle and apoptosis regulator protein 1 | Phosphorylation level | 0.958 | 0.825 | 1.652 | 0.001 |
|  |  | Protein abundance | 0.928 | 0.509 | 1.019 | 0.751 |
| *K7VGC6 | cyclin-dependent kinase family protein | Phosphorylation level | 1.230 | 0.401 | 0.649 | 0.001 |
|  |  | Protein abundance | 1.034 | 0.775 | 0.968 | 0.586 |
| *K7VKP3 | sap domain containing expressed | Phosphorylation level | 1.222 | 0.147 | 1.542 | 0.004 |
|  |  | Protein abundance | 0.932 | 0.534 | 1.059 | 0.333 |
| K7VLM3;B4FHS7 | epidermal growth factor receptor substrate 15-like 1-like isoform x1 | Phosphorylation level | 1.535 | 0.067 | 1.512 | 0.005 |
|  |  | Protein abundance | 1.074 | 0.541 | 0.983 | 0.774 |
| *P05022 | atp synthase cf1 alpha subunit | Phosphorylation level | 1.160 | 0.446 | 2.574 | 0.000 |
|  |  | Protein abundance | 1.094 | 0.442 | 0.886 | 0.043 |
| P22275 | tubulin alpha chain | Phosphorylation level |  | 1.000 | 0.417 | 0.000 |
|  |  | Protein abundance | 0.983 | 0.880 | 0.987 | 0.827 |
| *P31927 | sucrose-phosphate synthase | Phosphorylation level | 1.455 | 0.105 | 1.943 | 0.000 |
|  |  | Protein abundance | 1.002 | 0.986 | 0.985 | 0.800 |
| Q41790 | calcium-dependent protein kinase | Phosphorylation level | 1.133 | 0.020 | 1.575 | 0.003 |
|  |  | Protein abundance | 0.851 | 0.154 | 1.054 | 0.374 |
| *Q8H6B1 | fact complex subunit spt16 | Phosphorylation level | 0.779 | 0.268 | 1.867 | 0.000 |
|  |  | Protein abundance | 1.064 | 0.596 | 1.015 | 0.801 |

**Table S6︱Map name of induced pathways by osmotic stress in maize *Vp***5

| **Map ID** | **Map name** | **Query sequence** | **Number** |
| --- | --- | --- | --- |
| ko03040 | Spliceosome | K7V792 C0HIN5 B6U3A0 M1GS93 B4FUX9 K7TTT8 C0P8S9 B4FX58 Q8W149 C0PMQ0 B6SY05 B4FQ73 K7VZN2 | 13 |
| ko01200 | Carbon metabolism | B7ZYP6 B8A367 C0HHU2 B6TS38 C0HIV2 B4FRM3 B4FZ38 C0PKN2 P04711 | 9 |
| ko01230 | Biosynthesis of amino acids | B8A367 C0HHU2 B6TS38 C0HIV2 B4FRM3 B4FWX5 C0PKN2 | 7 |
| ko03013 | RNA transport | M1H548 K7V1I2 B4FKD1 C0PL59 B4FX58 B6T7C2 K7V0H3 | 7 |
| ko03015 | mRNA surveillance pathway | M1H548 K7V1I2 B8A134 B4FK28 B8A305 C4J0D7 B4FX58 | 7 |
| ko00680 | Methane metabolism | C0HHU2 C0HIV2 B4FRM3 B4FZ38 C0PKN2 P04711 | 6 |
| ko00710 | Carbon fixation in photosynthetic organisms | B7ZYP6 B6TS38 B4FRM3 B4FZ38 P04711 | 5 |
| ko04120 | Ubiquitin mediated proteolysis | B4FHK6 K7TFK8 K7V4D9 B6UEN7 | 4 |
| ko04151 | PI3K-Akt signaling pathway | K7TWA4 K7TL05 O04014 P49106 | 4 |
| ko00010 | Glycolysis / Gluconeogenesis | C0HHU2 B4FRM3 B4FZ38 | 3 |
| ko00030 | Pentose phosphate pathway | B6TS38 B4FRM3 B4FZ38 | 3 |
| ko00195 | Photosynthesis | C0PNN7 P24993 B4FAW3 | 3 |
| ko00260 | Glycine, serine and threonine metabolism | C0HHU2 C0HIV2 C0PKN2 | 3 |
| ko00500 | Starch and sucrose metabolism | K7TZ83 P31927 B6T0F0 | 3 |
| ko03010 | Ribosome | B6TPG2 B4FCE7 O04014 | 3 |
| ko04075 | Plant hormone signal transduction | Q6JN48 B7ZXP0 B6THF5 | 3 |
| ko04110 | Cell cycle | K7TL05 K7VGC6 P49106 | 3 |
| ko00051 | Fructose and mannose metabolism | B4FRM3 B4FZ38 | 2 |
| ko00190 | Oxidative phosphorylation | K7VBC2 C0PNN7 | 2 |
| ko00310 | Lysine degradation | C0HHJ0 B8A298 | 2 |
| ko00620 | Pyruvate metabolism | B7ZYP6 P04711 | 2 |
| ko00720 | Carbon fixation pathways in prokaryotes | B7ZYP6 P04711 | 2 |
| ko02010 | ABC transporters | K7V8M7 Q6UNK5 | 2 |
| ko04144 | Endocytosis | C0HE93 C4J1U3 | 2 |
| ko04150 | mTOR signaling pathway | K7TWA4 O04014 | 2 |
| ko04626 | Plant-pathogen interaction | B6T6R3 B6TEF1 | 2 |
| ko04712 | Circadian rhythm - plant | B6UEN7 B6UEP1 | 2 |
| ko00196 | Photosynthesis - antenna proteins | B6STN4 | 1 |
| ko00270 | Cysteine and methionine metabolism | B8A367 | 1 |
| ko00561 | Glycerolipid metabolism | B8A0C6 | 1 |
| ko00564 | Glycerophospholipid metabolism | B8A0C6 | 1 |
| ko00630 | Glyoxylate and dicarboxylate metabolism | C0HIV2 | 1 |
| ko00670 | One carbon pool by folate | C0HIV2 | 1 |
| ko00760 | Nicotinate and nicotinamide metabolism | B6SWV6 | 1 |
| ko00770 | Pantothenate and CoA biosynthesis | B4FWX5 | 1 |
| ko00860 | Porphyrin and chlorophyll metabolism | B4FSE2 | 1 |
| ko00920 | Sulfur metabolism | B8A367 | 1 |
| ko01210 | 2-Oxocarboxylic acid metabolism | B4FWX5 | 1 |
| ko03022 | Basal transcription factors | K7VGC6 | 1 |
| ko03050 | Proteasome | B6TE60 | 1 |
| ko03060 | Protein export | C0P9K3 | 1 |
| ko03420 | Nucleotide excision repair | K7VGC6 | 1 |
| ko04115 | p53 signaling pathway | B6UEN7 | 1 |
| ko04140 | Regulation of autophagy | Q3MQ01 | 1 |
| ko04141 | Protein processing in endoplasmic reticulum | B4FXQ4 | 1 |
| ko04142 | Lysosome | K7VBC2 | 1 |
| ko04146 | Peroxisome | K7V6A5 | 1 |

**Table S7︱Map name of induced pathways by osmotic stress in maize *vp***5

| **Map ID** | **Map name** | **Protein** | **Number** |
| --- | --- | --- | --- |
| ko03040 | Spliceosome | B6T2W8, K7V792, K7VKP3, Q8W149, P11143 | 5 |
| ko04151 | PI3K-Akt signaling pathway | K7TWA4, B6SGQ1, K7UQ69, O04014 | 4 |
| ko00195 | Photosynthesis | B6T9S5, P05022, P24993 | 3 |
| ko03010 | Ribosome | B4FCK4, O04014, B4FWI0 | 3 |
| ko03013 | RNA transport | K7VKP3, B4FKD1, M1H548 | 3 |
| ko03015 | mRNA surveillance pathway | B6SGQ1, K7VKP3, M1H548 | 3 |
| ko04110 | Cell cycle | K7UQ69, B4FZM4, K7VGC6 | 3 |
| ko04141 | Protein processing in endoplasmic reticulum | C4XVE1, B6T6P7, P11143 | 3 |
| ko00500 | Starch and sucrose metabolism | Q9ZTL2, P31927 | 2 |
| ko00710 | Carbon fixation in photosynthetic organisms | B4FQ59, P04711 | 2 |
| ko01200 | Carbon metabolism | B4FQ59, P04711 | 2 |
| ko00906 | Carotenoid biosynthesis | B6SVI8 | 1 |
| ko04113 | Meiosis - yeast | B6SGQ1, B4FZM4 | 2 |
| ko04150 | mTOR signaling pathway | K7TWA4, O04014 | 2 |
| ko04626 | Plant-pathogen interaction | B6UHC3, Q41790 | 2 |
| ko00052 | Galactose metabolism | Q9ZTL2 | 1 |
| ko00190 | Oxidative phosphorylation | P05022 | 1 |
| ko00310 | Lysine degradation | B8A298 | 1 |
| ko00561 | Glycerolipid metabolism | B8A0C6 | 1 |
| ko00564 | Glycerophospholipid metabolism | B8A0C6 | 1 |
| ko00620 | Pyruvate metabolism | P04711 | 1 |
| ko00680 | Methane metabolism | P04711 | 1 |
| ko00720 | Carbon fixation pathways in prokaryotes | P04711 | 1 |
| ko00770 | Pantothenate and CoA biosynthesis | B4FWX5 | 1 |
| ko00906 | Carotenoid biosynthesis | B6SVI8 | 1 |
| ko01210 | 2-Oxocarboxylic acid metabolism | B4FWX5 | 1 |
| ko01230 | Biosynthesis of amino acids | B4FWX5 | 1 |
| ko02010 | ABC transporters | K7V8M7 | 1 |
| ko03022 | Basal transcription factors | K7VGC6 | 1 |
| ko03420 | Nucleotide excision repair | K7VGC6 | 1 |
| ko04111 | Cell cycle - yeast | B4FZM4 | 1 |
| ko04115 | p53 signaling pathway | B6UEN7 | 1 |
| ko04120 | Ubiquitin mediated proteolysis | B6UEN7 | 1 |
| ko04140 | Regulation of autophagy | Q3MQ01 | 1 |
| ko04144 | Endocytosis | P11143 | 1 |
